# Supplementary material for: Fission of double-membrane tubes under tension
Source: Biophys J. 2024 Oct 15;123(22):3977–96. doi: 10.1016/j.bpj.2024.10.009 (PMC11617631; doi:10.1016/j.bpj.2024.10.009)
Supplement: Document S1. Figures S1–S22 [file mmc1.pdf]

Biophysical Journal, Volume *123*

Supplemental information

Fission of double-membrane tubes under tension

Russell K.W. Spencer, Isaac Santos-Pérez, Anna V. Shnyrova, and Marcus Müller

Supplementary Information for

"Fission of Double-Membrane Tubes under Tension"

Russell K. W. Spencer[1], Isaac Santos-Perez[2], Anna V. Shnyrova[3,4], Marcus Müller[1]

[1]Institute for Theoretical Physics, Georg-August University, 37077 Göttingen, Germany
[2]Electron Microscopy and Crystallography, Center for Cooperative Research in Biosciences (CIC bioGUNE), Bizkaia Science and Technology Park Bld 800, 48160-Derio, Bizkaia, Spain
[3]Instituto Biofisika (CSIC, UPV/EHU), Barrio Sarriena, 48940 Leioa, Spain
[4]Department of Biochemistry and Molecular Biology, University of the Basque Country, 48940 Leioa, Spain

E-mail: russell.spencer@theorie.physik.uni-goettingen.de, anna.shnyrova@ehu.eus, mmueller@theorie.physik.uni-goettingen.de

OBSERVED WLM

An overview of the experimental setup as well as a post-fission stable WLM are shown in Figure S1.

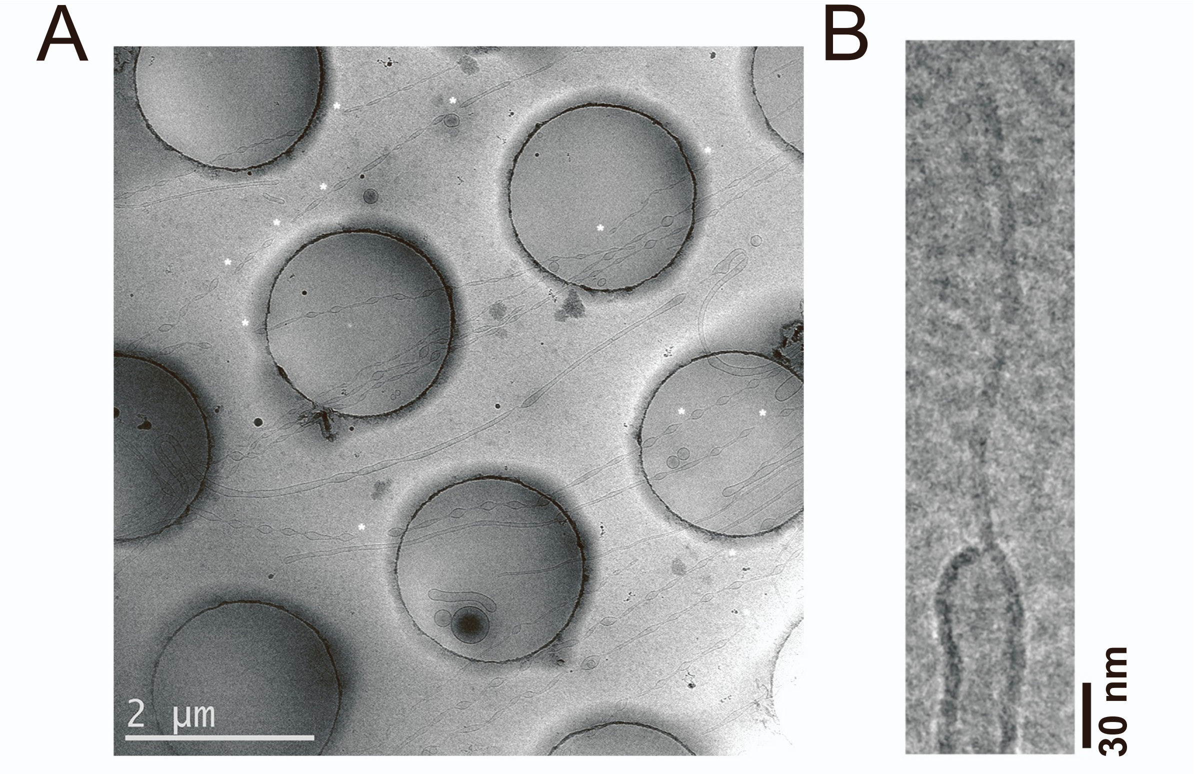

Figure S1: CryoEM of sNTs. A. Low magnification image of the sNTs obtained by the methods explained in Figure 1A. Asterisks indicate sites of sNT fission. B. Stable WLM upon sNT fission as observed by cryoEM.

PHASE BEHAVIOR

In order to study the rearrangement of lipid membranes using SCFT, we start by calculating the equilibrium phase behavior, in terms of the chemical potential of the lipids relative to the solvent, $\mu \equiv \mu_\ell - \mu_\mathrm{s}$ and composition of the lipids, f, defined as the molecular fraction of a lipid tail. The former controls the membrane tension and the latter controls the spontaneous curvature of a lipid monolayer. The phase diagram, shown in Figure S2, is calculated by comparing the grandcanonical free energies of different structures.

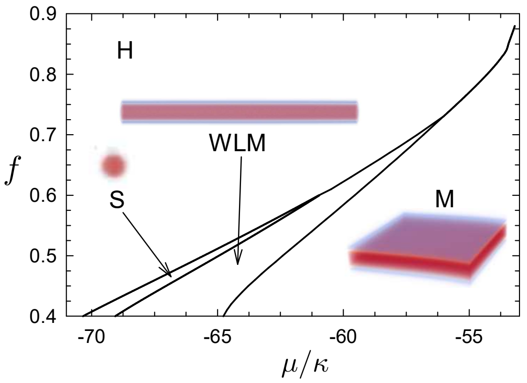

Figure S2: Phase behavior of lipids in solvent is shown in terms of the fractional composition of the lipid, f, and exchange chemical potential, $\mu = \mu_\ell - \mu_\mathrm{s}$, of the lipid relative to that of the solvent. We consider the phases: homogeneous solution (H), spherical micelles (S), cylindrical wormlike micelles (WLM); and membranes (M).

In addition to the membrane structures that interest us, we also find spherical and worm-like micelles. The free-energy calculations were performed for a single spherical micelle (S), worm-like micelle (WLM), and membrane (M), coexisting with a homogeneous solution. The curves indicate where forming a S, WLM or M first lowers the free energy at the given f and μ.

The simplest form of M is a flat planar membrane, however, other possibilities include membrane nanotubes (NTs) and vesicles. The geometry (*e.g.* NT or vesicle diameter) is controlled by additional factors, such as tension, spontaneous curvature and penalties to bending. It is also possible for multiple structures to coexist, as in the case of double-membrane nanotubes (dNTs), which will be discussed later. In this case, interactions between membranes, such as hydration repulsion, also become relevant.

Although S and WLM are no longer stable at large f, they remain metastable. The WLM-M coexistence curve meets the H-M coexistence curve at an angle and can be continued to larger f. This is to say that there is a region on the low μ side of the H-M curve where WLM have a lower free energy than membranes, though both have a higher free energy than H. A membrane under tension (lower μ than coexistence) may thus lower its free energy by converting to a WLM, before lowering the free energy further by dissolving the WLM into H.

As described in the main text, throughout this work, unless otherwise stated, we will use $\chi N = 30$ and $f = 0.8$. The constants that we derive, such as the ordering transition, bending and splay moduli as well as spontaneous curvatures are specific to these parameters.

PROPERTIES OF MEMBRANE BILAYERS AND TUBES

We are primarily interested in the mechanisms by which membranes rearrange during fission and fusion, however, it is useful to elucidate the equilibrium properties of membranes and NTs. The stability of membranes as well as the tension in the membrane can be controlled by the chemical potential of the lipids, μ. Membranes are stable at high μ, but at low μ they dissociate into a homogeneous mixed state containing a small concentration of lipids. We first locate the membrane-homogeneous transition and the relationship between the free energy per unit area, tension, in the membrane and μ. We then calculate the free energy of an NT as well as its equilibrium radius and bending energy.

The free energy of a planar membrane is shown in Figure S3. The transition occurs at $\mu_\mathrm{MH} = -11.3512\sqrt{\bar{N}}k_BT$. Expanding the free energy per unit area, $\sigma \equiv F/A$, to linear order in distance from the transition, $(\mu_\mathrm{MH} - \mu)$, we find $\sigma \approx 1.374(\mu_\mathrm{MH} - \mu)/R_0^2$ from Figure S3. The width of the bilayer at $\mu = \mu_\mathrm{MH}$, given by the distance over over which the total lipid concentration is greater than 0.5, is $d = 1.22R_0$. σ and d, along with κ discussed below, are used to scale quantities in the main paper.

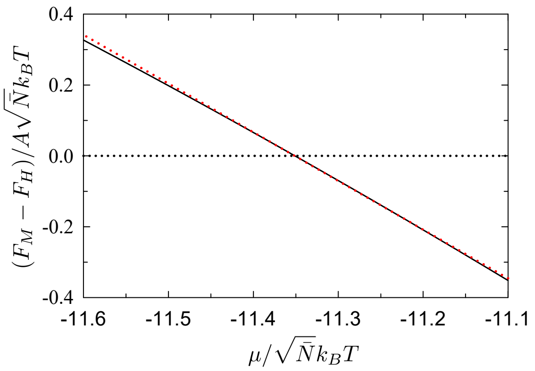

Figure S3: The free energy per unit area of a planar bilayer membrane is shown as a function of lipid chemical potential, μ. The red dotted line shows $F/A = 1.374(\mu_\mathrm{MH} - \mu)/R_0^2$.

We are primarily interested in NTs and thus turn our attention thereto. There is a number of ways of calculating the bending energy of a membrane, such as that described in Ref. 1. SCFT gives us direct access to the free energy, making our task simpler than that of simulations. The first step is to find the radius of an NT from our SCFT calculations.

We define the radius of an NT as

$$r_{\mathrm{NT}} \equiv \frac{\int d\mathbf{r}\ \phi_\ell(\mathbf{r}) r^2}{\int d\mathbf{r}\ \phi_\ell(\mathbf{r}) r} \qquad (1)$$

where $\phi_\ell(\mathbf{r})$ is the concentration of lipids at position $\mathbf{r}$ and $r = 0$ defines the cylindrical axis. We can then study the membrane bending energy which can be written as (2–4)

$$U_{\mathrm{bend}} = \int \left[\frac{\kappa}{2} (K - C_0)^2 + \bar{\kappa} K_G \right] dA \qquad (2)$$

where κ and $\bar{\kappa}$ are the bending modulus and the saddle splay modulus, respectively. The extrinsic curvature, $K = 1/R_1 + 1/R_2$, is the sum of the two principal curvatures and the Gaussian curvature, $K_G = 1/(R_1 R_2)$, is their product. The inverse length, C_0, is the spontaneous curvature. In the case of a cylinder there is only one curvature, thus $K = 1/r_{\mathrm{NT}}$ and $K_G = 0$, and it follows from the symmetry of the membrane that $C_0 = 0$. Taking the integral in Equation 2 over the area of the cylinder and adding in the, previously discussed, free energy per unit area allows us to write the free energy of a cylindrical membrane, F_{NT}, per unit length, L, as

$$\frac{F_{\mathrm{NT}}}{2\pi L} = r_{\mathrm{NT}}\sigma + \frac{\kappa}{2 r_{\mathrm{NT}}} \qquad (3)$$

where the first term is simply the free energy per unit area multiplied by the circumference of the cylinder and the second term is the bending energy.

Extracting σ from Figure S3, we then fit Equation 3 to the free energy in Figure S4A to obtain the bending modulus, $\kappa = 0.209\sqrt{\bar{N}} k_B T$. Minimizing the free energy, Equation 3, with respect to the radius results in $r_{\mathrm{NT}} = \sqrt{\kappa/2\sigma}$. Figure S4B shows a comparison with the radius found from SCFT.

Repeating this argument for a spherical vesicle of radius r_{V}, the free energy then becomes

$$\frac{F_{\mathrm{V}}}{4\pi} = r_{\mathrm{V}}^2 \sigma + 2\kappa + \bar{\kappa} \qquad (4)$$

Unlike a cylinder, a spherical vesicle cannot minimize this free energy with any finite radius. If $\sigma < 0$, the vesicle can always decrease its free energy by growing, as with a cylinder, however if $\sigma > 0$ the vesicle shrinks unbounded. This is obviously a result of truncating the bending energy expansion, as higher powers of $1/r_{\mathrm{V}}$ would become more significant as r_{V} decreases. Nonetheless, we still have a simple way of extracting the saddle splay modulus: we employ SCFT calculations similar to the ones above, but with spherical symmetry. Using $\mu \gtrsim \mu_{\mathrm{MH}}$, so $\sigma \lesssim 0$, the vesicle 'tries' to grow but is repelled by the reflecting upper boundary. This procedure produces a small correction to the r_{V}^2 term caused by the aforementioned repulsion, however the constant term, $2\kappa + \bar{\kappa}$, is unaffected, allowing us to extract $\bar{\kappa} = -0.07459\sqrt{\bar{N}}$. An example plot is shown in Figure S5.

Turning our attention back to cylinders, concentration profiles for single- and double-nanotubes, sNTs and dNTs, are

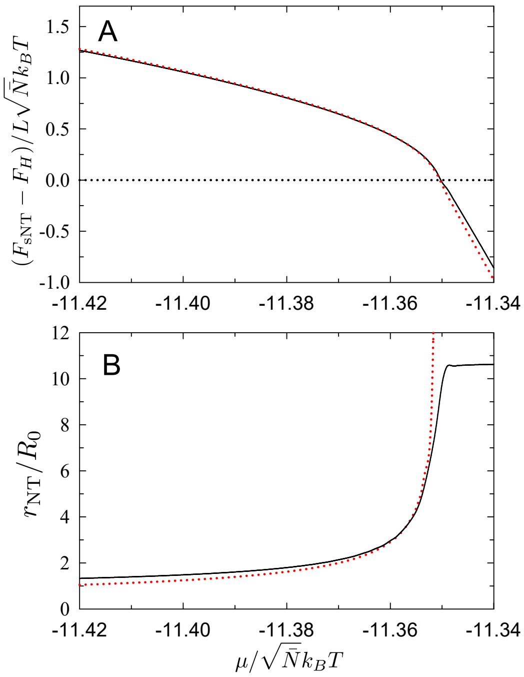

Figure S4: A Free energy of NTs per unit length and B equilibrium radii of NTs as a function of lipid chemical potential, μ. Red, dotted curves show fits to A $F/2\pi L = r_{\mathrm{NT}}\sigma + \kappa/2r_{\mathrm{NT}}$ and B $r_{\mathrm{NT}} = \sqrt{\kappa/2\sigma}$. Close to and above $\mu = \mu_{\mathrm{MH}}$ (where σ is small or negative), the radius is limited by the size of the system, and exhibits a plateau.

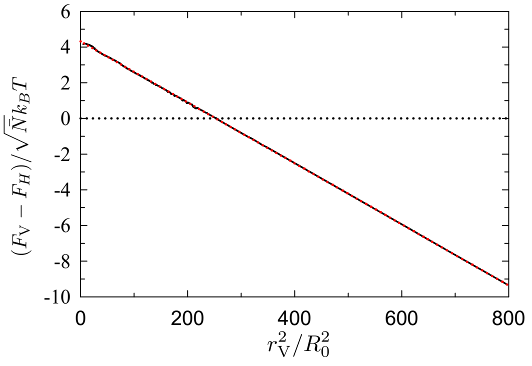

Figure S5: Free energy of a spherical vesicle as a function of radius, at $\mu = -11.35\sqrt{\bar{N}}k_BT$. The radius is controlled by a repulsion of the membrane against a reflecting boundary.

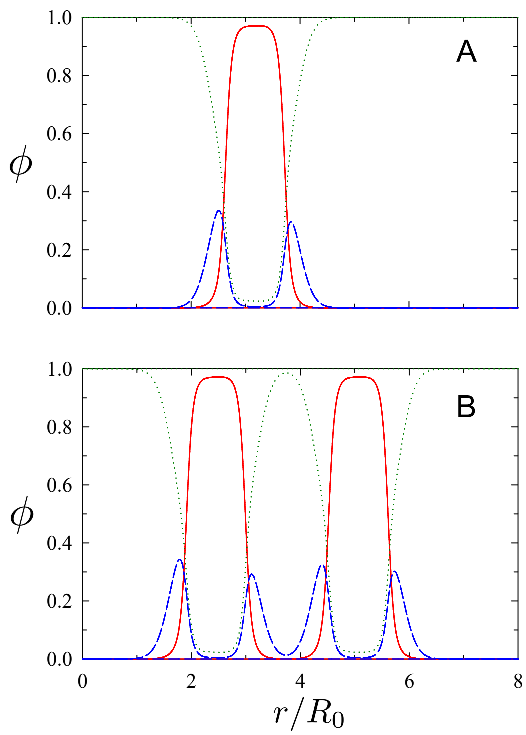

Figure S6: Profiles of A sNT and B dNT at $\mu = -11.373\sqrt{\bar{N}}k_BT$. Concentrations are shown of the (red) tail, (blue) head and (green) solvent as a function of the radial distance from the cylinder axis.

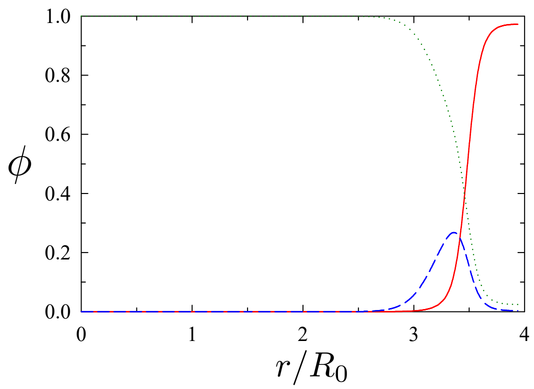

Figure S7: A planar monolayer profile is illustrated. Concentrations are shown of the (red) tail, (blue) head and (green) solvent as a function of position along the dimension normal to the membrane.

shown in Figure S6. At fixed chemical potential, NTs have a preferred radius, as discussed above. In a dNT, the radius of each is set by a combination of the preferred radius of each (that of a sNT) and the repulsion between head groups. The free energy of the dNT, relative to the mixed state, is slightly higher than double that of a sNT, due to a deviation from the equilibrium radius. Close to coexistence, however, this difference becomes small.

As we have seen, equilibrium membranes (above $\mu_{\rm MH}$) are planar (zero spontaneous curvature) and cylinders only form under tension. Throughout our calculations we are interested in the fission of NTs, as they exist in biological systems. In these cases, there are various influences, such as proteins and other cellular components, causing the membrane to retain its cylindrical shape. Close to $\mu_{\rm MH}$, we restrict the cylindrical radius simply by restricting the size of our box. This is similar to how we restricted the spherical vesicle and can also be seen in the plateau of the radius in Figure S4C, where the cylinder comes up against the reflecting boundary.

The results we have shown thus far were calculated from one-dimensional calculations, exploiting symmetries of the system. Several calculations in the main paper also use cylindrical symmetry, however, when this is not possible, as for the more complicated membrane rearrangements, we employ a cuboidal box. For the chemical potentials that we use, the square cross-section of the box has little effect. This is confirmed by the following: (i) the circular symmetry of the NTs (where expected - small deviations are noted for low tensions), (ii) recalculations where the system was initialized in rotated configurations, and (iii) comparisons between the full 3D calculation (in a cuboidal box) and the 2D (cylindrically-symmetric) calculations, where applicable. At higher μ, the cylinder pushes against the box boundaries and begins to deform, but this deformation is small (if present at all) for the values of μ presented.

PROPERTIES OF MEMBRANE MONOLAYERS

It is also instructive to examine the properties of membrane monolayers. To do this, we construct systems with profiles like those in Figure S7. Reflecting boundaries are implemented on either end of the simulation. Similar calculations can be conducted in planar, cylindrical or spherical geometries in order to examine the properties of planar, cylindrical and spherical monolayers.

Unlike bilayers, monolayers are not symmetric, and we cannot say *a priori* that the spontaneous monolayer curvature is $C_{0,m} = 0$. For the case of a cylindrical monolayer, the free energy per unit length becomes

$$\frac{F_{{\rm NT},m}}{2\pi L} = r_{\rm NT}\tilde{\sigma}_m + \frac{\kappa_m r_{\rm NT}}{2}\left(\frac{1}{r_{\rm NT}} - C_{0,m}\right)^2 \tag{5}$$

$$= r_{\rm NT}\sigma_m + \frac{\kappa_m}{2r_{\rm NT}} - C_{0,m}\kappa_m \tag{6}$$

where $\tilde{\sigma}_m$ is the free energy per unit area of the monolayer, $\sigma_m = \tilde{\sigma}_m + \kappa_m C_{0,m}^2/2$ is the free energy per unit area of a planar monolayer and κ_m is the monolayer bending modulus. The free energy of a spherical monolayer with radius $r_{\rm V}$ is

$$\frac{F_{{\rm V},m}}{4\pi} = r_{\rm V}^2\tilde{\sigma}_m + \frac{\kappa_m r_{\rm V}^2}{2}\left(\frac{2}{r_{\rm V}} - C_{0,m}\right)^2 + \bar{\kappa}_m \tag{7}$$

$$= r_{\rm V}^2\sigma_m + 2\kappa_m - 2\kappa_m C_{0,m} r_{\rm V} + \bar{\kappa}_m \tag{8}$$

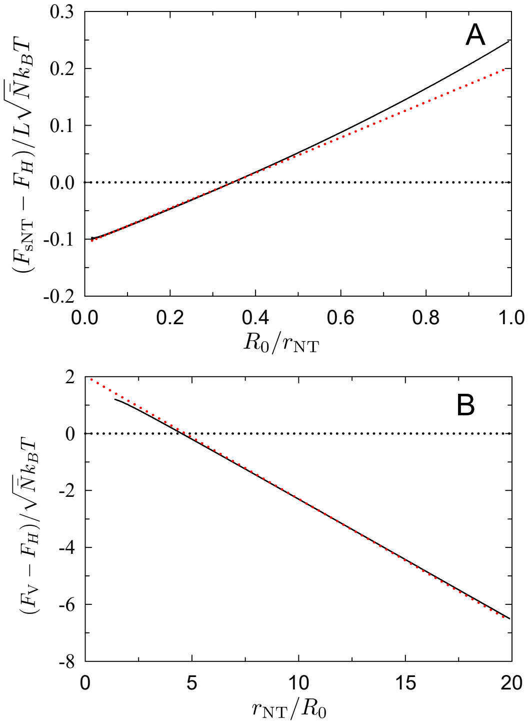

Figure S8: Free energies are shown for A cylindrical and B spherical monolayers as functions of radius, with $\mu = \mu_{\mathrm{MH}}$ chosen so that the free energy density of a planar membrane is $\sigma_m = 0$. Red curves illustrate fits to A Equation 6 and B Equation 8.

where $\bar{\kappa}_m$ is the monolayer saddle splay modulus.

Similar to our previous calculations, we examine the free energy of cylindrical and spherical monolayers. In this case, we control the radius of the cylindrical or spherical structures through our choice of the system radius. The resulting free energies are shown in Figure S8. Fitting the curves to Equation 6 and Equation 8 allows us to extract the monolayer bending and saddle splay moduli, $\kappa_m = 0.09909\sqrt{\bar{N}}k_BT$ and $\bar{\kappa}_m = -0.03741\sqrt{\bar{N}}k_BT$, and the spontaneous monolayer curvature, $C_0 = 0.1734R_0^{-1}$. As one might expect, the bending and saddle splay moduli for the monolayers are close to half of that for the bilayer.

The small deviation from $\kappa = 2\kappa_m$ likely occurs because we ignored the membrane thickness, ΔR. The bending energy of a bilayer is given by the bending energy of the two monolayers *i.e.* the sum of Equation 6 for the r_{NT} of the inner and outer membranes, with $C_{0,m}$ having opposite signs for the two membranes. For a large bilayer radius, the inner and outer radii are approximately the same and we recover Equation 3 with $\kappa = 2\kappa_m$, plus a small correction of order $\Delta R^2/r_{\mathrm{NT}}^3$. Although insignificant for large radii, this correction may account for the deviation from strictly $\kappa = 2\kappa_m$ since the fits from which we extracted κ and κ_m included small radii. A similar argument applies to $\bar{\kappa}$ and $\bar{\kappa}_m$.

LEAFLETS UNDER DIFFERENT TENSION

Throughout this work, we have assumed that all membranes (and membrane leaflets) are under the same tension, fixed by their chemical potential. When using constriction due to tension as a proxy for other, more complicated, ways of constricting the membrane, this approximation is likely appropriate, but should be both noted and tested. Testing the effects of tension differentials is particularly important in systems such as mitochondria, where the inner and outer membranes are attached to different reservoirs with, presumably, in general, different tensions. A thorough investigation is beyond the scope of this manuscript, but here we lay the groundwork for simulating membranes under different tensions using SCFT.

As described above, the tension in a membrane (or leaflet) is set by the lipid chemical potential. Furthermore, the instantaneous exchange of lipids between leaflets implies that membranes in the same simulation (with a fixed lipid chemical potential) will have the same tension. Even if we introduce multiple (miscible) lipid species, with separate chemical potentials, they will instantly mix. The solution is to introduce a repulsion between the head groups of otherwise identical lipid species, thereby relegating lipids to a specific leaflet, while allowing their tail groups to mix. This was done previously in (5). Example membranes with the same and different tensions in each leaflet are shown in Figure S9. The difference in tension is visible in the asymmetry in the head-group density on either side of the membrane.

We utilize a head-head repulsion of $\chi N = 300$ in order to reduce overlap to almost zero. Note that this large χN value does not bring about the significant challenges usually seen in SCFT with large χN because there are no interfaces between repulsive heads.

It is also important to note that at a fixed chemical potential and finite head-head repulsion, there is a shift in the free energy and thus zero-tension chemical potential which, though small, is noticeable on the scale of our calculations. For $\chi N = 300$ the shift in μ_{MH} is approximately $9.9 \times 10^{-3}\sqrt{\bar{N}}k_BT$, thus the μ values in the plot below are this amount lower than the quantities above to obtain the same tension. The shift in the free energy is due to the small number of extra lipids allowed in the system due to the finite amount of mixing, and disappears as the head-head repulsion diverges.

The next steps are to repeat the sNT calculations using a different tension for the inner and outer leaflets. Preliminary calculations (not shown) suggest that the average tension dictates the radius of the composite tube, thus we anticipate only a small effect on the barrier. One can also repeat the dNT calculations using combinations of leaflets in a membrane to create the desired membrane tension. Note that this must be done carefully. If the above technique is used and apposing leaflets are of different species, then the extra repulsion may far exceed the usual hydration repulsion, and would likely preclude fusion. This investigation is left for future work.

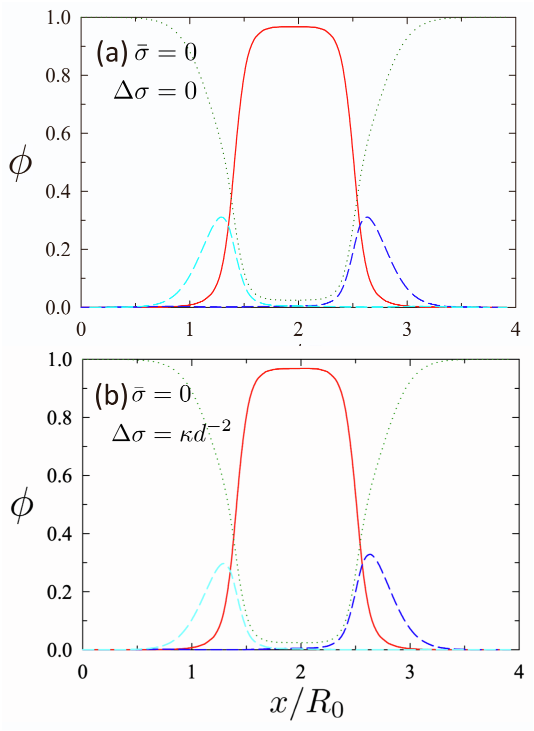

Figure S9: Profiles of planar bilayers, similar to Figure S6 and Figure S7, calculated at (a) zero tension, and (b) a total tension of zero, but the two leaflet having tensions of $\Delta\sigma = \pm\kappa/d^2$. Different head types are shown in blue and cyan.

LIPID DIFFUSION IN PLANAR MEMBRANES

In order to extract dynamical information from our particle-based simulations, we conduct simulations of planar membranes in order to compare the in-membrane self-diffusion constant of lipids to that found in experiments. An illustration of such a planar membrane and a plot of average lipid displacement is shown in Figure S10. From this we can extract a diffusion constant of approximately $D = 1.9 \cdot 10^{-4}\text{nm}^2/\Delta t$. Approximating the in-plane diffusion constant of lipids as $4\mu\text{m}^2/\text{s}$ (6) allows us to estimate a SOMA time step size of $\Delta t = 2 \cdot 10^{-10}s$. Other particle-based simulation methods, such as MARTINI (7), use time steps of $10^{-14}s$, making this a significant increase in speed. The uncertainties in both the membrane thickness (particularly between various cellular components) and lipid diffusion time are quite high, particularly as both quantities vary significantly depending on the particular membrane under consideration and the membrane environment. The resulting estimate for our time step is thus only a rough approximation. Based on the variation in the aforementioned literature values, we expect our estimate to be within a factor of 4 of the true value.

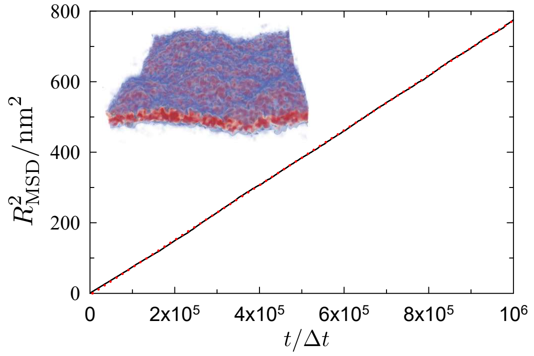

Figure S10: Mean-squared lipid displacement in the membrane plane as a function of time step, for a planar membrane in SOMA. The scaling of the vertical axis was calculated by equating an average membrane bilayer thickness of 4nm (8) with the thickness found in SCFT (see Figure S6) of approximately $1.22R_0$. The inset shows an image of the planar bilayer used in SOMA. Fitting the mean-squared displacement, ΔR^2_{MSD}, to $R^2_{\text{MSD}} = 4Dt$ yields $D = 1.9 \cdot 10^{-4}\text{nm}^2/\Delta t$, where Δt is a SOMA time step.

MEMBRANE TUBE COLLAPSE RATE

Unlike the SCFT calculations, the coarse-grained simulations can give us an order-of-magnitude estimate for the time taken to undergo the aforementioned transformation processes. Using the time step calculated above, $\Delta t = 2 \cdot 10^{-10}$ s, allows us to investigate the time taken to rearrange membranes.

The collapse of an NT into a WLM is shown in Figure S11. Approximating the membrane width as 4 nm (8) and using the previously-calculated time step, we estimate that the collapse of the NT proceeds at a rate of approximately $100\mu\text{m/s}$. At higher lipid concentration (or chemical potential), the NT has a larger diameter and collapses more slowly and the converse is also true. Repeating the above calculation, the rate of collapse appears to be proportional to the free-energy difference between sNT and WLM, which varies linearly with tension. Using the fact that $R_c = \sqrt{\kappa/2\sigma}$, we can write the speed of WLM elongation as $v = C\left(R_c^{-2} - R_{cW}^{-2}\right)$, where R_{cW} is the radius of a membrane tube at sNT-WLM coexistence. For the case outlined above, $C \approx 1.973 \cdot 10^6\text{nm}^3/\text{s}$ and $R_{cW} \approx 8\text{nm}$.

The illustrated NT is under high tension, chosen to intentionally increase the speed due to the slow speed of simulations.[1] If we were to decrease the tension so as to adjust the collapse rates in our simulations, the simulations would take prohibitively long. It should also be noted that we are approximating lipids as polymers and polymer membranes can withstand much higher tension and areal strain before rupture (9, 10) and the illustrated system is likely beyond the tension where lipid membranes would be stable.

[1]The initial condition was calculated from an NT at $\mu - \mu_{\text{MH}} = -0.26\kappa$, where the radius of the tube is $R_c \approx 3.9\text{nm}$.

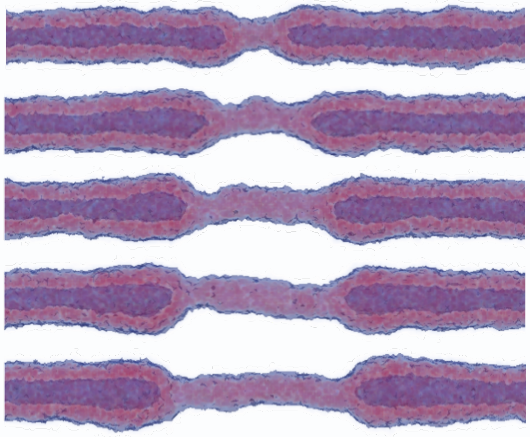

Figure S11: The collapse of a sNT into a WLM in a SOMA simulation is shown at intervals of $2 \cdot 10^5 \Delta t$. Approximating the membrane thickness as 4nm (8), the collapse proceeds at approximately $2 \cdot 10^{-5}$nm$/\Delta t$.

TRANSITION PATHWAY DETAILS

In order to clarify the pathways presented in the main text, this section reproduces, annotates and expands upon Figures 5-8 and discusses variations to the mechanisms presented in the main text. In cases where membrane changes may not be obvious, we have highlighted the relevant locations on the images.

We first consider a variation of the sNT collapse mechanism. As discussed in the main text, the minimum free energy path (MFEP) connecting an intact sNT and a pair of capped sNTs does not include pores. Seeing as short-lived pores are apparently readily nucleated by interactions between membranes in a dNT, we wished to investigate the fission mechanism of a sNT where we enforce that the initial condition contains a pore. This is illustrated in Figure S12. rather than closing the pore, and then undergoing fission in the usual way (collapse to HF, then WLM, then fission of the WLM) the pore facilitates fission by simultaneously unzipping the sNT and closing it, until there are two capped sNTs connected by a WLM, with pores in the caps, which then close.

Note that the starting state of Figure S12 necessarily involves an external field that renders the pore in the sNT metastable. Without such an external field, a pore in the sNT is *not* metastable, *i.e.* if allowed to relax, it would close and an intact sNT would form. A pore in a sNT could be rendered metastable, *e.g.* by proteins. In this case, the free-energy barrier between the sNT with a pore and the WLM is lower than that associated with a collapse of an intact sNT.

Figure S13 reproduces Figure 6A in the main text, and represents the simplest pathway for merging the inner and outer membranes in a dNT. Starting from A the unperturbed dNT, the outer membrane bends inwards towards the inner membrane (highlighted in B). A small contraction of the inner NT is also visible. C A stalk then grows between the inner and outer membranes. The membrane thins on either side of the stalk - this is first apparent in D. E-F The stalk widens horizontally and the connection thins (thins vertically, from the vantage point of the lower panels). This produces a thin hemifusion diaphragm (HD), which separates the inner and outer regions. G-H The HD may then broaden. The free-energy barrier to rupturing this HD is small, and we have observe it rupturing, to form a pore, in some particle-based simulations.

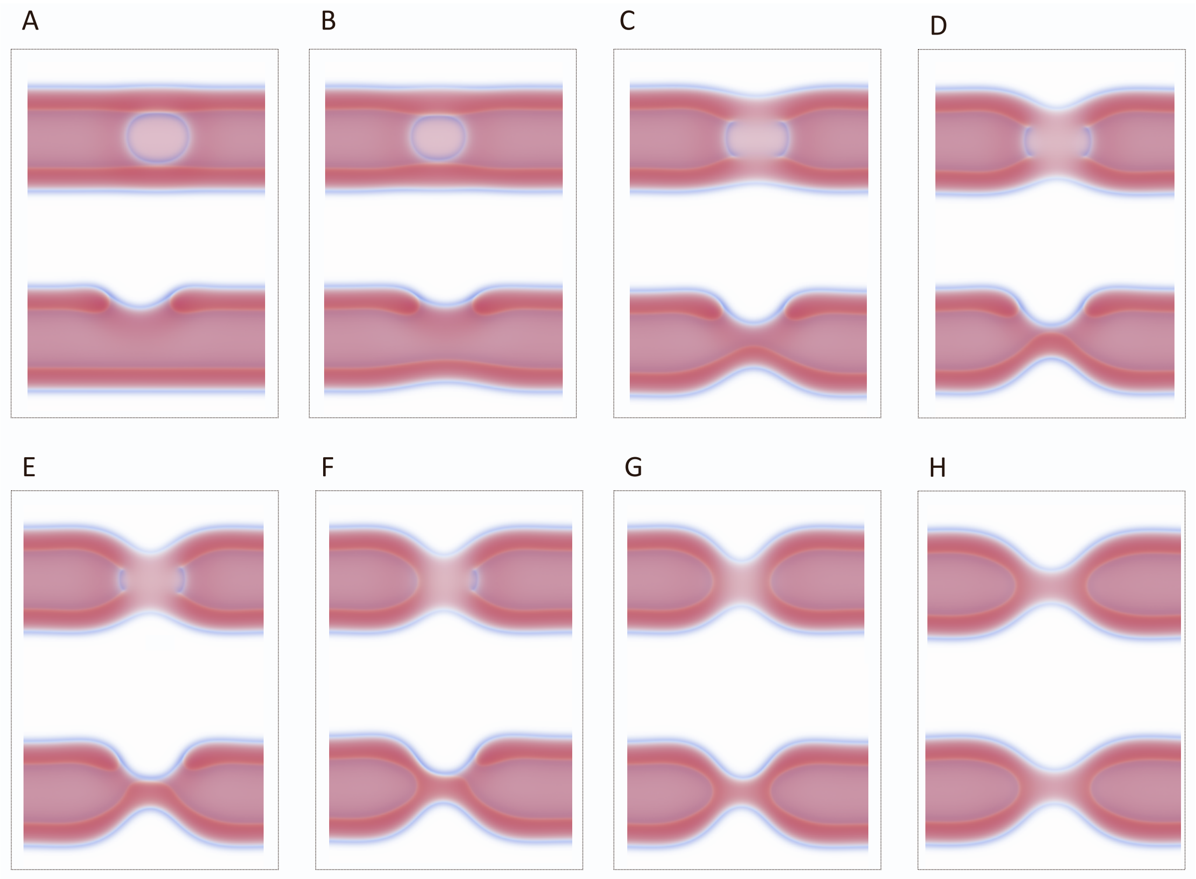

Figure S12: Images of the fission of a sNT starting from a state containing a pore at $\sigma = 0.387\kappa/d^2$.

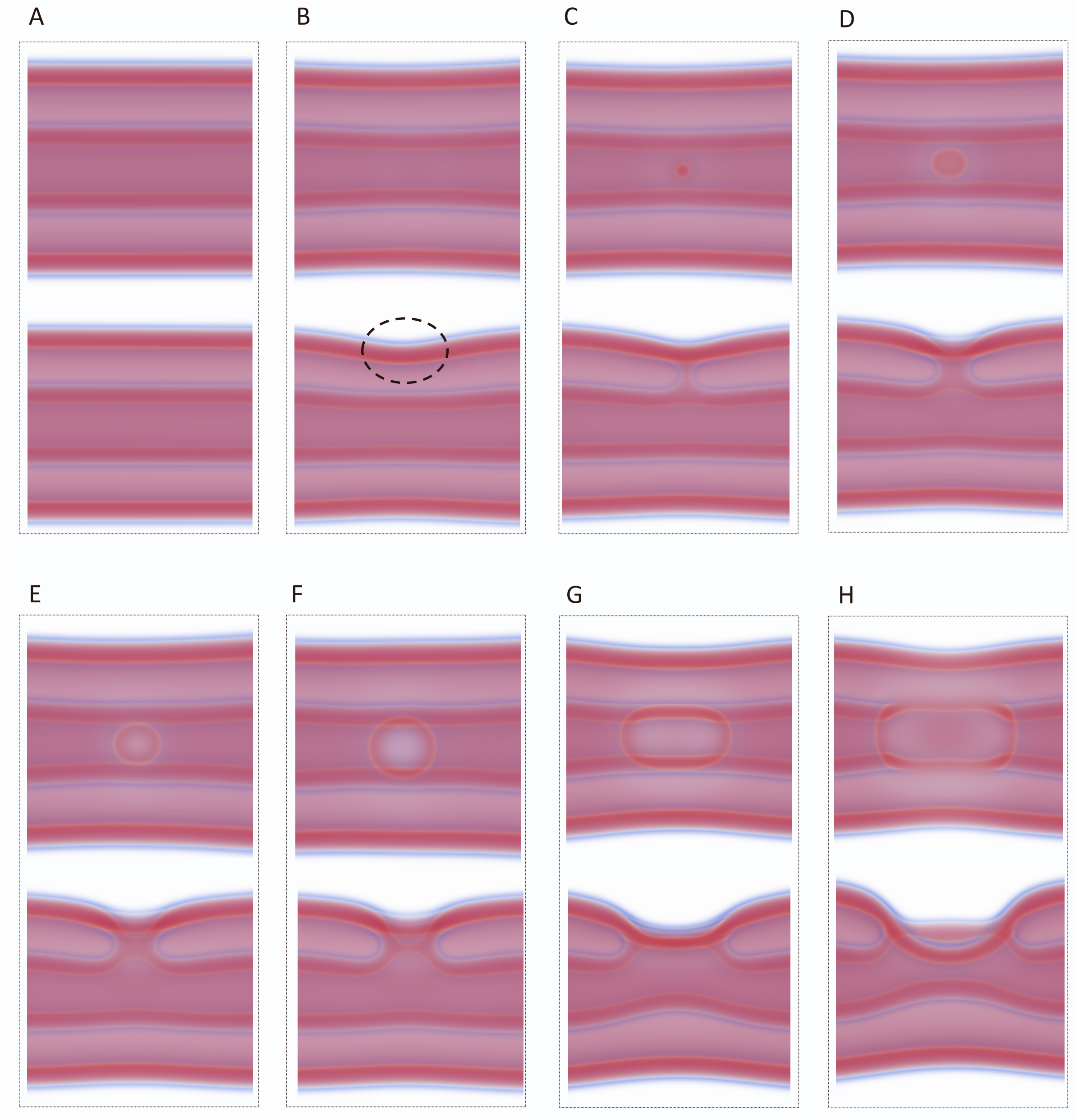

Figure S13: Enlarged versions of the images in Figure 4A in the main text. Panel A has been added and represents the unperturbed double membrane. The change in panel B has been highlighted. Unlike in the main text, outer tubes have not been made transparent.

Figure S14 reproduces Figure 6B in the main text, and represents the variation on the previous pathway, where a pore intermediate is present in the outer membrane. The transition begins similarly, bending the outer membrane towards the inner and forming a stalk. Once again, the membranes become thinner around the stalk. This time, however, a pore nucleates in D. The stalk widens when the pore forms (going from panel C to D). The stalk continues to widen, and zips around the pore. The expanding connection is highlighted in E. The next three steps, F-H, are the same as in the previous pathway.

In fact, there are more variations of this pathway. Figure S15 shows a path where a stalk forms (as in Figure S14) and nucleates a pore in the inner membrane. As before, the stalk then grows around the pore. Another variant of this, shown in Figure S16, is to first form a pore, which then causes a stalk to form next to it and subsequently zip around the pore. A similar mechanism (not shown) can occur, involving a pore first forming in the outer membrane. Other variations may involve multiple stalks, for example on either side of the pore, stalks forming at different positions *i*.e. not adjacent along the axis of the cylinder, but at different angles etc.

The barrier to each of these pathways is similar – the difference is negligible on the scale of our calculations. The difference in free-energy barrier between these pathways is so similar because of the following: the barrier (free energy difference between the peak and preceding minimum) is determined by the highest peak compared with the unperturbed dNT state. The barrier to reversing the transition (see discussion in the main text) is small along any of these paths, which is to say, the barrier height is dominated by the difference in free energy between the hemifused state and the unperturbed dNT state.

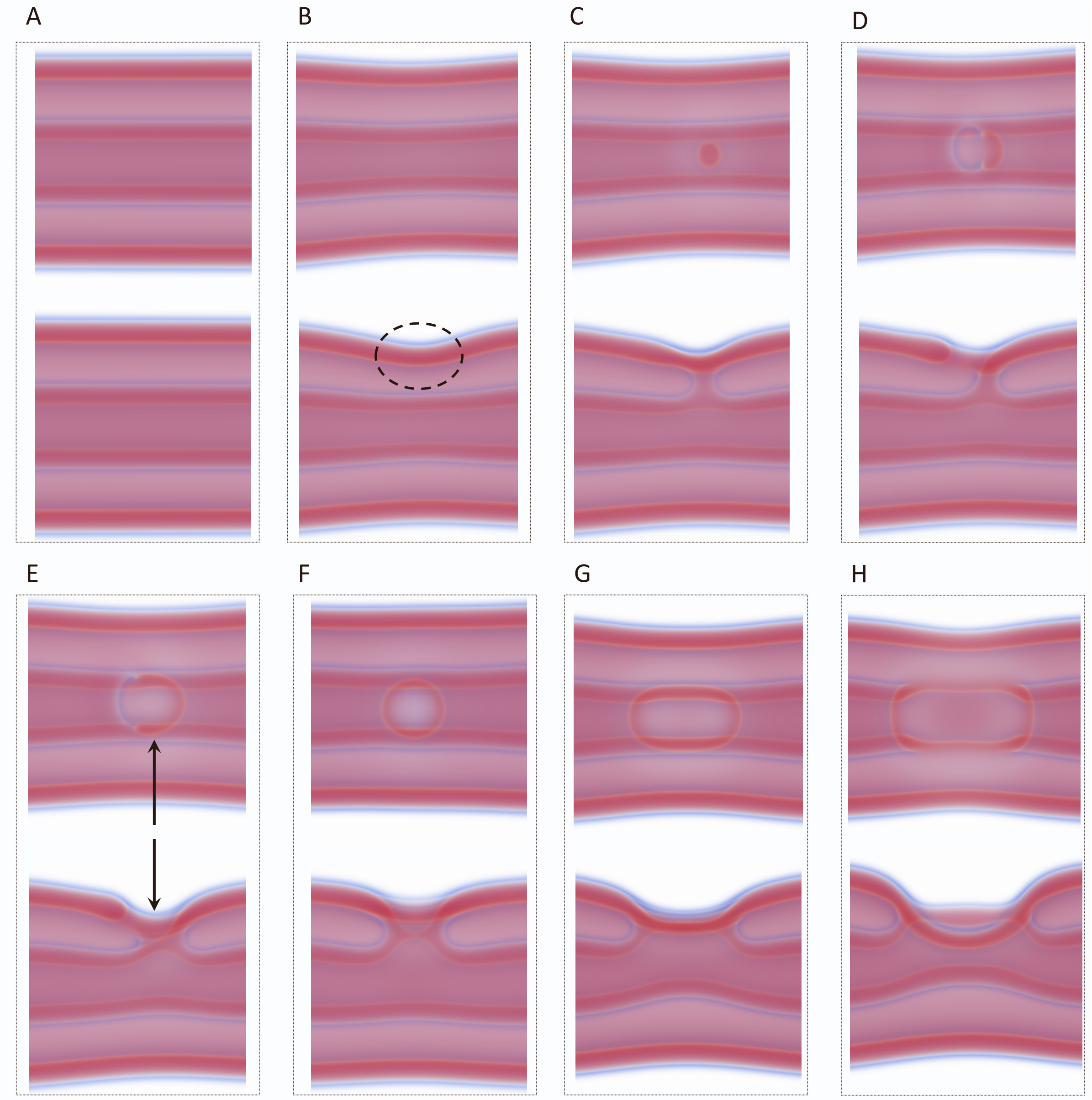

Figure S14: Enlarged versions of the images in Figure 4B in the main text. Panel A has been added and represents the unperturbed double membrane. Interesting points in panels B and E have been highlighted.

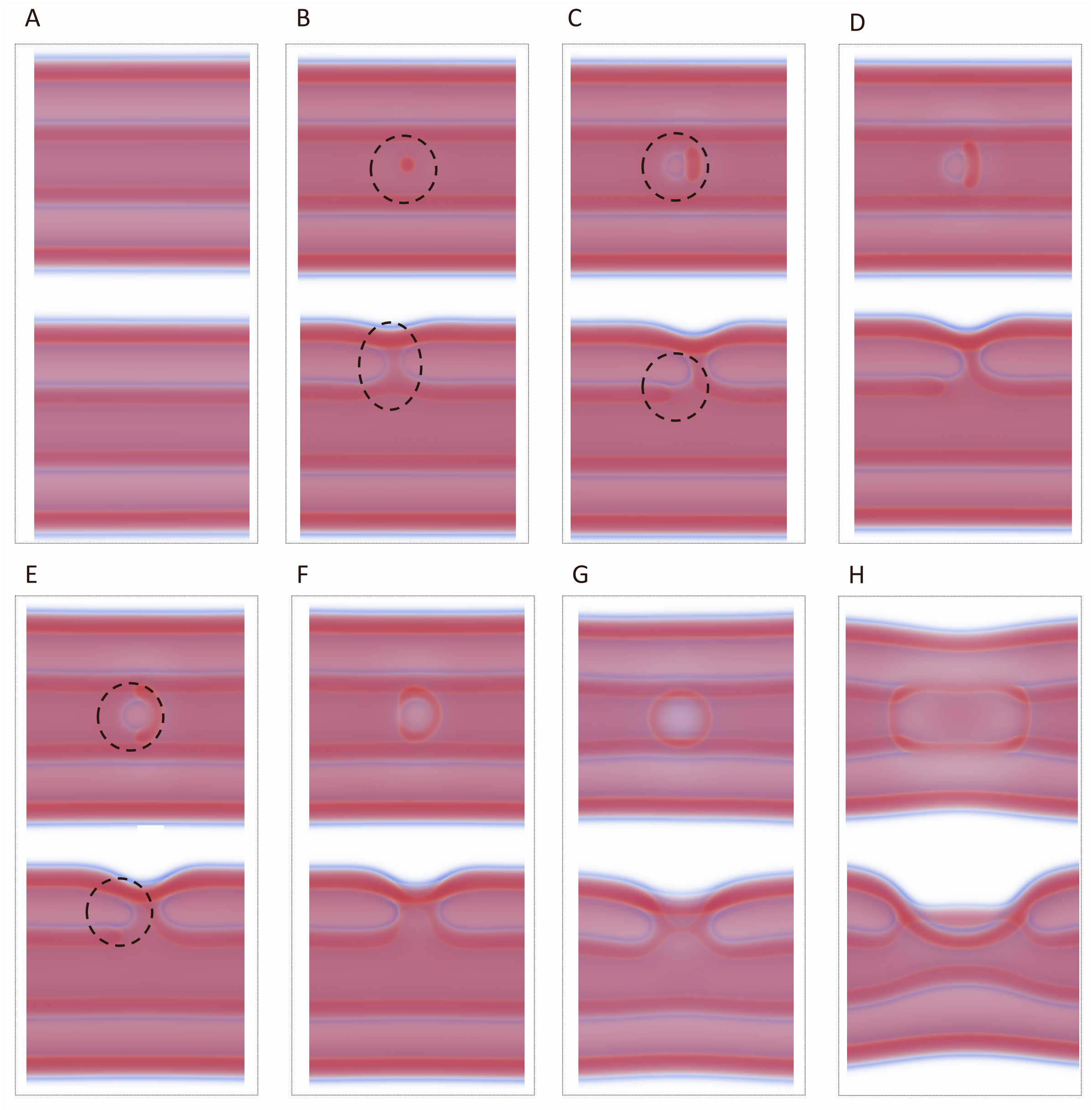

Figure S15: A path similar to Figure S14 but with a pore forming on the inner membrane, at $\sigma = 0.387\kappa/d^2$.

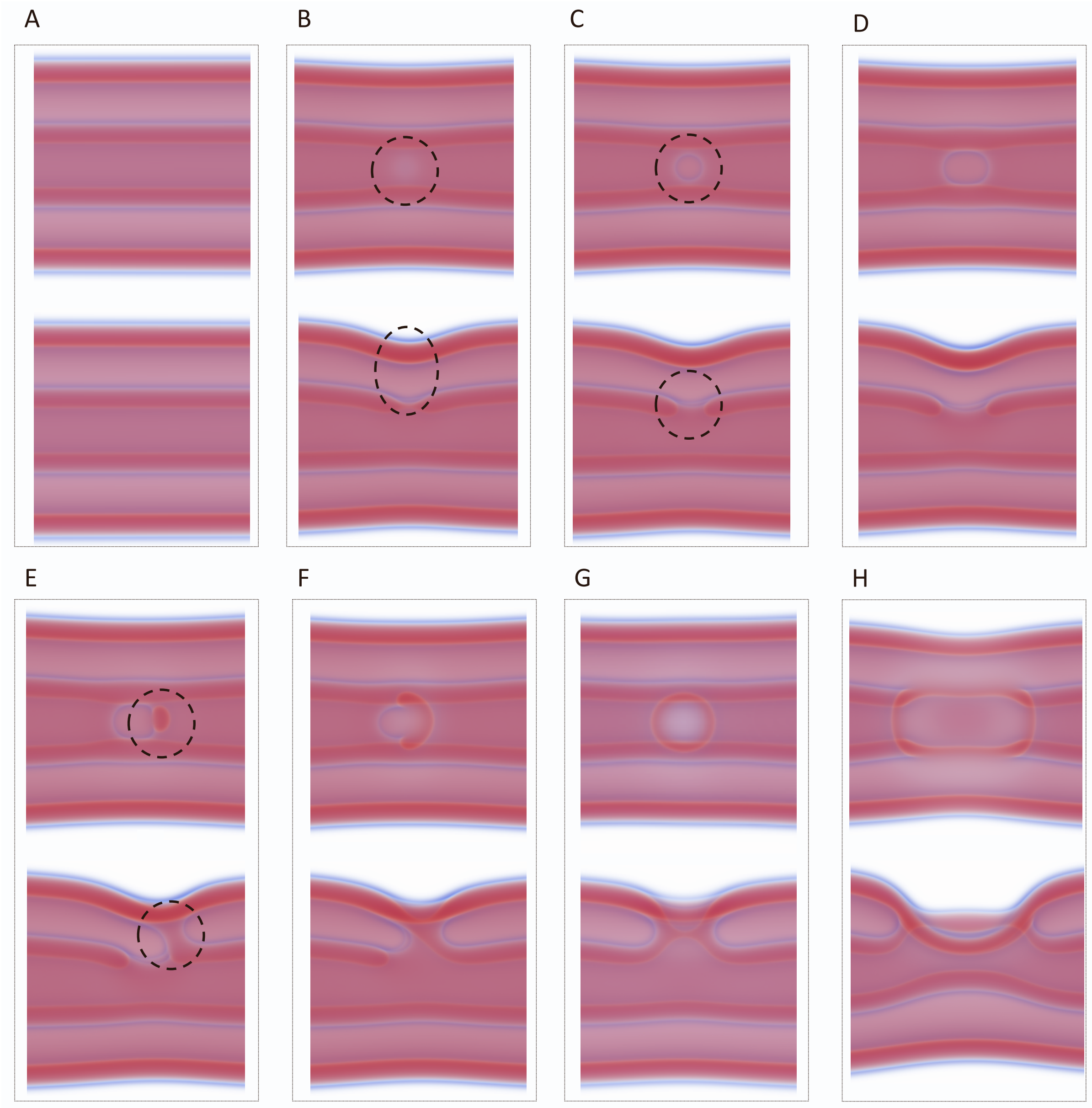

Figure S16: A path similar to Figure S15 but with the pore forming before the stalk, at $\sigma = 0.387\kappa/d^2$.

Figure S17 reproduces Figure 6D in the main text for $\sigma = 0.387\kappa/d^2$, with images added to illustrate the collapse of the inner NT and the concomitant formation of a WLM. The initial state, panel A, is the unperturbed dNT. Both NTs become thinner in panel B. As the inner NT thins, on its way to a WLM, the hydrostatic repulsion pushing on the outer NT decreases, allowing it to partially adopt a thickness closer to its equilibrium radius. In panel C the inner NT collapses, connecting to itself. The connection grows in panel D, forming a long WLM that connects two capped NTs. The growth continues and, in panel F, the outer membrane bends inward towards the WLM.

In the next phases of the evolution, depicted in panel G, a stalk forms at the saddle-point of the inward-bending outer NT. Note that the image in panel F has been rotated 90° (about the cylinder axis) relative to panel G, in order to illustrate the inward bend. The orientation is consistent in all other images. Just 'above' and 'right' of the stalk in panel G, there is another inward bend from the outer NT, and the WLM bends slightly 'right' towards the outer membrane. A second stalk forms here, as shown in panel H. In panel I this is followed by a pore forming in the inner NT. This pore connects the inner and intermediate regions, making this pathway 'leaky'.

After the stalks and pore have formed, panel J, the 'left' stalk widens, followed by the right stalk widening in panel K. The two wide stalks merge at the 'top' and then at the 'bottom', in panels L and M, respectively. The inside of the 'upper' inner NT is now once again disconnected from the intermediate region. Note that in panel M the 'lower' portion of the WLM remains a WLM. Panel H shows that this WLM shortens and widens, similar to what we saw in Figure S13, forming a diaphragm, which separates the 'upper' and 'lower' inner NTs. The diaphragm then ruptures in panel O, reconnecting the inner NTs and adopting the same final state seen in Figure S13 and Figure S14. At lower tension (higher μ) the hemifusion diaphragm is more circular. The preferred shape appears to be controlled by the curvature of the membranes.

As with the previous pathway, variations of this mechanism exist. Some features are consistent between all pathways that we have observed, *e.g.* the presence of two stalks.[2] The steps L-O, however, may occur differently, with a pore first opening in the 'lower' NT (similar to the pore in I) and the connections growing around this pore (similar to the steps K-L).

[2]As described in the main text, if one stalk is manually removed from the string, it reforms when the string is relaxed.

Figure S18 reproduces Figure 7 in the main text for $\sigma = 0.387\kappa/d^2$. The HD, shown in the final panel of Figure S13, expands around the dNT. As the HD expands, the highly-curved region highlighted in B becomes thin. This is apparent from the light color. C As the curvature increases further, a pore opens in the outer membrane and D expands. The pore closes by connecting the outer membrane to the inner membrane. This re-connection is beginning to form in E. The result is two dNTs connected by an sNT, the cylindrically hemifused state. As with the other pathways, a variation exists of this pathway, where a pore opens in the outer membrane earlier, *i.e.* close to the time represented by B. The pore then travels around the membrane until C. Once again, we showed only the variation with the lowest free energy.

Figure S19 reproduces Figure 9A in the main text. Once again, the main changes are highlighted. Starting from the cylindrically hemifused state (final state in Figure S18) B a pore forms in the inner membrane. The pore begins to 'unzip' the inner and outer membranes. As it unzips, the 'open' side of the inner NT bends inward and connects to the other side, closing the inner NT. D What remains is an inner NT, connected to the outer NT by a stalk. E The stalk ruptures.

Figure S20 reproduces Figure 9b in the main text. This time, rather than forming a pore first, the inner NT connects to itself forming an HD separating the inner region. This is highlighted in B. The formation of this HD is likely helped by the inward bend seen at the top of A. C The HD extends into a stalk, or WLM. The inner NT is now a capped NT, connected to an HD by a WLM. D The WLM ruptures, leaving the HD intact. E The HD ruptures shortly thereafter.

Figure S21 reproduces Figure 9c in the main text. A The outer membranes come closer to one another (right hand side) and B a pore forms between them. As we have seen before, the pore expands as shown in B-D, leaving only a WLM. The WLM then ruptures. There is some variation in the geometry of this mechanism (though not the topology) depending on the tension in the NT. At high tension, the sNT in the middle is longer than at low tension (See Figure 9 in the main text).

In the case of a long sNT, the outer membranes do not bend towards one another, rather a pore opens on one side of the sNT (*i.e.* close to a connection point). Once the WLM forms, it ruptures at a connection point and retracts, similar to what we saw for the fission of a WLM connecting two capped NTs. At low tension, where the sNT is short, the WLM is merely a short stalk and ruptures in the middle.

In addition to the pathways illustrated in the main text, the CH state may collapse through via the fission of the ST, as illustrated in Figure S22. The ST collapses via the canonical pathway and then retracts. Once disconnected, this state continuously transforms into two capped tubes, connected by a stalk. We expect that in experimental systems, the double tubes would retract further before this occurs, thus the barrier should be that of the canonical pathway and involve the retraction of a capped single tube. This is not shown due to the small size of our simulations box.

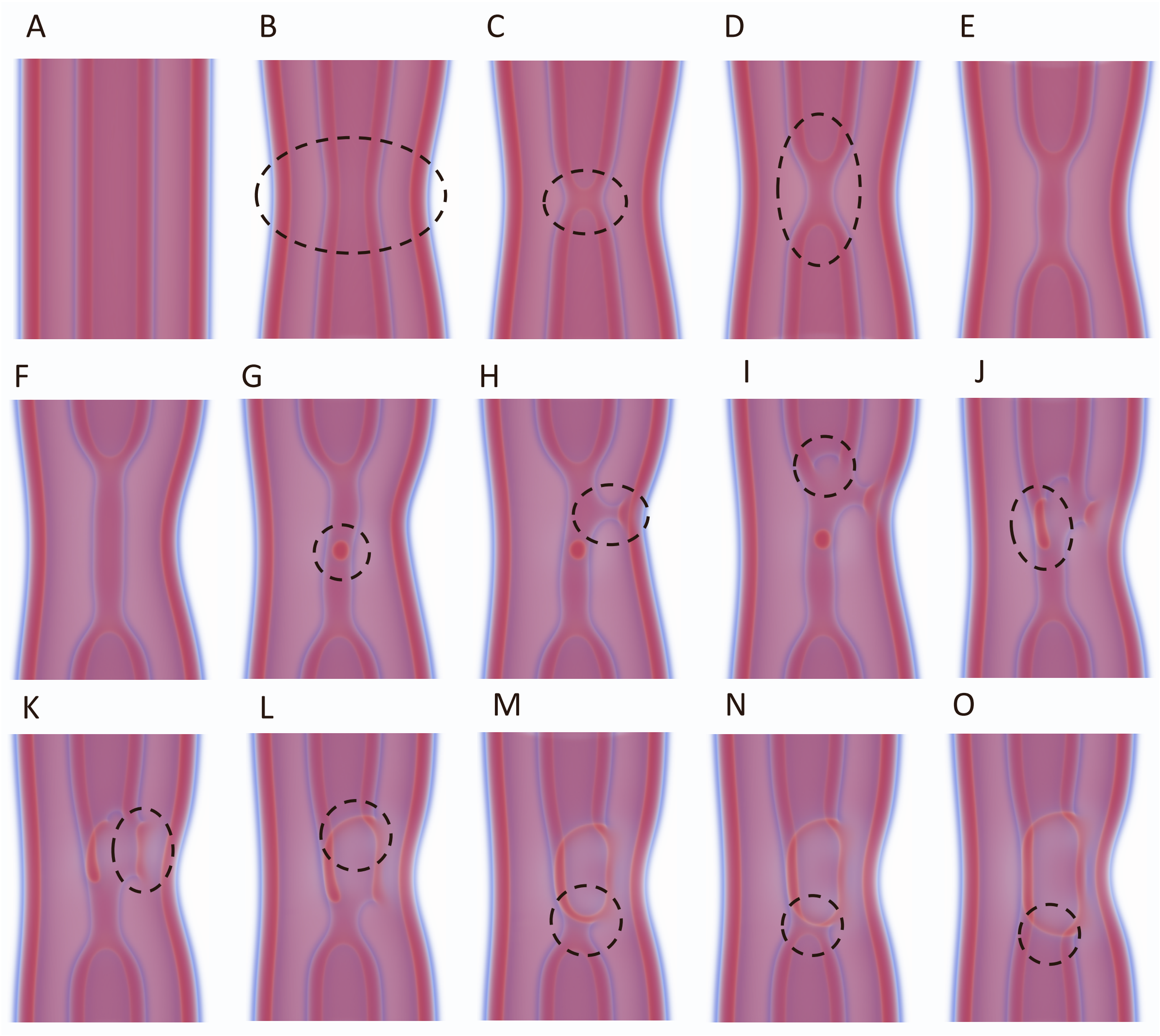

Figure S17: Enlarged versions of the images in Figure 4D in the main text. Panels A-E have been added and represent the collapse of the inner NT into a WLM. Interesting points have been highlighted to aid visualization and discussion.

REFERENCES

1. Harmandaris, V. A., and M. Deserno, 2006. A novel method for measuring the bending rigidity of model lipid membranes by simulating tethers. *Journal of Chemical Physics* 125:204905.

2. Helfrich, W., 1973. Elastic Properties of Lipid Bilayers: Theory and Possible Experiments. *Zeitschrift für Naturforschung C* 28:693–703.

3. Evans, E., 1974. Bending Resistance and Chemically Induced Moments in Membrane Bilayers. *Biophysical Journal* 14:923–931.

4. Canham, P., 1970. The minimum energy of bending as a possible explanation of the biconcave shape of the human red blood cell. *Journal of Theoretical Biology* 26:61–81.

5. Spencer, R. K., Y. G. Smirnova, A. Soleimani, and M. Müller, 2024. Transient pores in hemifusion diaphragms. *Biophysical Journal* 123:2455–2475.

6. Macháň, R., and M. Hof, 2010. Lipid diffusion in planar membranes investigated by fluorescence correlation spectroscopy. *Biochimica et Biophysica Acta (BBA) - Biomembranes* 1798:1377–1391.

7. Souza, P. C. T., R. Alessandri, J. Barnoud, S. Thallmair, I. Faustino, F. Grünewald, I. Patmanidis, H. Abdizadeh, B. M. H. Bruininks, T. A. Wassenaar, P. C. Kroon, J. Melcr, V. Nieto, V. Corradi, H. M. Khan, J. Domański, M. Javanainen, H. Martinez-Seara, N. Reuter, R. B. Best, I. Vattulainen, L. Monticelli, X. Periole, D. P. Tieleman, A. H. de Vries, and S. J. Marrink, 2021. Martini 3: a general purpose force field for coarse-grained molecular dynamics. *Nat. Methods* 18:382–388.

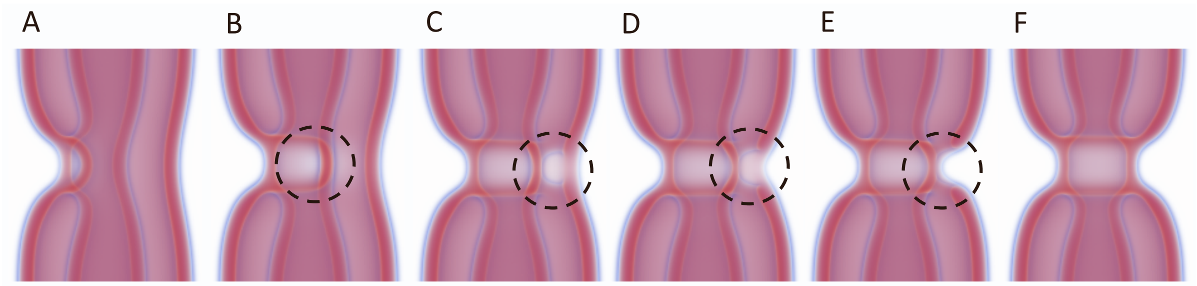

Figure S18: Enlarged versions of the images in Figure 7 in the main text. Interesting points have been highlighted to aid visualization and discussion.

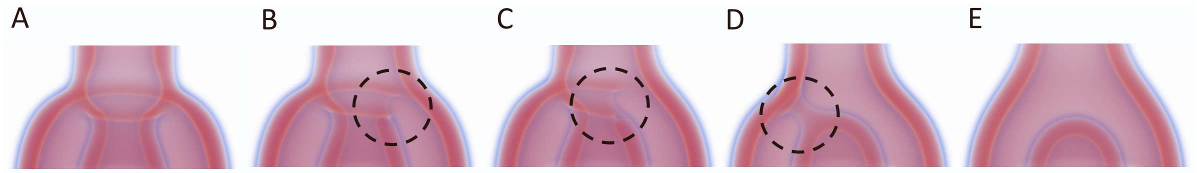

Figure S19: Enlarged versions of the images in Figure 9a in the main text. Interesting points have been highlighted to aid visualization and discussion.

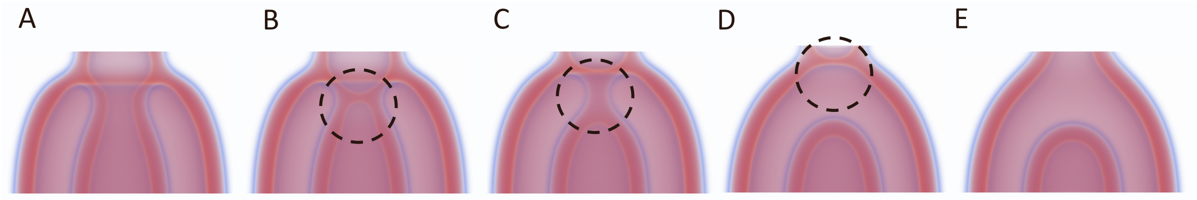

Figure S20: Enlarged versions of the images in Figure 9b in the main text. Interesting points have been highlighted to aid visualization and discussion.

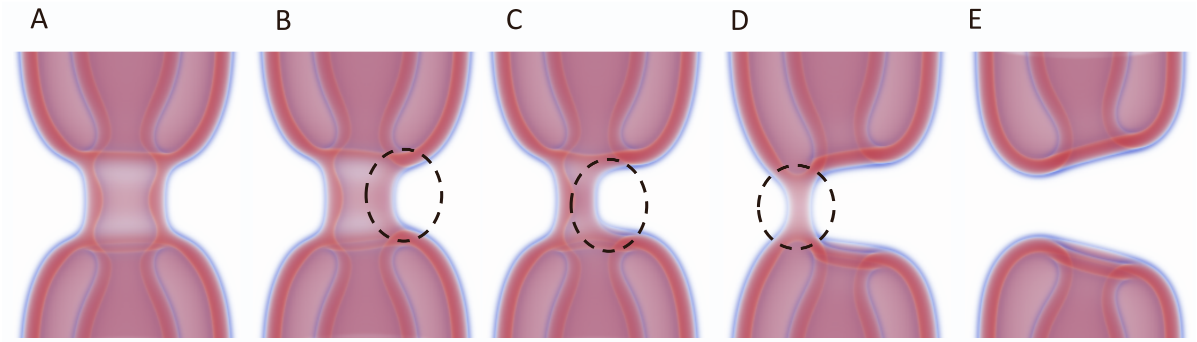

Figure S21: Enlarged versions of the images in Figure 9c in the main text. Interesting points have been highlighted to aid visualization and discussion.

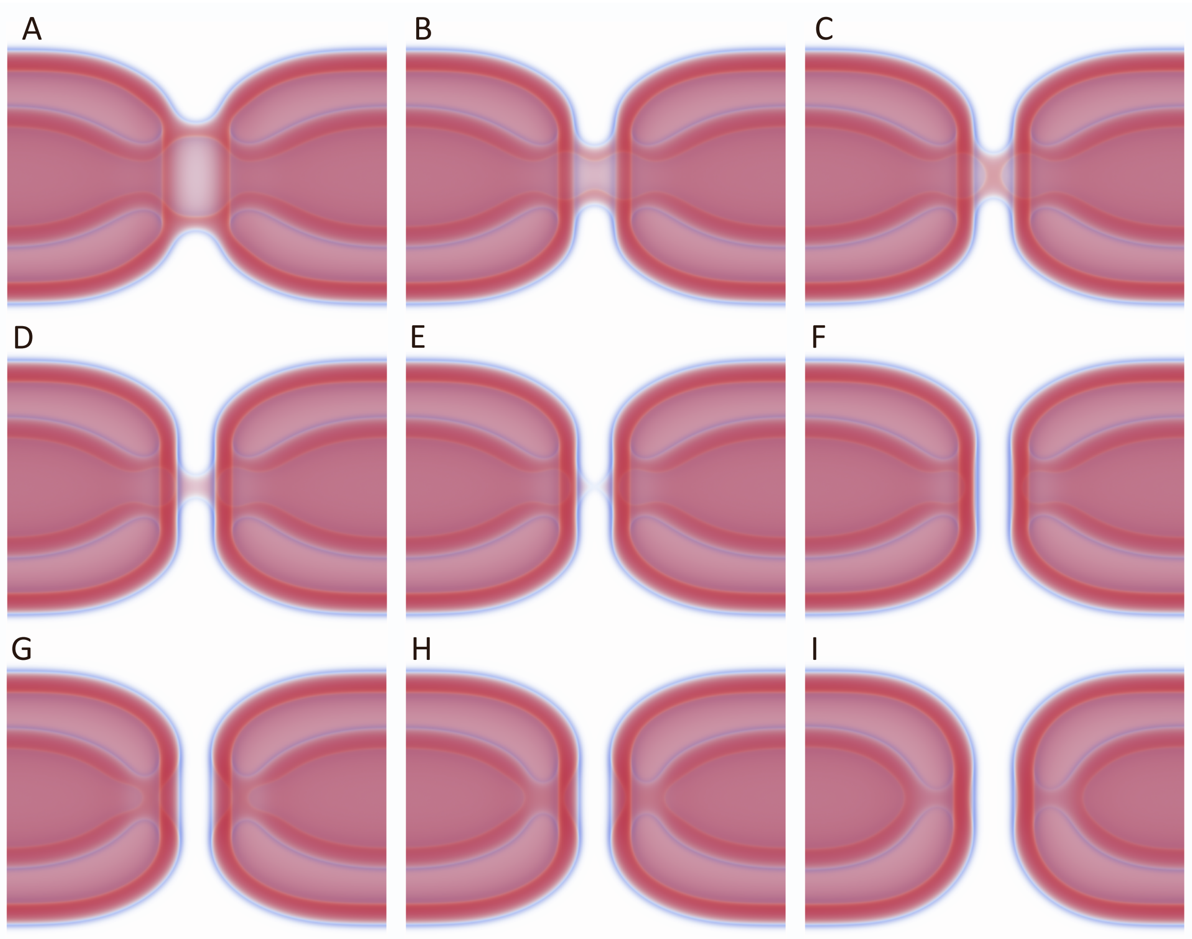

Figure S22: Collapse of the CH state via the canonical fission of the ST at $\sigma = 0.129\kappa/d^2$.

8. Mitra, K., I. Ubarretxena-Belandia, T. Taguchi, G. Warren, and D. M. Engelman, 2004. Modulation of the bilayer thickness of exocytic pathway membranes by membrane proteins rather than cholesterol. *Proceedings of the National Academy of Sciences* 101:4083–4088.

9. Discher, B. M., Y.-Y. Won, D. S. Ege, J. C.-M. Lee, F. S. Bates, D. E. Discher, and D. A. Hammer, 1999. Polymersomes: Tough Vesicles Made from Diblock Copolymers. *Science* 284:1143–1146.

10. Dimova, R., U. Seifert, B. Pouligny, S. Förster, and H.-G. Döbereiner, 2002. Hyperviscous diblock copolymer vesicles. *The European Physical Journal E* 7:241–250.
