# Supplementary material for: Fission of double-membrane tubes under tension
Source: Biophys J. 2024 Oct 15;123(22):3977–96. doi: 10.1016/j.bpj.2024.10.009 (PMC11617631; doi:10.1016/j.bpj.2024.10.009)
Supplement: Document S2. Article plus supporting material [file mmc9.pdf]

Biophysical Journal
Article

Fission of double-membrane tubes under tension

Russell K. W. Spencer,[1,*] Isaac Santos-Pérez,[2] Anna V. Shnyrova,[3,4,*] and Marcus Müller[1,*]
[1]Institute for Theoretical Physics, Georg-August University, Göttingen, Germany; [2]Electron Microscopy and Crystallography, Center for Cooperative Research in Biosciences (CIC bioGUNE), Bizkaia Science and Technology Park, Derio, Spain; [3]Instituto Biofisika (CSIC, UPV/EHU), Leioa, Spain; and [4]Department of Biochemistry and Molecular Biology, University of the Basque Country, Leioa, Spain

ABSTRACT The division of a cellular compartment culminates with the scission of a highly constricted membrane neck. Scission requires lipid rearrangements, topology changes, and transient formation of nonbilayer intermediate structures driven by curvature stress. Often, a side effect of this stress is pore-formation, which may lead to content leakage and thus breaching of the membrane barrier function. In single-membrane systems, leakage is avoided through the formation of a hemifusion (HF) intermediate, whose structure is still a subject of debate. The consequences of curvature stress have not been explored in double-membrane systems, such as the mitochondrion. Here, we combine experimental and theoretical approaches to study neck constriction and scission driven by tension in biomimetic lipid systems, namely single- and double-membrane nanotubes (sNTs and dNTs), respectively. In sNTs, constriction by high tension gives rise to a metastable HF intermediate (seen as stalk or worm-like micelle), whereas poration is universally slower in a simple neck. In dNTs, high membrane tension causes sequential rupture of each membrane. In contrast, low tension leads to the HF of both membranes, which may lead to a leaky fusion pathway, or may progress to further fusion of the two membranes along a number of transformation pathways. These findings provide a new mechanistic basis for fundamental cellular processes.

SIGNIFICANCE Biological membranes serve as barriers, delimiting cellular compartments. A key feature of cellular membranes is their ability to change topology through transient remodeling events, fusion or fission, without mixing the compartments' contents. This work investigates the mechanisms and transformation pathways of membrane fission, particularly double-membrane fission as it occurs in mitochondrial division, focusing on the structure and thermodynamics of intermediate states. We uncover a complex landscape of possible membrane rearrangements during fission, some of which may be deleterious to mitochondria. These developments shed light on fission mechanisms but also open questions about how organelles select from a multitude of possible rearrangement pathways.

INTRODUCTION

Membrane fission, a process key to cellular membrane dynamics (1), occurs at the later stages of organelle division when one membrane compartment becomes two. From the physiological point of view, this process should proceed without the loss of contents enclosed by the membrane, preserving the membrane's barrier function. Therefore, membrane fission is assumed to be a highly localized and controlled cellular process, with phospholipids having the leading role in the leakless remodeling process due to their intrinsic polymorphism (2). Indeed, the essence of membrane fission can be distilled to a tiny lipid neck linking the dividing compartments. In steady state and assuming cylindrical geometry, the neck's radius is related to the membrane tension, σ, by:

$$R_c = \sqrt{\frac{\kappa}{2\sigma}} \tag{1}$$

where κ is the bending rigidity set by the membrane composition, and the membrane tension, σ, is dictated by the available membrane reservoir (3). To undergo fission, the neck should first be constricted to a critical radius in the order of magnitude of the thickness of a lipid monolayer (4,5). At this point, a spontaneous lipid rearrangement occurs, leading to the neck's scission due to the accumulated curvature stress (2). However, such membrane constriction is hardly achievable in free-standing lipid systems, where κ is in the range of a few tens of $k_{\rm B}T$, whereas $\sigma < 10\ {\rm mN/m} \approx 2.4\ k_BT/{\rm nm}^2$. Therefore, the process does not occur spontaneously, but cells use proteins to drive fission in a controlled way.

Submitted June 24, 2024, and accepted for publication October 11, 2024.
*Correspondence: russell.spencer@theorie.physik.uni-goettingen.de or anna.shnyrova@ehu.eus or mmueller@theorie.physik.uni-goettingen.de
Editor: Padmini Rangamani.
https://doi.org/10.1016/j.bpj.2024.10.009
© 2024 The Author(s). Published by Elsevier Inc. on behalf of Biophysical Society.
This is an open access article under the CC BY license (http://creativecommons.org/licenses/by/4.0/).

Experimental and early theoretical approaches showed that the stress-driven lipid rearrangements can follow a leakless fission pathway characterized by sequential disconnection of the inner and outer monolayers of the neck's membrane (4,6–8). The major free-energy barrier here is associated with the formation of a hemifusion (HF) intermediate, closely resembling a fusion stalk, i.e., an hourglass-shaped monolayer structure, which can expand, resulting in a structure identical to a worm-like micelle (WLM). The free-energy barriers for the stalk or WLM formation are lowered by membrane constriction so that these structures readily form in silico models of membrane fission (8–10). But, so far, these nonbilayer intermediates have eluded direct experimental observation.

Alternatively, membrane stress has been linked to membrane poration as observed experimentally and in coarse-grained (CG) simulations (11–15). Then the question remains, what are the parameters that push the reaction toward leakless fission or another remodeling pathway. In this sense, membrane tension is a major, yet surprisingly overlooked, parameter to explore. It is a crucial constriction cofactor in protein-driven fission (16–20). It is also a major cause of poration, with high tension known to quickly produce pores and rupture of the lipid bilayer (11,14,15,21,22). How these constructive and destructive faces of tension operate in fission remains largely unexplored. In single-membrane systems, lateral tension or similar bulk forces (such as, e.g., friction-induced tension) can produce substantial membrane constriction, critical for protein-driven fission (20). However, it remains unexplored whether tension alone can drive fission via the HF intermediate all the way in a single-membrane system.

Curiously, despite the general consensus on the fission pathway for single-membrane nanotubes (sNTs), there are virtually no such predictions for remodeling of double-membrane systems, e.g., division of mitochondria or formation of annular gap junctions. The coexistence of two concentric membranes during fission-neck constriction complicates the topological outcome of constriction stress, as direct contact between the inner and the outer membranes cannot be ruled out. Indeed, experimental data suggest that close contact between both bilayers during mitochondrial division is possible as 1) close proximity of the inner and outer membranes of mitochondrial fission neck has been documented in ultrastructural studies (23–26) and 2) mitochondrial fission machinery and mitochondrial outer-membrane poration are linked through the programmed cell death process, where both have been implicated (27,28). Interestingly, membrane tension has been proposed to play a key role in mitochondrial fission (19). However, the exact remodeling pathway promoted by high membrane tension during mitochondrial division remains unknown.

One of the reasons behind the lack of theoretical and experimental descriptions of double-membrane neck remodeling is the inherent topological complexity of the process. So far, there have been virtually no double-membrane systems in place to systematically study this phenomenon. The methods available to theory vary significantly in the level of detail used to represent membranes. At one extreme, particle-based simulations represent the system as a set of particles (often atoms), which spontaneously assemble into a membrane (9,29–38). These simulations capture all behaviors exhibited by the membrane, but at an extreme computational cost. Modeling large-scale rearrangements requires huge systems to be simulated for long times and is currently out of reach for these fine-grained simulations.

At the other extreme of membrane modeling, one can ignore the molecular degrees of freedom and consider a membrane itself as an elastic sheet. This gives rise to equation-based free-energy descriptions, such as the Helfrich Hamiltonian, which can be adapted to high curvatures and the effects of lipid tilt and splay in the membrane (39–46). These methods coarse grain the behavior of lipids and proteins into just a few parameters and can easily capture the large-scale behavior of membranes. The ignored molecular details, however, contribute significantly to the free-energy and may become important when modeling topology-altering processes that involve complicated lipid rearrangements. If the membrane itself is the basic structure being considered, as opposed to being emergent, it is challenging to describe nonmembrane intermediate structures, such as WLMs.

There are multiple alternative ways to study the free-energy during membrane rearrangements (47–49). One highly successful method for studying membranes and amphiphilic molecules is self-consistent field theory (SCFT). This method considers the statistics of each molecular species and represents molecular interactions using fields, modeling the potential felt by each molecule at a given point in space. The statistics of molecules in these fields are calculated, giving the average local densities, and the field is adjusted until it accurately represents the particle interaction potentials expected from these densities. In addition to being very successful for traditional polymer systems, SCFT has already proven useful for studying lipid membranes (22,26,50–57). We will employ SCFT to study membrane fission.

Although SCFT has been highly successful, being a mean-field theory, it lacks some details available to particle-based simulations. 1) Particle-based simulations include fluctuations, which may affect the equilibrium behavior, 2) these fluctuations can allow the system to explore configuration space and discover alternative pathways, and 3) the transition pathway from SCFT contains no timescale, whereas particle-based simulations give access to actual

dynamics. For this reason, we use particle-based simulations to validate our SCFT results.

Regardless of the calculation or simulation method, changes in membrane topology during double-membrane fission can involve complicated rearrangements. Tracking the progress of these changes can be challenging, as they are often not amenable to a simple reaction coordinate. The string method offers an elegant solution (34,35) employing a reaction coordinate in terms of changes of the local density as the transformation progresses. A path in density configuration space is then locally optimized, converging to the most likely transition mechanism, the minimum free-energy path (MFEP). The string method has proven useful for obtaining the MFEP in various polymer and membrane systems (22,26,36–38,45,58–60).

In a recent publication, we combined new experimental and theoretical approaches for the ultrastructural characterization of sNTs and double-membrane nanotubes (dNTs) and discovered new pathways for the fission of membrane tubes, catalyzed by transient fusion events (26). This follow-up study expands upon this work, presenting a detailed description of single-membrane fission and expanding the characterization of double-membrane fission to explore a variety of possible fission pathways available to double-membrane tubes. We use SCFT of amphiphilic molecules to model the fission of single and double NTs under tension. Combining SCFT with the string method, we find the MFEPs connecting stable and metastable membrane configurations. Our results are then validated using a specifically developed cryoelectron microscopy (cryo-EM) methodology alongside particle-based simulations. Taken together, our findings provide new mechanistic insights into the process of membrane fission, particularly for double-membrane systems.

MATERIALS AND METHODS

Our experimental and theoretical methods closely follow those described in (26). The methods are described below and further details can be found in the supplemental information of (26).

Experimental methods

Formation of NTs for cryo-EM observations

Upon mixing 99:1 mol % of POPC and 1,2-dioleoyl-*sn*-glycero-3-phosphoethanolamine-*N*-(lissamine rhodamine B silfonyl) (ammonium salt) stocks in chloroform to a final 1 g/L concentration, the chloroform was evaporated under vacuum. The lipid lamellae were then hydrated in 1 mM HEPES (pH 7.0), resulting in the formation of multilamellar vesicles. A 0.4-μL drop of the multilamellar vesicle solution was placed on the edge of the carbon-coated side of a glow-discharged Quantifoil R 2/2300 mesh copper grid, and dried again under vacuum for 30 min. The grid, held with forceps near the lipid reservoir, was placed in a Leica EM GP2 cryoplunger, maintained at 10°C and 90% relative humidity. Next, 3–4 μL of 1 mM HEPES (pH 7.0) was added to the grid from the carbon-coated side. The lipids were allowed to rehydrate for 5 min. After incubation, the liquid was removed by blotting with an absorbent filter paper (Ø55 mm, Grade 595, Hahnemühle) on the grid edge opposite to the lipid reservoir for 3 s. Subsequently, the grid covered with the "ultrathin liquid film" (~100–300 nm thickness) was abruptly plunged into liquid ethane.

Cryo-EM observation of the NT specimens

Once the specimen was frozen, the vitrified grids were maintained in liquid nitrogen and visualized on a JEOL JEM-2200FS/CR, equipped with a field emission gun operated at 200 kV and an in-column Ω energy filter. During imaging, no-tilted zero-loss two-dimensional (2D) images were recorded under low-dose conditions, utilizing the "minimum dose system (MDS)" of Jeol software, with a total dose on the order of 30–40 electrons/$\mathring{A}^2$ per exposure, at defocus values ranging from 1.5 to 4.0 μm. The in-column Omega energy filter of the microscope helped us to record images with improved signal/noise ratio by zero-loss filtering, using an energy selecting slit width of 20 eV centered at the zero-loss peak of the energy spectra. Digital images were recorded in linear mode on a 3840 × 3712 (5 μm pixels) Gatan K2 Summit direct detection camera (Gatan, Pleasanton, CA, USA) using DigitalMicrograph (Gatan, Pleasanton, CA, USA) software, at nominal magnifications of 2000× and 25,000× with pixel sizes of 1.6 and 0.154 nm, respectively. Images were subsequently treated and analyzed using ImageJ software (61).

Theory

In aqueous environments, amphiphilic lipid molecules spontaneously organize into double-layered membranes. We model each lipid molecule as an AB diblock copolymer with fN segments of type A (tail) and $(1-f)N$ segments of type B (head). The surrounding medium, generally water, is represented by a simple homopolymer consisting of N_s segments of type B. We employ a flexible Gaussian chain model for the molecules, assigning each segment a statistical length of b and a volume of ρ^{-1}. The natural end-to-end length of a lipid molecule is represented by $R_0 = b\sqrt{N}$, which serves as the unit of length in our analysis.

A system of volume $V = (n_\ell N + n_s N_s)/\rho$ contains n_ℓ lipids and n_s solvent molecules, each consisting of N and N_s segments, respectively. We set $N_s = N/10$ and $f = 0.8$. Interactions between A and B segments are described by a fixed Flory-Huggins parameter, $\chi N = 30$. Our SCFT calculations are performed in the semigrand canonical ensemble, where we fix the exchange chemical potential, $\mu = \mu_\ell - \mu_s$ allowing n_ℓ and n_s to vary.

SCFT

The structure and thermodynamics of our lipid solution is calculated using SCFT (62,63), the implementation of which follows (26,64). Molecules are modeled as Gaussian chains with the position along the molecule parameterized by t, where $0 < t < 1$ for the lipids and $0 < t < N_s/N$ for the solvent molecules. Molecular statistics are calculated using partial partition functions, or propagators, represented by $q_j(\mathbf{r}, t)$, where $j = \ell$ or s for the lipid or solvent, respectively. The propagator satisfies

$$\frac{\partial}{\partial t} q_j(\mathbf{r}, t) = \left[\frac{R_0^2}{6} - w_\gamma(\mathbf{r})\right] q_j(\mathbf{r}, t) \quad (2)$$

where $w_\gamma(\mathbf{r})$ is a potential field felt by a γ-type segment. For a system with two types of segments, A and B (i.e., $\gamma = A$ or B) it is convenient to write the fields in terms of a segregation field, $w_-(\mathbf{r}) = (w_A(\mathbf{r}) - w_B(\mathbf{r}))/2$ and a pressure field, $w_+(\mathbf{r}) = (w_A(\mathbf{r}) + w_B(\mathbf{r}))/2$. Whereas the fields $w_A(\mathbf{r})$ and $w_B(\mathbf{r})$ are conjugate to the (dimensionless) segment concentrations $\phi_A(\mathbf{r})$ and $\phi_B(\mathbf{r})$, the new fields are conjugate to the local segregation, $\phi_-(\mathbf{r}) = \phi_A(\mathbf{r}) - \phi_B(\mathbf{r})$, and total concentration $\phi_+(\mathbf{r}) = \phi_A(\mathbf{r}) + \phi_B(\mathbf{r})$, respectively.

The lipid tail groups ($t < f$) are labeled A type, and the headgroups ($t > f$) and water are both B type. $q_j(\mathbf{r}, t)$ is calculated by integrating Eq. 2 with uniform initial conditions, as the ends of the molecules are unconstrained.

We also require a "back" propagator, $q_j^{\dagger}(\mathbf{r},t)$, which is similar, but is solved with one side of Eq. 2 multiplied by -1, as we are integrating from the other end of the molecule. The total partition functions for the lipids and solvents are

$$Q_\ell = \int q_\ell(\mathbf{r},1)d\mathbf{r}, \tag{3}$$

$$Q_s = \int q_s(\mathbf{r},N_s/N)d\mathbf{r}. \tag{4}$$

Given a particular set of field configurations, $\{w_\gamma(\mathbf{r})\}$, the product of the forward and back propagators, $q_j(\mathbf{r},t)q_j^{\dagger}(\mathbf{r},t)$, gives the Boltzmann-weighted number of ways to place segment t at spatial position $\mathbf{r}$ and is therefore proportional to the average concentration of segment t. The average dimensionless concentrations of each type of segment are found by scaling this product by the fugacity, $z \equiv \exp(\mu/k_BT)$, and integrating over the relevant parts of the molecule

$$\phi_A(\mathbf{r}) = z\int_0^f q_\ell(\mathbf{r},t)q_\ell^{\dagger}(\mathbf{r},t)dt, \tag{5}$$

$$\phi_{B,\ell}(\mathbf{r}) = z\int_f^1 q_\ell(\mathbf{r},t)q_\ell^{\dagger}(\mathbf{r},t)dt, \tag{6}$$

$$\phi_{B,s}(\mathbf{r}) = \int_0^{N_s/N} q_s(\mathbf{r},s)q_s^{\dagger}(\mathbf{r},t)dt, \tag{7}$$

The total B concentration is thus $\phi_B(\mathbf{r}) = \phi_{B,\ell}(\mathbf{r}) + \phi_{B,s}(\mathbf{r})$, and the total lipid concentration is $\phi_\ell(\mathbf{r}) = \phi_A(\mathbf{r}) + \phi_{B,\ell}(\mathbf{r})$. In the usual manner of SCFT, we calculate the following local exchange chemical potentials, which act on the segregation and total concentration, respectively,

$$\mu_-(\mathbf{r}) = \chi N\phi_-(\mathbf{r}) - 2w_-(\mathbf{r}),$$

$$\mu_+(\mathbf{r}) = \chi N(\phi_+(\mathbf{r}) - 1), \tag{8}$$

These local exchange chemical potentials drive the system to change configuration, thus stable and metastable configurations occur when they vanish. SCFT is done by initializing the system with some initial fields, $w_-(\mathbf{r})$ and $w_+(\mathbf{r})$, and relaxing the system toward $\mu_-(\mathbf{r}) = \mu_+(\mathbf{r}) = 0$ by updating the fields by an amount proportional to the exchange chemical potentials. We are typically satisfied with a root mean-squared error of $\lesssim 10^{-4}$.

The grand canonical free-energy, F, is then calculated using

$$\frac{F}{\sqrt{\overline{\mathcal{N}}}k_BT} = -Q_s - zQ_\ell + \int\left(\frac{w_-^2}{\chi N} - w_+(\mathbf{r})\right)d\mathbf{r} \tag{9}$$

where $\overline{\mathcal{N}} = (\rho R_0^3/N)^2$ is the invariant polymerization index. Once we have obtained stable or metastable structures, we compare the free energies to determine the relative stability of different phases. The quantity $\overline{\mathcal{N}}$ depends on specific system parameters, such as the molecular weight of the lipids and the density of statistical segments. In practice, we treat the energy scale, $\sqrt{\overline{\mathcal{N}}}k_BT$, as a fitting parameter and match to experiments by comparing known quantities. A convenient choice is the membrane bending modulus, κ.

We typically solve Eq. 2 using a pseudospectral method (65) with 60 steps along the lipids and 6 along the homopolymers (solvent). We typically use a 3D grid of dimensions $16R_0 \times 12R_0 \times 12R_0$ and discretization of $0.08R_0$ to represent the concentrations and fields. For the cylindrically symmetric phases, however, we can greatly decrease computation time by using a 2D grid and a cylindrical coordinate system.

String method

To study transitions between stable and metastable structures, we use the string method (34,35,66–70). First, we must describe the system in configuration space: each dimension of this space corresponds to the state of the system (given by $w_-(\mathbf{r})$) at the point $\mathbf{r}$. The state of the system is given by a point (or vector) in this high-dimensional space, and its evolution is described by motion through the space. The set of local exchange chemical potentials at all points, i.e., the function $\mu_-(\mathbf{r})$, corresponds to a vector in this space and drives this transformation along its evolution path. The string method seeks a path that is tangent to $\mu_-(\mathbf{r})$, as this corresponds to a (thermodynamically reversible estimate for) the most probable pathway between metastable states given by the MFEP connecting configurations (34,35).

A path between two states is discretized along the reaction coordinate into m configurations indexed by i in the range $1 \le i \le m$. Each point in configuration space is described by its segregation field $w_-^{(i)}(\mathbf{r})$, with incompressibility enforced ($w_+^{(i)}(\mathbf{r})$ equilibrated) independently. This allows us to define a reaction coordinate, α, determined by calculating the Euclidean distance between adjacent points on the string and normalizing the total string length to 1.

The ends of the string $i = 1$ and $i = m$ are updated as described above; however, we only update the intermediate positions *perpendicularly* to the path of the string to find a path with tangent vector $\mu_-(\mathbf{r})$. This involves defining a vector parallel to the string, $\tau(\mathbf{r},\alpha) \equiv \frac{dw_-(\mathbf{r},\alpha)}{d\alpha}$. We find $\tau(\mathbf{r},\alpha)$ by fitting $w_-(\mathbf{r},\alpha)$ pointwise along the α direction using a cubic spline. We then calculate the portion of $\mu_-(\mathbf{r})$ parallel to $\tau(\mathbf{r},\alpha)$,

$$\mu_-^{\parallel}(\mathbf{r},\alpha) = \hat{\tau}(\mathbf{r},\alpha)\left(\int \mu_-(\mathbf{r},\alpha)\hat{\tau}(\mathbf{r},\alpha)d\mathbf{r}\right) \tag{10}$$

where $\hat{\tau} = \frac{\tau}{\|\tau\|}$ with $\|\tau\|(\mathbf{r},\alpha) = \frac{1}{V}\sqrt{\int \tau(\mathbf{r},\alpha)^2 d\mathbf{r}}$. Notice that the spatial integral is a scalar product in configuration space. We then subtract $\mu_-^{\parallel}(\mathbf{r},\alpha)$ from $\mu_-(\mathbf{r})$, resulting in the perpendicular component,

$$\mu_-^{\perp}(\mathbf{r},\alpha) = \mu_-(\mathbf{r},\alpha) - \mu_-^{\parallel}(\mathbf{r},\alpha). \tag{11}$$

In addition to the perpendicular updates, we restrain the points to be equidistant in configuration space by shifting them along the path using the aforementioned fit. The update procedure can in fact be done using the entire local exchange chemical potential (not simply the perpendicular component) followed by redistributing the points; however, the perpendicular update provides a more direct update and a far better estimate of the degree of convergence. The completely converged string satisfies $\mu_-^{\perp}(\mathbf{r},\alpha) = 0$; however, due to the computationally intensive nature of these calculations, we are typically satisfied with a root mean-squared error of $\lesssim 10^{-3}$.

The MFEP connecting one (meta)stable state (local free-energy minimum) to another passes through a saddle-point in the free-energy, where $\frac{d^2F}{d\alpha^2} < 0$. The MFEP from the saddle-point to a metastable state corresponds to a simple "downward" motion, i.e., $\frac{dw_-}{d\alpha} \propto \mu_- \propto \frac{\delta F}{\delta w_-}$. The first proportionality is true when μ_- is parallel to the path, i.e., when $\mu_-^{\perp}(\mathbf{r},\alpha) = 0$. The MFEP connecting two metastable states may be direct or include other intermediate metastable states, and thus multiple saddle-points (barriers).

Particle simulations

In addition to the mean-field calculations conducted with SCFT and the string method, we also employ particle-based simulations using the versatile and well-tested soft coarse-grained Monte-Carlo acceleration (SOMA) simulation package (71). This allows us to compare our SCFT predictions with particle-based simulation results, which include

fluctuations and do not assume that molecular configurations relax quickly as compared with densities. Simulations also allow us to extract a timescale, which is absent in SCFT calculations. The particle-based simulations are conducted using $\sqrt{\mathcal{N}} = 100$. This is in the experimentally relevant regime, as judged by comparing our bending energy, $\kappa = 0.209\sqrt{\mathcal{N}}k_BT$, with the experimental value of $\kappa \approx 39k_BT$ (72). We use a Helfand compressibility energy

$$\frac{U_\kappa}{k_BT} = \frac{\rho\xi N}{2}\int d\mathbf{r}(\phi_A(\mathbf{r}) + \phi_B(\mathbf{r}) - 1)^2 \quad (12)$$

where we choose $\xi N = 100$. This large value of ξN is used to approximate the incompressible system considered in SCFT.

RESULTS

We start with the simple case of sNT fission, comparing theoretical and experimental results. Then we investigate how a second NT complicates the topological transition in dNT fission, as dNTs offer many more degrees of freedom. As a result, we explore many possible rearrangements as steps in an overall pathway and then compare the free-energy barriers of each step to conclude the most likely pathway to fission. The preferred pathway is finally presented as a function of membrane tension.

We use the membrane tension as a control parameter in the theoretical calculations, as it is a simple way to implement constriction in tubular membranes. The tension and free-energy are given in units of the bending energy of a bilayer, $\kappa = 0.209k_BT\sqrt{\mathcal{N}}$, and bilayer thickness, $d = 1.22R_0$, where $\mathcal{N}$ measures the density of lipid molecules, and R_0 is the average end-to-end length of the lipid molecule. The bending energy and bilayer thickness depend on the lipid but, for ease of comparison, a purely POPC membrane used in the experiments throughout this work is characterized by $\kappa \approx 39\ k_BT \approx 1.6 \times 10^{-29}$ J (at $T = 300$ K) (4,54) and has a thickness of $d = 4$ nm (72). The unit conversion factor is thus simply $\kappa/d^2 \approx 9.86$ mN/m (see supporting material).

Detailed results are shown for tensions of $\sigma = 0.129\kappa/d^2 \approx 1.27$ mN/m and $\sigma = 0.387\kappa/d^2 \approx 3.82$ mN/m. These are on the high end of physiological tensions, 10^{-3} – 3 mN/m (73,74); however so is, presumably, the membrane tension at the constriction site during a fission event. All pathways that we have found available at low tension are also available at higher tension, but the reverse is not true. We therefore use key images during paths at $\sigma = 0.387\kappa/d^2 \approx 3.82$ mN/m as exemplars, highlighting where reducing the tension leads to differences. Summary data show a full range of tensions, down to almost zero.

Single-membrane tube fission

Our first step is to reconstruct the remodeling pathway of NTs under tension with experimental data and theoretical predictions. The free-energy along the MFEP, $0 \leq \alpha \leq 1$, of sNT fission is presented in Fig. 1 *A*. Our calculations indicate that, before fission occurs, the sNT constricts locally, forming an HF intermediate, where the free-energy peaks. The HF intermediate then expands into a metastable WLM, connecting two capped NTs. The WLM subsequently constricts and disconnects close to one of the caps, where the free-energy peaks again. Finally, the WLM retracts into one of the capped NTs, which recedes due to membrane tension.

Using α as the reaction coordinate captures the cumulative change in the spatial distribution of lipid tails throughout the transformation, but it is not easy to interpret as its correspondence to intuitive descriptions of the system changes along the transformation process. During constriction, α reflects changes in tube radius, then the length of the WLM as it grows. During WLM rupture, α reflects its thickness at the rupture site. Finally, α reflects the distance between capped tubes as they retract. The ability of the MFEP to identify a suitable reaction coordinate, α, makes it ideal to simulate complicated transformations but, when it comes to interpretation, it is useful to calculate coordinates reflecting changes that occur at specific parts of the transformations.

One useful reaction coordinate is the local tube radius, $R = \int r\phi_\ell(\mathbf{r})dA / \int \phi_\ell(\mathbf{r})dA$, where r is the distance from the tube axis. R is calculated by evaluating the integral over a plane normal to the tube axis and passing through the constriction site. For an equilibrium tube, this gives the tube radius, R_c, and for a WLM it is R_{WLM}. During fission, we find the minimum value of R at the constriction site. In the case of tube constriction this is the radius of the constricted tube, whereas for fissioning WLM, it measures the thickness of the WLM at the fission site. The free-energy of the unperturbed tube is, of course, a local minimum; thus, as seen in Fig. 1 *B*, the free-energy at small $R_c - R$ depends quadratically thereon. The quadratic behavior quickly gives way to an approximately linear dependence, with a slope (constriction force) that does not significantly depend on tension. This suggests that the main source of the barrier comes from the degree to which the tube must be constricted, i.e., the equilibrium tube radius, and also that a constant force is required to constrict the tube, independent of radius. It is important to note that this is a local constriction and is markedly different from a global constriction brought about by, for example, tension in the membrane.

To confirm our theoretical predictions experimentally, we developed a method that allows the formation and ultrastructural observation of lipid NT geometry by cryo-EM. Moreover, as the transitional states are highly unstable, and thus difficult to detect in stationary experimental conditions, we cryopreserved NTs right upon inducing NT fission by hydrodynamic flow. Such quasidynamic conditions rendered snapshots of the main stages of the NT fission process (Fig. 1 *C*) that further confirm our predictions. We find that extreme constriction of the membrane neck (Fig. 2 *C*,

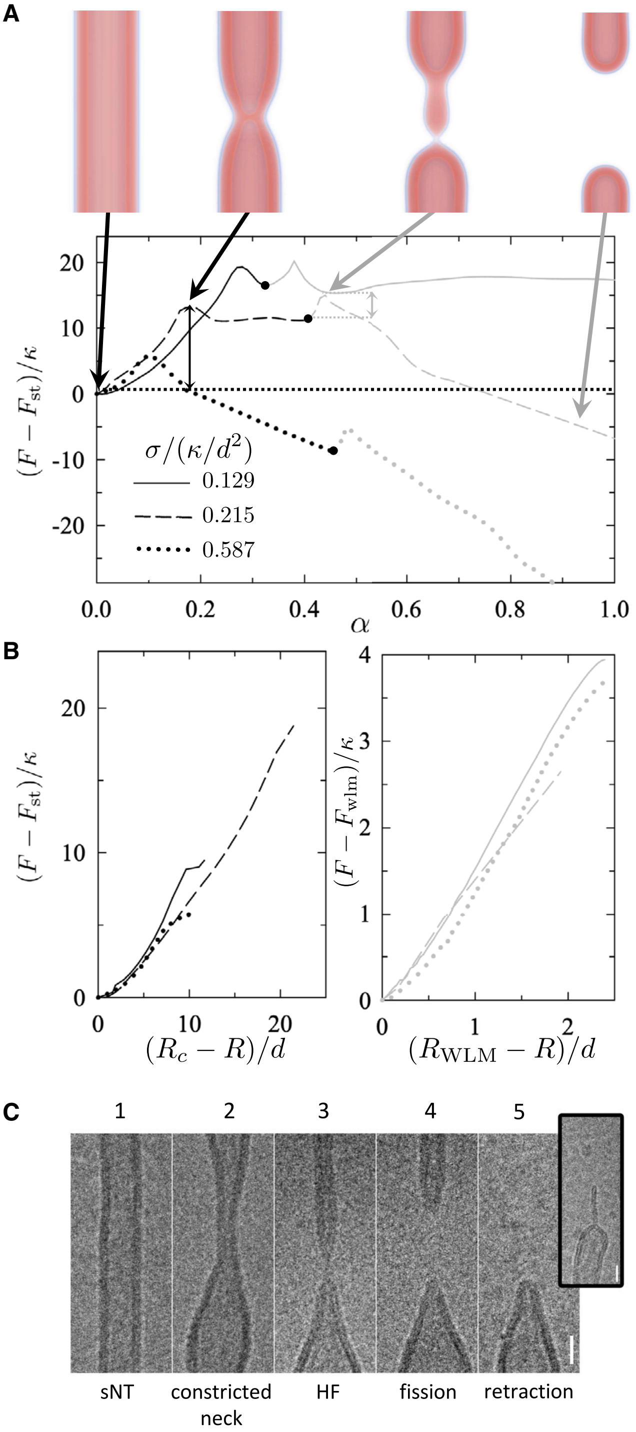

FIGURE 1 Pathway of sNT remodeling during scission. (*A*) Free-energy (relative to that of a sNT, F_{st}) along the MFEP during sNT fission. Graphs are shown for (*solid*) $\sigma = 0.129\kappa/d^2$ (1.3 mN/m for a POPC membrane), (*dashed*) $0.215\kappa/d^2$ (2.1 mN/m), and (*dotted*) $0.587\kappa/d^2$ (5.8 mN/m). Images depict lipid densities at characteristic points along the pathway. A sNT divides into two capped tubes, moving apart and exiting the system. The final state still contains two capped tubes. Transition barriers are indicated by vertical arrows for the middle (*solid*) curve. (*B*) Fission is divided into two steps (*left*) constriction and (*right*) WLM fission, and free energies are shown as functions of calculated order parameters, discussed in the text. The order parameters are only calculated (*left*) when the tube is constricting and (*right*) when the WLM is thinning, i.e., in the increases leading to the maxima. (*C*) Cryo-EM snapshots of main sNTs remodeling steps during fission upon increase of membrane tension (> 1 mN/m). Scale bars, 20 nm. Step 1 corresponds to the sNTs. Constriction of the NT is seen in step 2. At step 3, the NT partially collapses forming an HF/WLM intermediate. The inset shows an elongated WLM sometimes forming at this step. Membranes disconnect at step 4 and retraction to the reservoir is observed in step 5.

step 2) (4) is followed by its collapse into a stalk or WLM structure (Fig. 1 *C*, *step 3*). We also detected the predicted membrane retraction upon rupture of the HF intermediate. (Fig. 1 *C*, *steps 4* and *5*).

Interestingly, the probability of the formation of the WLM structure depends on the initial membrane tension. At high membrane tension (*dotted curve* in Fig. 1 *A*), the extension of the WLM becomes favorable, as its free-energy, F_{WLM}, is smaller than that of the sNT, F_{sNT} (see the supporting material for further discussion). Indeed, in our dynamic experimental conditions, where membranes are subjected to high tension, we could observe extended WLMs even after fission occurred (see Fig. 1 *C*, supporting material, and (26)). For intermediate tension, close to that of the constricted neck (*dashed curve* in Fig. 1 *A*), $F_{WLM} \approx F_{sNT}$. At low tension (*solid curve* in Fig. 1 *A*), the WLM becomes unfavorable as $F_{WLM} > F_{sNT}$. In this case, the WLM breaks without extending significantly.

In our calculations done at high tension, where $F_{WLM} < F_{sNT}$, the system can lower its free-energy by elongating the collapsed region. This is apparent from the negative slope seen in the dotted curve of Fig. 1 *A* as the WLM grows, and continues to be true for arbitrarily extended WLMs. It therefore does not reach a true minimum before fission, but rather WLM fission constitutes a jump over a barrier in a direction nonparallel (in configuration space) to WLM elongation. In practice, this suggests that, at high tension, the gradual conversion of tube into WLM will be randomly interrupted by the fission of the WLM at one of its connection sites.

Of note, high tension in membranes is usually associated with pore formation. Therefore, we address this possibility. Fig. 2 shows that the barrier to pore formation in single membranes is much larger than the free-energy of the leakless fission pathway presented above. However, the poration barrier in Fig. 2 may be overestimated, as we approximated it using the classical nucleation theory in a planar membrane. We decided to corroborate this result by 1) manually inserting pores into the sNT fission sequences near sites of high membrane curvature (e.g., the WLM-membrane fusion site) and 2) creating a fission pathway that starts from a nucleating pore, which "unzips" around the membrane, leading to fission. In both cases, as the string relaxed (i.e., the pathway evolved from our artificial initial state to an optimal mechanism), the pores closed and the sequence converged to the fission mechanism described above.

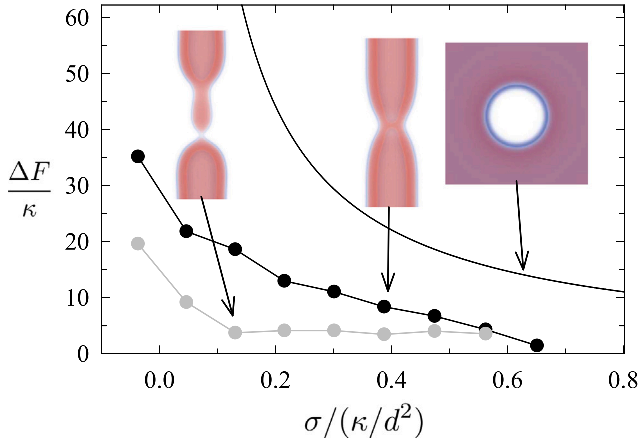

FIGURE 2 Free-energy barriers are shown for the (*black points*) first and (*gray points*) second barriers to sNT scission (see Fig. 1). Also shown is (*curve*) the barrier to pore formation ($\Delta F = \pi\gamma^2/\sigma$, where γ is the line tension of a bilayer edge) calculated from classical nucleation theory.

Hence, our results suggest that, in sNTs, fission would preferentially proceed without pores even under relatively high membrane tension. See supporting material for further details.

While formation of a pore is a costly process and not an optimal or expected pathway, in real biological systems, one cannot completely exclude membrane poration as part of the fission mechanism. If an external agent, e.g., a protein, stabilizes a pore at the initial configuration, fission may still progress through the leaky unzipping pathway (see supporting material for further details). This does not preclude the possibility of fission via poration from occurring, but it is not an optimal path.

Double-membrane tube fission

While single-membrane systems undergo fission via the HF intermediate, the first systematic studies about the remodeling paths of double-membrane systems occurred only recently (26). Given the evidence provided therein for the role of intermembrane fusion in membrane fission, and the above success in describing the fission of sNTs, we turned to in silico calculations to find the possible pathways for dNTs remodeling in different membrane-tension regimes.

Sequential fission pathway

The first case we consider is the one of sequential fission of first the inner and then the outer bilayer of a double-membrane system (Fig. 3). The inner NT proceeds to HF, which extends into a WLM and then ruptures and retracts. Both retracting capped NTs remain inside the outer NT until it undergoes fission in a similar way. Free-energy calculations, similar to those presented above, show that the barrier to inner NT rupture is not significantly affected by the presence of the outer NT (the difference is small on the scale of free energies presented) (26). Importantly, this pathway is leakless, as in the case of sNTs discussed above.

Pathways to intermembrane fusion

Next we explore the landscape of possibilities that include intermembrane contact. Evidence of intermembrane contact has been presented in (26) and is recapitulated in Fig. 4.

In the simplest case, the inner and outer NTs would fuse—direct-HF pathway. This is illustrated in Fig. 5, *A* and *B* (see also Videos S2 and S3). The first step here is the formation of a metastable stalk—i.e., a hydrophobic bridge—between both membranes. At this point we find the first bifurcation in the pathway. The stalk can simply widen (i.e., radially expand) and flatten (Fig. 5 *A*), forming a metastable hemifusion diaphragm (HD), as has been seen previously (50). Alternatively, the presence of the stalk can lead to a thinning of the surrounding membrane, which then heterogeneously nucleates a pore in the outer (Fig. 5 *B*) or inner membrane (see supporting material). The connection initiated by the stalk then expands around the pore, as in

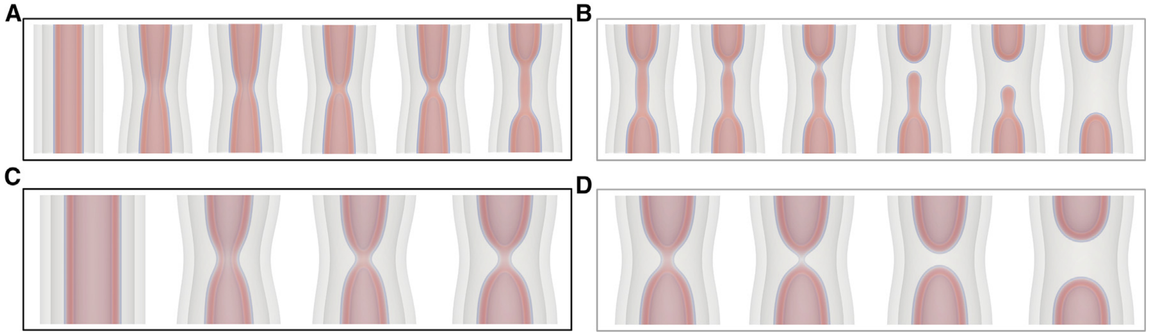

FIGURE 3 Rupture of the inner tube directly, by way of a WLM intermediate at (*top: A* and *B*) $\sigma = 0.387\kappa/d^2$ and (*bottom: C* and *D*) $\sigma = 0.129\kappa/d^2$. This pathway is functionally the same as the sNT pathway presented earlier: (*left: A* and *C*) the inner NT locally constricts, forms a WLM that elongates only at high tension, i.e., (*A*); then (*right: B* and *D*) the WLM thins and ruptures, resulting in two capped tubes. For the elongated WLM in (*A*), WLM rupture occurs at one of the connection sites (*B*), followed by WLM retraction, whereas the short stalk-like WLM in (*C*) simply ruptures in the middle (*D*). See also Video S1. The outer tube has been made transparent.

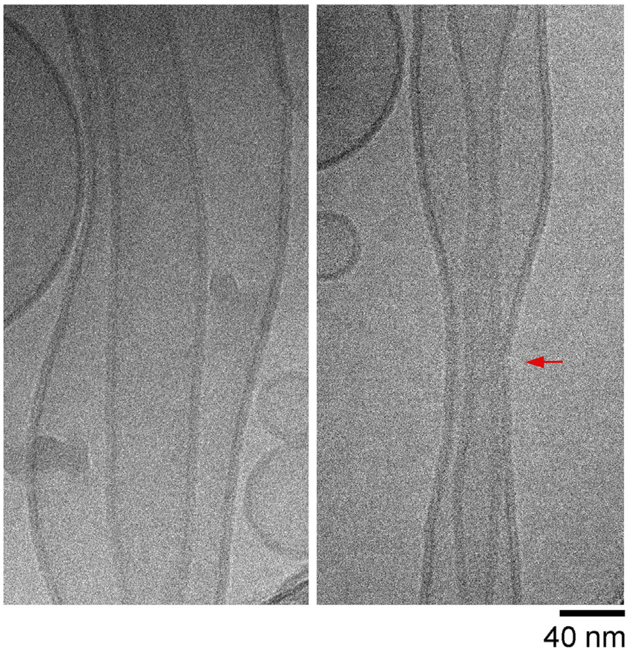

FIGURE 4 Cryo-EM snapshots of dNT constriction at low (*left*) and (*right*) higher membrane tension. The red arrow points to close proximity (<2 nm) of the inner and outer membrane of the dNT upon tension-induced constriction. Reproduced from (26) under the terms of the Creative Commons Attribution License.

fusion studies (75,76). In either case, the final topology is the same: an HD connecting the inner and outer membranes on one side of the dNT.

As illustrated in Fig. 5, *A* and *B*, showing the direct-HF pathway, the steps presented in this section can often occur with small variations, such as the transient pores mentioned above. Rather than trying to discuss each possible variation, we combine sets of potential steps, which are very similar in terms of the actual rearrangement as well as their free-energy barriers (differences typically $< 0.02\kappa$). We consider, for example, the above to be a direct HF, which may or may not include transient pores. Potential variations will be mentioned where relevant and are discussed further in the supporting material. For stalk formation, α reflects the number of lipids in the stalk, as it tracks changes in local lipid number.

Intriguingly, there is an alternative pathway to HF, via the collapsed state—the WLM pathway. We first presented this result in (26) but expand upon it here. Variations of this pathway are illustrated in Fig. 5, *C* and *D*, respectively, and in Videos S3 and S4. The simpler of these, shown in Fig. 5 *C*, is the reverse of the fission-by-fusion pathway presented in (26), calculated here at higher tension. It proceeds as follows: 1) the WLM bends toward the outer membrane, 2) a stalk forms, connecting the inner and outer membranes, 3) the stalk destabilizes the inner membrane and a pore forms, 4) the other capped NT forms a pore resulting in a dNT with a pore on the inner membrane and a stalk connecting the membranes, and 5) the pore then closes, resulting in a dNT connected by a stalk. This final structure is topologically equivalent to the hemifused states at the end of the other processes. The stalk may then widen to form an HD, similarly to the process in Fig. 5 *A*. Although framed as fusion pathways, the paths produced by the string method are reversible, and they may equally be seen as pathways from the hemifused to the collapsed state.

At high tension, the stable WLM intermediate allows for a more complicated path (illustrated in Fig. 5 *D*): 1) the inner NT first collapses into a WLM as in the sNT system, and the outer membrane curves inward, closer to the equilibrium radius of a sNT, 2) this inward bend facilitates the formation of a stalk, connecting the outer membrane and the WLM, 3) a second stalk forms, closer to one of the capped NTs, 4) the stalks move along the WLM toward this capped NT, and a pore forms in the cap, 5) the WLM flattens, and the stalks broaden into diaphragms, 6) subsequently, these diaphragms expand around the pore and form an enclosed connection between one of the inner capped NTs and the outer membrane, and 7) finally, the connected portion expands toward the other capped NT, which fuses with the connected region, reconnecting the two inner NTs. The HD adjusts slightly, forming the same final metastable state as seen in Fig. 5, *A* and *B*. This complicated, multistep pathway is not amenable to a simple-order parameter.

The formation of a second stalk in this second pathway may seem extraneous, as a single stalk could expand around the pore, leading to a more direct path. We manually modified the transformation pathways to check whether the second stalk was an artifact of our initialization. If one of the stalks is removed from the string, it simply reforms when the string is allowed to relax. We, therefore, conclude that the presence of a second stalk is an intrinsic part of this transformation path. This is unlike, for example, the pore in the previous direct-HF pathway, which may or may not form. Once the inner and outer membranes have locally fused by any of the pathways illustrated in Fig. 5, the next step is to expand the HD.

Pathway selection

Fig. 5 presents a number of paths by which a dNT system may form or break connections between the inner and outer membranes. To understand how the system will actually proceed, we need to understand the barriers to each of these processes. The free energies along the path are presented in Fig. 6. Each step in the transition can be thought of as a jump between metastable states (local minima on the free-energy landscape) with a barrier given by the difference between the free-energy at the starting metastable state and the highest free-energy before the next metastable state.

It is important to note that the pathways are reversible, and can proceed in the direction of increasing or decreasing α. In fact, the system can proceed along any of the given

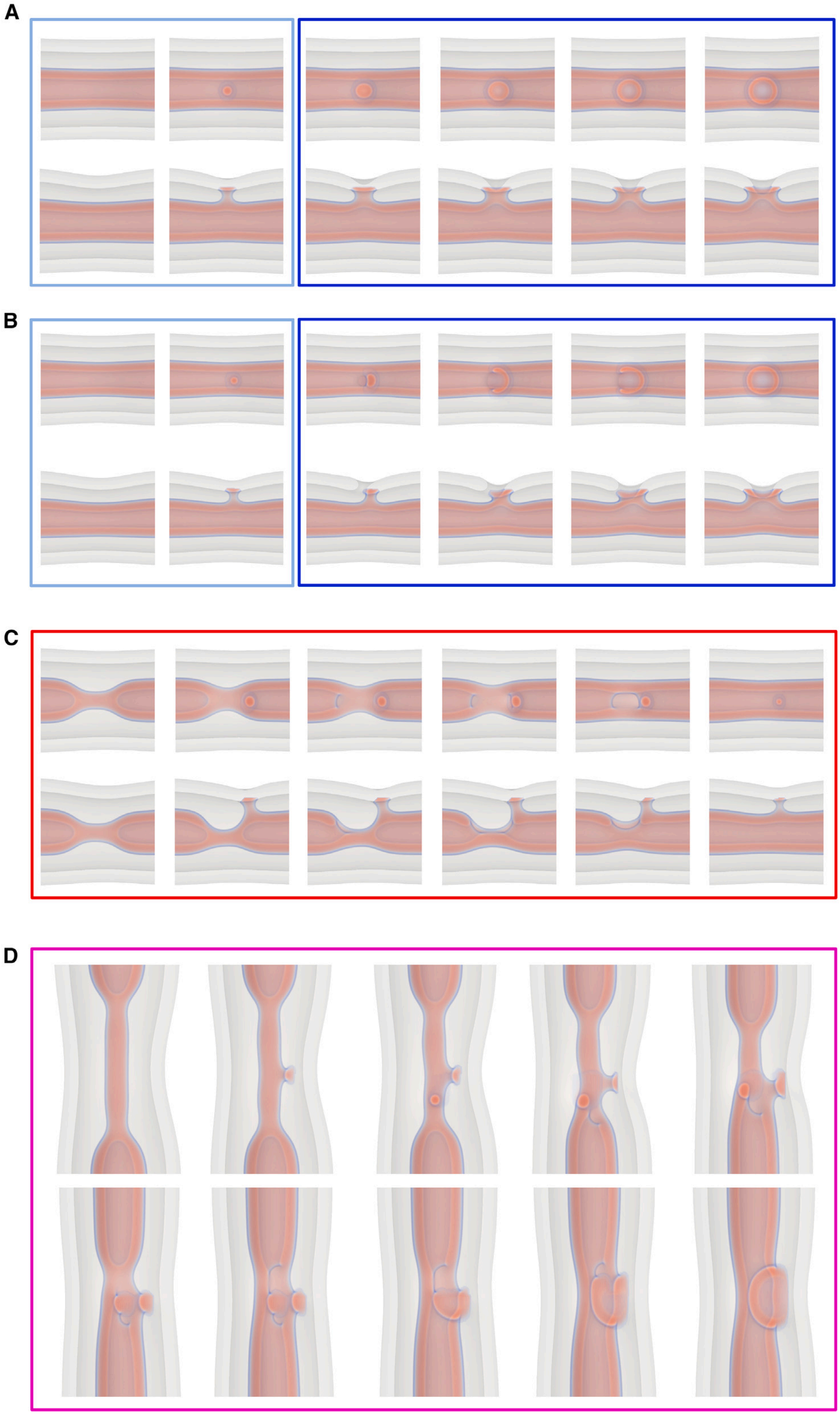

FIGURE 5 Intermediate configurations for the fusion of an inner and outer membrane at a tension of $\sigma = 0.387\kappa/d^2$. Four mechanisms are shown: (*A*) direct hemifusion via a stalk connecting the membranes, (*B*) fusion via a pore in the outer membrane, (*C*) hemifusion via a WLM intermediate, and (*D*) a more complicated pathway to the hemifused state from the WLM intermediate as first described in (26). (*A*–*C*) Show the same configurations from two perpendicular views. The WLM at the start of (*C*) and (*D*) forms according the same mechanism shown previously for a sNT and in Fig. 3. In each case, the outer tube has been made transparent, to emphasize rearrangements of the inner tube.

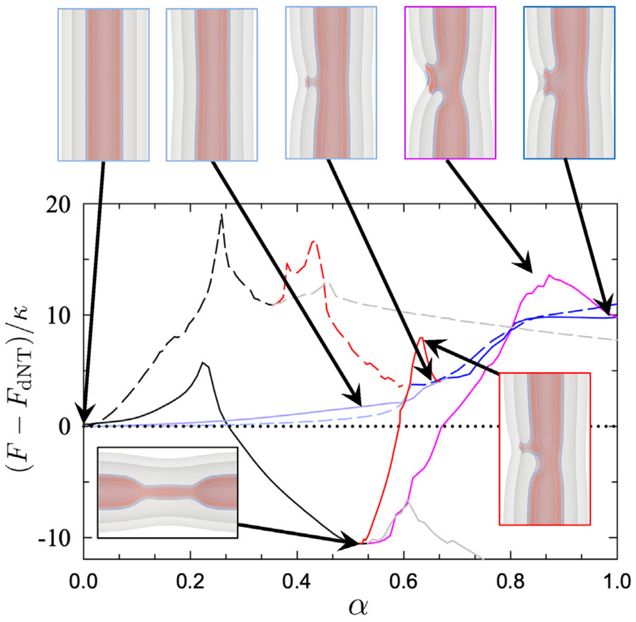

FIGURE 6 Free-energy along the MFEPs for the pathways linking the fused and unfused dNT states shown in Fig. 5. Data are shown for (*dashed*) $\sigma = 0.129\kappa/d^2$ and (*solid*) $\sigma = 0.387\kappa/d^2$, and free energies are given relative to that of a dNT, F_{dt}. Colors correspond to different steps, and are chosen to match those in Figs. 3 and 5. Membrane configurations are shown at key steps for $\sigma = 0.387\kappa/d^2$. Barriers are calculated similarly to Fig. 1.

paths in either direction, with metastable states serving as junction points connecting between paths. As before, local minima correspond to metastable states and the barrier can be inferred by the free-energy peak between these minima. We present data for a tension on the high end of physiological relevance, $\sigma = 0.129\kappa/d^2 \approx 0.2 k_B T/\mathrm{nm}^2$, and a higher tension, $\sigma = 0.387\kappa/d^2$, to illustrate a change in behavior occurring between these values.

Starting from the unperturbed dNT state, there are two possibilities: collapsing the inner tube, as in the sNT case (*black*) or forming a stalk between the membranes (*light blue*). At both tensions, the barrier to stalk formation is $\sim 5\kappa$. At the higher tension, $\sigma = 0.387\kappa/d^2$, the barrier to collapse (*black*) is also $\sim 5\kappa$. As the stalk is only weakly metastable (the barrier to its dissolution is small on the scale of the free energies presented) and thus likely to disconnect before proceeding further, we expect inner tube collapse to be the dominant pathway. At the lower tension, however, the barrier to direct tube collapse is far greater ($\sim 20\kappa$) and less likely to occur.

Once the stalk forms, the system can proceed in a number of ways: the stalk could expand (*dark blue*) into an HD; it could catalyze further fusion of the membranes (*red*, in the decreasing α direction, i.e., Fig. 5 *C*); or the stalk could simply dissolve. If the stalk dissolves, the system simply returns to where it started until it "tries" again, thus we ignore this possibility. The barrier to forming an HD is smaller than the alternatives; however, the HD is only weakly metastable. The free energies presented imply that the HD is unstable, as it corresponds to a plateau in the free-energy ($\sigma = 0.387\kappa/d^2$) or, worse yet, a slope leading back to the stalk state ($\sigma = 0.387\kappa/d^2$). Similar simulations conducted in the canonical ensemble (fixing lipid number, as opposed to tension) produce a free-energy minimum, i.e., stable HD. Based on these calculations, as well as particle simulations showing long-lived stalks and HDs (discussed later), we expect that the slow lipid diffusion will lead to a metastable HD. Due to the weak metastability (or instability) of the HD, and (as we will see) the prohibitive barrier to proceeding further along the HD pathway, we expect it to simply shrink back into the stalk state.

The free-energy barriers to these competing processes are shown in Fig. 7. Starting from the stalk, the barrier to collapse into the WLM state is consistently below the barrier to the canonical pathway, showing that the stalk aids the collapse. The total free-energy cost of both jumps is, however, similar between the canonical pathway and stalk intermediate. Nonetheless, the existence of a stalk intermediate serves as a more easily formed jumping off point, to catalyze the collapse. Once in the collapsed state (second image of Fig. 6, *top*), the WLM easily ruptures (see sNT data) leading to the fission of the inner tube. This is the case for $\sigma \lesssim 0.4\kappa/d^2 \approx 4$ mN/m, i.e., for essentially the entire range of physiologically relevant tensions.

Expanding the HD

Although inner-tube fission is the most likely possibility, other possibilities remain, which may be of physiological relevance. As illustrated in Fig. 8, the HD may expand ("zip") around the NT, resulting in a sNT connecting two dNTs—the cylindrically hemifused (CH) state. During this process, a pore opens in the outer membrane. The pore forms during the final stages of the "zipping" process,

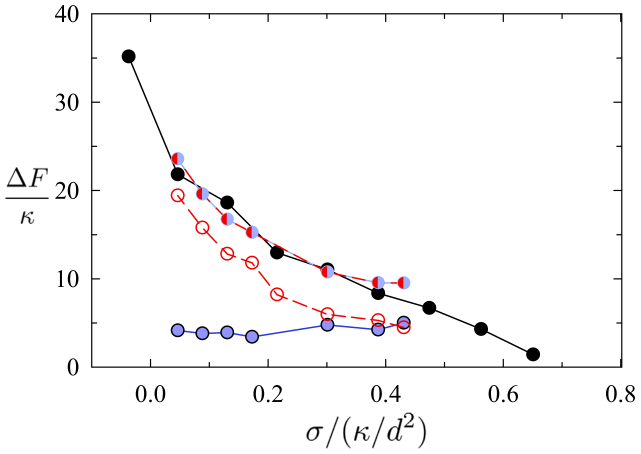

FIGURE 7 Free-energy barriers are shown for the (*black points*) canonical pathway, (*blue*) stalk formation, (*red*) transition from the stalk to the collapsed state, and (*blue* and *red*) sum of the stalk and collapse barriers, i.e., total barrier to collapse via the stalk pathway.

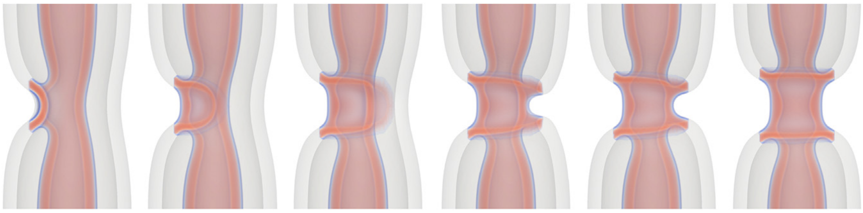

FIGURE 8 Configurations during the "zipping" of the HD toward the CH state at a tension of $\sigma = 0.387\kappa/d^2$. The initial connection between the inner and outer membranes zips along the membranes until the remaining portion is small. A pore then opens (fourth image) and the remaining outer membrane breaks and then fuses with the inner membrane. The outer tube in the double-tube region has been made transparent.

when and where the membrane is strongly curved and would need to curve even further to continuously deform into the final state. Consequently, the bending energy of the highly curved membrane outweighs the line tension of the pore. A transient pore can also form at the leading edge of the zip due to the high curvature. Each of the mechanisms presented here are further discussed in the supporting material, including images that are enlarged and annotated for clarity and a discussion of possible variations of transition pathways.

The free-energy curves during zipping are illustrated in Fig. 9, and the barriers are quite large, for both of the tensions. Without intervention, the barriers to forming the CH state appear to be prohibitively large, and the previously presented pathways are likely to dominate. Potential interventions will be discussed later.

Although a simple-order parameter for the entire process is not forthcoming, we examine the main part of the pathway in terms of the angle around which the HD has zipped, θ_{CH} (Fig. 9 *B*). The free-energy increases roughly linearly with θ_{CH}. The process can be seen as the free-energy change associated with replacing the double membrane with a single membrane plus two threefold membrane junctions. The areas of the membrane replaced and the length of the junctions both vary linearly with θ_{CH}.

Collapse of the CH state

The sNT is metastable and, once this CH state has formed, the evolution may proceed in a number of ways. The most likely pathways involve either disconnecting the inner and outer membranes or simply rupturing the sNT. This section discusses these mechanisms, which are illustrated in Fig. 10. Fig. 10 *A* shows a mechanism where a pore forms in the inner membrane, close to its connection to the diaphragm. The pore then expands, "unzipping" the inner NT from the outer one, but leaving a stalk connecting the inner and outer membranes. This is followed by the closure of the inner NT, creating a capped NT connected to the outer membrane by a stalk. The inner region is now disconnected from the region between the membranes. The stalk then disconnects, leaving a capped NT inside of the outer membrane. As the membranes are under tension, the capped NT then retracts. A possible variation on this pathway involves the formation of two pores, leading to two stalks. Another variation involves the stalks remaining intact, as the inner tube retracts, but remains connected to the outer tube.

Alternatively, as presented in Fig. 10 *B*, the inner NT may first collapse, creating a WLM, connecting it to a diaphragm that spans the outer NT. The WLM then breaks, followed by the diaphragm. The result is, once again, a capped NT inside the outer NT. As the sNT has two sides, the disconnection process must occur twice to disconnect both inner membranes from the outer membrane and produce a pair of capped NTs. The sides may disconnect separately or concurrently. When calculating the free energies, we consider the case of only one side disconnecting. The barrier to the second disconnection is smaller (not shown).

The third option involves the formation of a pore in the sNT connection (Fig. 10 *C*). The pore then grows, unzipping the sNT portion, until the two structures are only connected by a WLM, which subsequently breaks. This resembles fission by poration of a sNT, which was discussed previously and found to be unfavorable. In this case, however, the newly created interface is not simply a membrane edge, and the pore resembles an HD rim pore, which can be more stable than simple membrane pores (64). Unlike cases in Fig. 10 *A* and *B*, the final result is leaky: the region inside the inner membrane is open to the outer fluid. As the membrane is under tension, the two NTs are then pulled away from one another. In our case, disappearing through the reflecting boundary, but in real system they would simply retract.

Once again, we can compare the likelihood of these pathways by examining the free-energy change throughout the process, shown in Fig. 11. At both tensions, the barriers to disconnecting the inner tube (Fig. 10, *A* and *B*) are similar via either process, and smaller than the barrier to rupturing the sNT (Fig. 10 *C*).

The inner tube disconnection processes are not easily amenable to a simple order-parameter. Rupturing the sNT can be measured in terms of the angle through which the pore expands, θ_{st}. The free-energy is roughly linear in terms of θ_{st}. The reasoning is similar to the zipping process: disappearance of the membrane under tension and conversion of the threefold junction into a curved membrane. The threefold junction has a higher line tension than the curved membrane, and deletion of the membrane under tension is favorable, thus the free-energy decreases. The barrier to this process comes from pore formation.

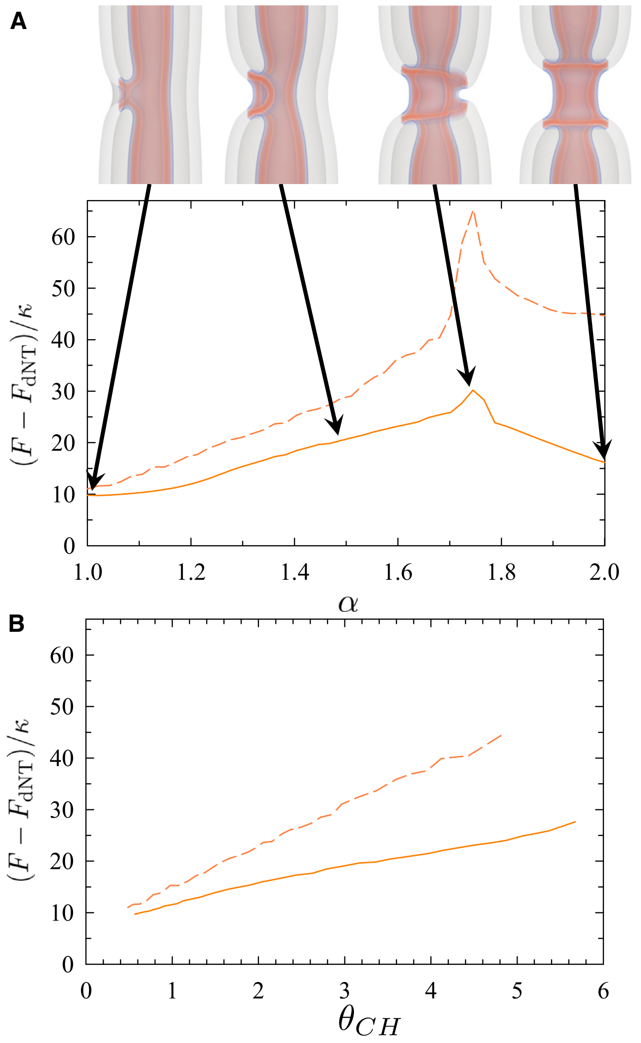

FIGURE 9 Free-energy along the MFEPs for the HD zipping shown in Fig. 8. Free energies are shown (*A*) as a function of, α, the order parameter derived from the string method and (*B*) θ_{CH}, the angle through which the HD has "zipped." θ_{CH} is only calculated while the HD is zipping, i.e., the data used in (*B*) is cut off at the more complicated final rupture of the outer membrane. Data are shown for (*dashed*) $\sigma = 0.129\kappa/d^2$ and (*solid*) $\sigma = 0.387\kappa/d^2$, free energies are given relative to that of a dNT, F_{dt} and key steps are illustrated for $\sigma = 0.129\kappa/d^2$.

Another possibility (not shown) is that the single-tube simply collapses and ruptures as in the simple mechanism presented earlier. The barrier to this is significantly larger than for any of the disconnection mechanisms presented. After the tube retracts, the structure continuously transforms into a pair of capped NTs (inner and outer) connected by a stalk. This transformation is shown in the supporting material.

Overview of possible pathways

We have presented a number of ways that a dNT can evolve under tension, broken into individual steps or stages. We now wish to combine the steps into an

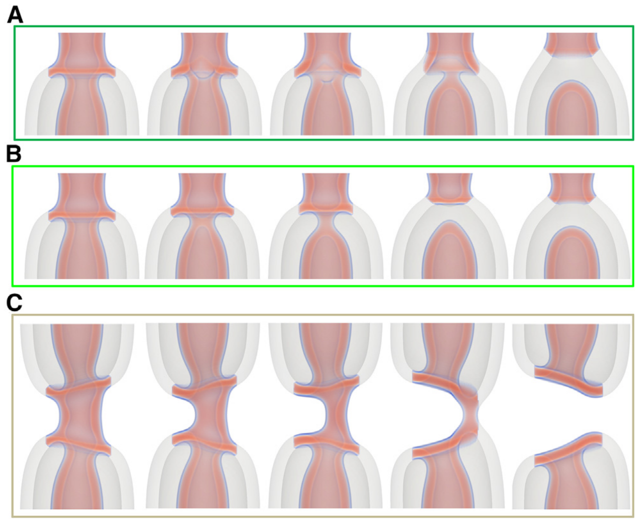

FIGURE 10 Intermediate configurations for the evolution of the CH state at a tension of $\sigma = 0.387\kappa/d^2$. (*A*) and (*B*) show two mechanisms, by which the inner membrane detaches from the outer membrane, resulting in a capped inner NT. (*C*) The rupture and unzipping of the connecting sNT. The outer tube in the double-tube region has been made transparent.

overall picture of membrane tube evolution. To clarify the possible processes, Fig. 12 summarizes potential transformation pathways of sNTs at high tension, and dNTs at high and low tensions, $\sigma = 0.387\kappa/d^2$ and $0.129\kappa/d^2$, respectively. sNT fission (Fig. 12 *A*) proceeds by partially collapsing the membrane (WLM) and subsequently rupturing the WLM.

The dNT system has many more degrees of freedom than the sNT system and a variety of possible pathways. At high tension, the system may collapse directly (canonical pathway) or using a stalk. The barrier to initially forming a stalk is lower than the canonical pathway. The stalk may catalyze inner-tube fission or may expand to form an HD. From there, it can either collapse (*pink* pathway) or zip (*orange* pathway). The barrier to zipping is significantly higher than the other options, thus the next state is likely the collapsed state.

To judge the likelihood of the multistep pathways, we combine the probabilities of each event along the pathway. The probability of a fluctuation with free-energy F_i occurring is proportional to e^{-F_i/k_BT}. The probability of a series of such events is proportional to $\prod_i e^{-F_i/k_BT} = e^{-\sum_i F_i/k_BT}$. We therefore choose the sum of the individual barriers to describe the probability of a given process. At high tension, the sum of the individual barriers is lowest for the direct (*black*) pathway, making it the most likely.

At low tension (Fig. 12 *C*), the barrier to directly collapsing a part of the inner membrane into a WLM becomes prohibitive, and stalk formation between the inner and outer membranes becomes more favorable by comparison. Once again, from here, the stalk may either follow the

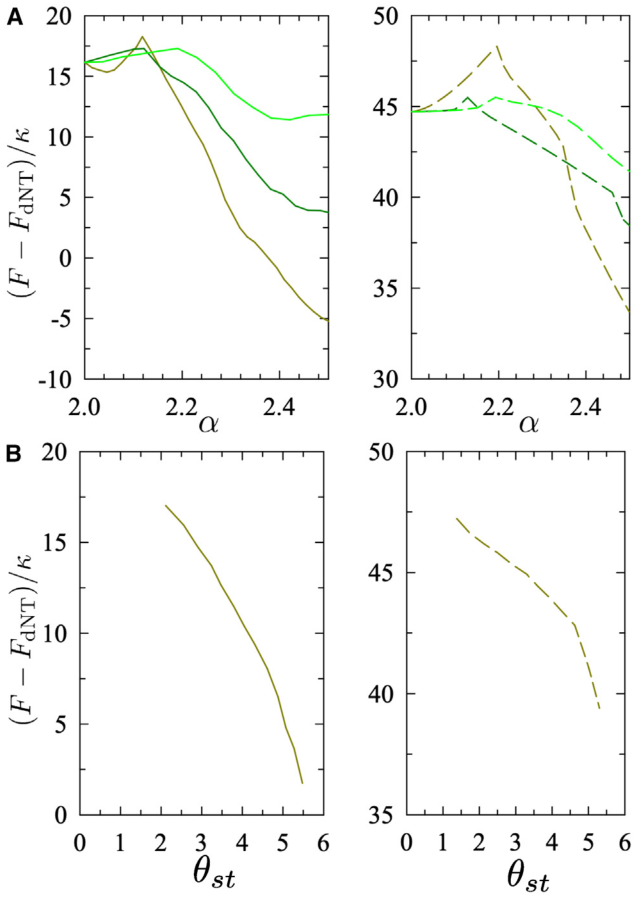

FIGURE 11 Free-energy along the MFEPs for the fission of the CH state. Plot colors are chosen to match those of Fig. 10, data are shown for (*left*, *solid*) $\sigma = 0.387\kappa/d^2$ and (*right*, *dashed*) $\sigma = 0.129\kappa/d^2$. Free energies are given relative to that of a dNT, $F_{\rm dt}$. Free energies are shown as functions of (*A*) α and (*B*) the inferred order parameter, θ_{st}, the angle through which the sNT has unzipped for the pathway shown in Fig. 10 *C*. θ_{st} is only calculated during the growth of the pore, i.e., after pore formation and before the final disconnection of the tubes.

upper branch and form an HD and then zip around to form the CH state (*orange*) or the inner membrane may disconnect via a WLM intermediate (*red*), i.e., transition to the lower branch in the diagram. The barrier to disconnecting and forming a partially collapsed (WLM) inner NT is substantially lower than zipping the HD. The system is therefore more likely to first connect the inner and outer membranes via a stalk, as an intermediate to forming a partially collapsed (WLM) inner NT, i.e., the inner and outer NTs will occasionally connect and disconnect via a stalk, until the connection leads to a partial collapse (WLM) of the inner NT. The latter then undergoes rupture. The outer membrane subsequently fissions via the mechanism in Fig. 12 *A*. This stalk-intermediate pathway has several stages at which either membrane may form transient pores, making it a potentially leaky pathway. This more thorough investigation thus agrees with the prediction of (26).

Comparison between SCFT and particle-based simulations

SCFT and the string method have allowed us to obtain a detailed picture of many membrane remodeling processes, including free-energy barriers that would be computationally prohibitive for direct particle-based simulations. This approach, however, merely relaxes the transformation pathway from an initial path into the nearest local MFEP. It may therefore fail to find even close-by (in configuration space) pathways with lower barriers. This is particularly important for cases like double-membrane remodeling, where there are many degrees of freedom, and it is practically impossible to examine every way that a membrane could rearrange. Combining our calculations with particle-based simulations can reveal transformation pathways that we may have missed, in addition to allowing us to validate our calculations, by verifying that the predicted evolution actually occurs.

SCFT and the particle-based simulations also differ in that the evolution of the particle-based simulations is locally mass conserving, unlike the SCFT evolution, which is governed by model A dynamics (77). It is possible to carry out dynamic SCFT calculations using model B; however, the implementation is more complicated and usually (when considering only (meta)stable states) the outcome is the same. For nonequilibrium behavior, as in the string method, the two models may in general produce different dynamics.

It would take prohibitively long to carry out simulations by simply initializing a sNT or dNT and waiting for the previously described processes to occur. A more productive approach is to use the fields from SCFT to initialize a starting configuration of the particle-based model and allow the system to evolve. We initialized SOMA simulations at key locations, such as metastable configurations or saddle-points on the free-energy landscape, and allowed them to relax. The setup and an example of subsequent evolution of the SOMA simulations are shown in Videos S5 and S6, respectively.

In each case, the system evolved by "descending" the pathway predicted by the string method. Side-by-side comparisons between SCFT and particle-based simulation are shown in Fig. 13, and further discussed below. The consistency suggests that the pathways are realistic, and are not sensitive to either fluctuations or the particular dynamical model employed.

A sNT partially collapses into a WLM, as illustrated in Fig. 13 *A*. This corresponds to the sNT pathway discussed earlier (Fig. 1). Unlike in the SCFT calculations, we do not observe the fission of the WLM connecting the two capped sNTs. In the SCFT calculations there is a constant tension along the NT, set by the chemical potential. By contrast, the particle-based simulations are conducted in the canonical ensemble, where we fix the number of lipids

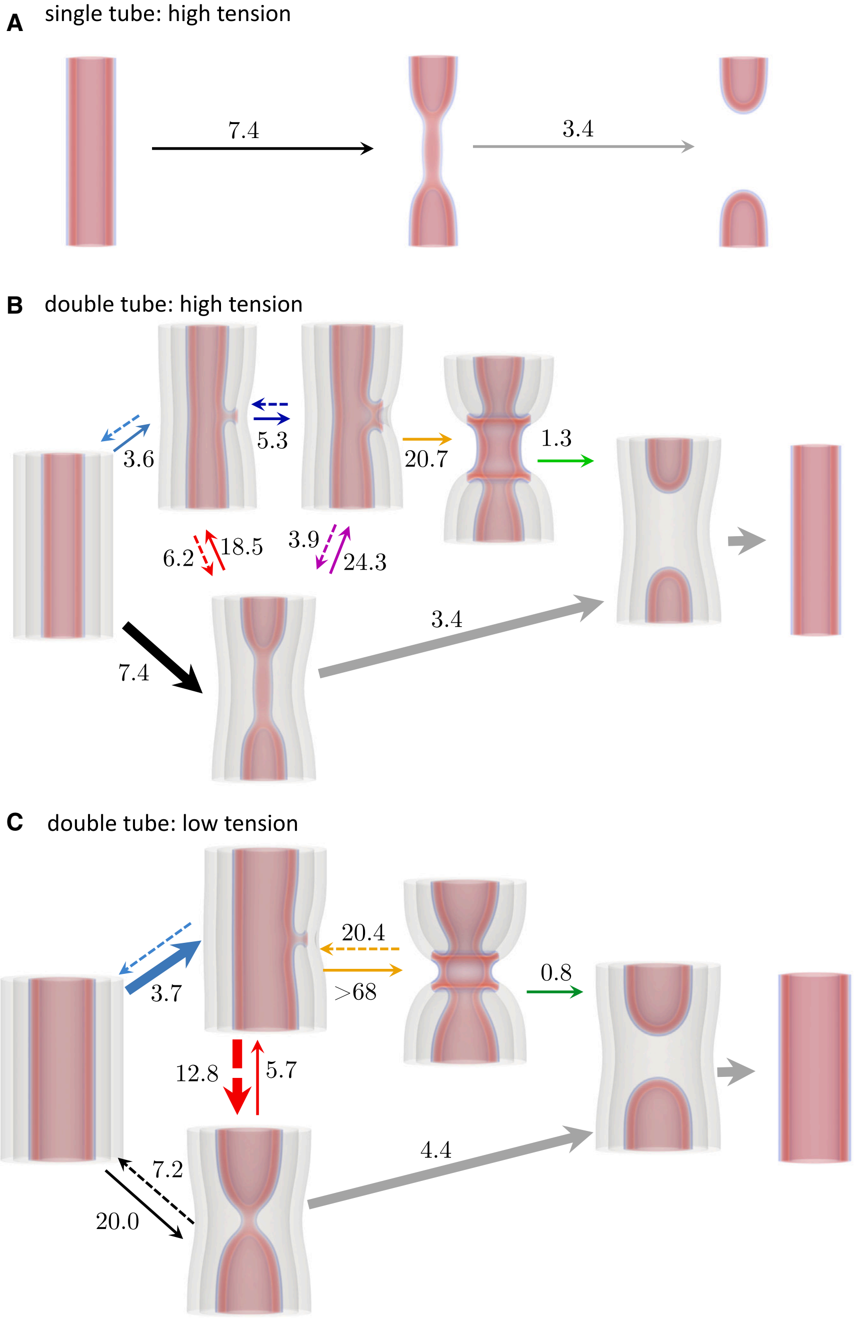

FIGURE 12 For a Figure360 author presentation of Fig. 12, see https://doi.org/10.1016/j.bpj.2024.10.009. Figure360▷
Illustration of the transition mechanisms with associated free-energy barriers, in units of κ. Colors correspond to the barriers used thus far in the paper. Arrows are shown for increasing α (*solid*) and decreasing α (*dashed*) directions, respectively. The most likely pathways to fission is highlighted with thick arrows. Pathways are shown for (*A*) sNTs at $\sigma = 0.387\kappa/d^2$, (*B*) dNTs at $\sigma = 0.387\kappa/d^2$, where sequential fission is more likely, and (*C*) dNTs at $\sigma = 0.129\kappa/d^2$, where hemifusion is more likely. The missing barriers on blue paths likely are nonzero but difficult to assess (see main text). Arrows going only one direction are transitions that appear irreversible, because the transition may proceed arbitrarily far in one direction, e.g., the growth of a WLM intermediate.

rather than the chemical potential. Partially collapsing the NT reduces the tension in the membrane resulting in insufficient tension to break the WLM.

If the system is initialized in a cylindrically hemifused state, it remains metastable for a short time, then the inner NT disconnects from the outer one via either of the predicted mechanisms, i.e., the transformation pathways illustrated in Fig. 10, *A* or *B* and corresponding to the dark and light green data in Fig. 11. The comparison between particle-based simulation and SCFT for these transformation pathways is shown in Fig. 13 *B*.

A partially zipped HD further unzips, decreasing the diaphragm that connects the inner and outer NTs, as illustrated in Fig. 13 *C*. This mechanism corresponds to the data shown in Fig. 9, along the direction of decreasing free-energy and decreasing α. The system remains metastable for a short time and then proceeds by the transformation pathways shown in Fig. 13, *D* or *E*. These pathways are the reverse of the blue and pink curves in Fig. 6, respectively. The HD may radially shrink, as in Fig. 13 *D*, collapsing into a stalk that connects the inner and outer membranes. Alternatively, the diaphragm may disconnect, as in Fig. 13 *E*,

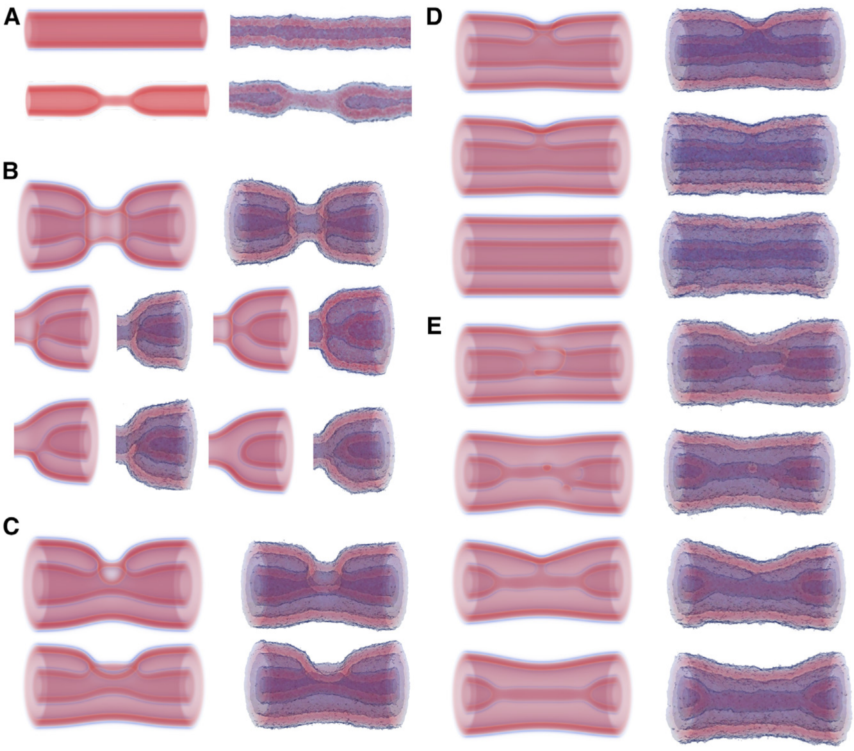

FIGURE 13 Comparison of configurations from SCFT calculations and particle-based simulations. Simulations were initialized using fields derived from SCFT strings (see Video S5) and, in each case, they either remained in a metastable state or followed the string derived from SCFT along the direction of decreasing free-energy. (*A*) The sNT partially collapses into a WLM. (*B*) Initialized with a cylindrically hemifused state, the inner tube disconnects from the sNT-dNT-junction by either (*left*) the mechanism in Fig. 9 *A* or (*right*) Fig. 9 *B*. (*C*) Starting from an HD, the diaphragm unzips, descending the curve in Fig. 9. The system then proceeds via one of the pathways in (*D*) and (*E*), which correspond to the blue and pink curves in Fig. 6, respectively. For pathway (*E*), side-by-side trajectories are shown in Video S6.

resulting in a partially collapsed (WLM) inner NT. This observation is consistent with our prediction that the stalk or HD is metastable and can act as an intermediate state toward the partial collapse of the inner NT into a WLM, along the pathway to fission.

The comparisons provide validation that the pathways that we have found are not artifacts of the additional approximations in SCFT. Another aspect of the kinetics is the transition rate. As discussed above, the barriers are too large to simply wait for each process to occur in particle-based simulations. A more promising approach is to combine the free-energy barrier and a kinetic coefficient to obtain the Kramers rate (78). We have calculated the former using SCFT. Although we present some kinetic information in the supporting material, obtaining the kinetic coefficients requires modifications to our particle-based simulation approach. One strategy is to use forward-flux sampling (79). This method breaks each process into many small subprocesses, calculates the time (thus rate) for the first subprocess, and the success probability (thus free-energy barrier) to carry out each subsequent subprocess. This is outside of the current scope and is left for future work.

DISCUSSION

Fusion and fission are indispensable and ubiquitous elements of intracellular membrane dynamics (80). Both control the membrane-barrier function and, hence, there has long been the question how to transform membrane topology without full-scale rupture and leakage (6,81). First, a highly curved lipid stalk was discovered and established, experimentally and theoretically, as the initial intermediate of leakless membrane fusion. Then, in 2003, Kozlovsky and Kozlov were the first to adapt this model to fission (4). In their study, the authors used elastic theory to analyze the transition from a constricted neck to a hypothetical HF intermediate (in the analogy to the fusion process, i.e., resembling a partially collapsed [WLM] NT). The work introduced the notion of critical curvature, at which the elastic energy of the neck becomes comparable with that of the WLM-like HF intermediate, thus favoring the transition. The HF intermediate was assumed to be unstable so that the HF-to-full-fission transition happened spontaneously.

Since then, membrane fission has been modeled using different phenomenological, mean field, and CG approaches. The use of CG simulations pointed to the metastable character of the HF intermediate (9,10), a discovery with mechanistic implications for the energy transduction by proteins during this type of membrane remodeling. Indeed, WLM-like HF intermediates were several times detected in protein-driven membrane transformations. Mattila et al. produced a truncated dynamin GTPase that mimicked the protein's activated state during its GTPase cycle, usually linked to the protein's fission activity (7). Upon incubation of the truncated protein with large unilamellar vesicles, the authors detected HF intermediates beneath the protein

scaffold. Similarly, Mizuno et al. detected protein-coated cylindrical lipid micelles upon membrane incubation with endophilin molecules (82). However, in none of these cases a bona fide fission reaction was reconstructed, as the protein coats stabilized the HF intermediate, precluding the characterization of topological transitions along the fission pathway.

The data presented here as well as in (26) provide additional experimental evidence that membrane fission proceeds through the formation of a metastable WLM-like HF intermediate, which then resolves into full fission (Fig. 1). The HF intermediate has the expected geometry, with its thickness being similar to that of a lipid bilayer. Although not discussed therein, the statistics presented in (26) confirmed the earlier theoretical predictions about the geometry of membrane fission, as membrane remodeling proceeds only upon reaching the critical radius value in the range of the bilayer thickness (4).

To achieve fission in the pure lipid system we used membrane tension. In the cell, tension is often related to membrane dynamics, action of motor proteins, and cytoskeleton acting against adhesion and friction forces (83). The balance between the membrane-pulling forces and friction drag constitutes the constriction-by-friction mechanism of membrane fission where local membrane tension increases dramatically causing membrane HF and/or pore formation (18,20). Here, we reconstructed such dynamically elevated tension. The tension-driven constriction followed by HF was detected by cryo-EM. Both short HF intermediates and metastable WLMs were detected (Fig. 1 *B*). We found that HF becomes spontaneous at values of membrane tension that are at least one order of magnitude higher than that reported for cellular plasma membrane ($10^{-1} - 10^{-2}$ mN/m, (84)). High lateral membrane tensions, required to produce such a high membrane curvature, have been a long-known cause of poration and lysis of planar lipid bilayers (11). Importantly, using SCFT modeling, we showed that the HF pathway would be more likely than pore nucleation even at such high membrane tension (Fig. 1). High membrane tension also favors the formation and extension of the metastable WLM (Fig. 1).

Membrane fission, however, is not limited to single membranes. One of the best-known examples of double-membrane fission in cells is that of the mitochondrion. While the protein machinery involved in this process has been extensively explored, surprisingly little is known about the free-energy landscape of double-membrane remodeling itself (27,27,85–91). By direct extrapolation from single-membrane remodeling, we can expect two outcomes upon double-membrane constriction: sequential fission of the inner and then the outer membranes following the single-membrane pathway, or the fusion of the outer membrane to the inner one due to curvature stress. Consistent with previous work (26), we find the latter pathway for low tensions, $\sim 1 - 2$ mN/m, already associated with curvature instabilities in single-membrane systems. At higher tensions, however, the sequential fission pathway is the likely outcome of constriction.

The details of the membrane rearrangements were further analyzed by SCFT. The first step in the reaction is the formation of a close contact area between the membranes. Next, a stalk and then an HD is nucleated and, in some cases, leads to formation of an extended HD encircling the dNT, i.e., the CH state. Intriguingly, such restricted HF may result in a quick exchange of lipids, such as cardiolipin, between the outer and inner membrane of the mitochondrion (92). HF may resolve into full fusion (81,93); however, this transformation from dNT to sNT does not occur in our simulations. Instead, in the absence of cofactors, the HD rather decays via redetachment of the inner NT from the outer one. This process is accompanied by the formation of several pores. Such poration may result in depolarization of the mitochondrial inner membrane, a process well known to complement mitochondrial fission (87,89).

Of note, lipid rearrangements in fission and fusion are closely related both in vivo and via the pathways studied in this work. While we have been focused on the fission of membrane tubes, we can also consider the antipode transformation by simply looking at our MFEPs in the "reverse" (decreasing α). Barriers to some steps in the fusion process can be inferred from the free-energy profiles (Figs. 6, 9, and 11), and are partially illustrated in Fig. 12. Some reverse barriers, however, are omitted as they are difficult to obtain from our calculations. For example, as currently implemented, since the NTs are under tension, they simply retract as far as they can from one another upon fission. To reverse the process, one would need to fix the capped NTs at a set distance. In cells, this process is mediated by GTP-powered docking proteins (85). Based on the low barriers, once the membranes are brought together, the fusion process would readily follow the reversed path of fission. This observation might be of importance for mitochondrial fusion and fission known to be synchronized and colocalized in time and space (89,90). Analysis of mitochondrial remodeling indicate that fusion may trigger fission, but fusion is not affected by fission (90). Interestingly, the barriers in the fusion direction, shown in Fig. 12, suggest that the complex pathway may occur more readily when membranes are fusing, particularly under low tension.

The free-energy barriers reported here are valid for homogeneous lipid systems under high lateral tension, such as the one produced by the hydrodynamic flow in our cryo-EM experiments. While cellular membranes are at considerably lower tension on average, local spikes of tension during fast membrane transformations can lead to extreme membrane constriction and instabilities similar to those described here (18,20). Biological systems may also employ a variety of strategies to decrease the free-energy barriers and select the preferred transformation pathway. Among

these strategies are protein catalysis of membrane remodeling. A great example of such catalyzers are the members of the dynamin superfamily of GTPases that transform the energy of GTP into membrane curvature stress both in fusion and in fission. Interestingly, many fusion and fission dynamins colocalize at the mitochondrial fission site (94). Another factor that may alter the outcome of the remodeling pathway is membrane asymmetry. Biological membranes present compositional asymmetry both between leaflets and between different membranes (95). Such asymmetry confers distinct mechanical properties onto each membrane, thus potentially shifting the free-energy barriers for the transformations we predicted in our model system. Finally, lipid and protein redistribution along the curvature gradients may also help remodeling (96–98).

Each one of the cited factors may alter the way that membranes rearrange during fission and fusion, possibly giving rise to new transformation pathways. However, as the most energetically expensive transformation steps involve locally constricting the (outer) membrane, it seems likely that a simple mechanical constriction can drastically lower the free-energy barriers, while leaving the details of the rearrangements mostly intact. The linearity of the required free energy with constriction radius implies that a constant force is required regardless of the membrane radius.

Future work should therefore explore the effect of constriction by cellular factors on the favorability of competing transformation pathways in membrane remodeling, particularly in fission and fusion of double-membrane systems. The main contribution from constriction proteins, such as dynamin, is likely simply a way to apply the required constriction force, but there may also be specific effects due to the particular membrane interactions, for example, splaying of headgroups due to the insertion of dynamin's PH domains between the headgroups. Furthermore, although zipping of the HD around the double tube, to produce the cylindrically hemifused state, is prohibitively expensive, perhaps it may be possible with the aid of constriction.

Finally, it should be noted that our SCFT implementation used in the majority of this work leads to each membrane having the same tension. This is not generally the case, as each membrane, and indeed leaflet, may be attached to a different reservoir. It does, however, create concentric tubes, which are in close contact, as seen in the experiments, and which we could expect from external sources of constriction, such as helical constriction proteins around the outer tube. We have briefly explored, in the supporting material, an approach to calculating corrections due to different membrane tensions.

CONCLUSION

In this study, we have combined SCFT and the string method to identify optimal fission pathways in single- and double-membrane tubes. We have further validated these pathways using cryo-EM experiments, demonstrating the robustness of our approach.

Our results suggest that the free-energy barriers to single- and double-membrane fission are sensitive to membrane tension. In double-membrane tubes, there are several possible pathways, and the dominant pathway can be controlled by the membrane tension. We describe two competing pathways to fission. At very high tension, the more likely mechanism involves the partial collapses of the inner tube, into a WLM, which then ruptures, resulting in two capped tubes. This occurs without interaction with the outer membrane. The outer membrane then follows similarly. This pathway is nonleaky, i.e., the solvent inside the inner membrane, between the membranes and outside the outer membrane never mix. At lower, more physiologically relevant tension, however, the barrier to forming a WLM becomes prohibitive, and instead, the inner and outer membranes fuse, which then catalyzes fission via a more complicated pathway. This pathway is leaky as pores form close to the fusion sites.

In addition to these fission pathways, we explore a variety of other mechanisms by which double tubes may evolve. Except for the case of very high tensions, the free-energy barriers that we obtain (typically $O(10\kappa)$) are larger than we would expect for biological processes that occur frequently. This is consistent with the need for constricting proteins to catalyze these processes in real biological systems. Further work is required to understand how these proteins affect the mechanism and free-energy barriers for the processes presented here.

ACKNOWLEDGMENTS

We thank Vadim Frolov for many helpful discussions. Financial support has been provided by the Deutsche Forschungsgemeinschaft within CRC 1286 TP C06. A.V.S. was supported by grant PGC2018-099971-B-I00 funded by MCINAEI 10.13039501100011033 and "ERDF A way of making Europe" through the "European Union", and by Basque Government Grant IT1625-22. The authors gratefully acknowledge the Gauss Centre for Supercomputing e.V. (www.gauss-centre.eu) for funding this research project by providing computing time through the John von Neumann Institute for Computing (NIC) on GCS Supercomputer JUWELS at the Jülich Supercomputing Centre (JSC). The authors are grateful to the Electron Microscopy and Crystallography platform of the CIC bioGUNE and the Basque Resource for Electron Microscopy for providing access to cryo-EM sample preparation and analysis equipment.

AUTHOR CONTRIBUTIONS

R.K.W.S., A.V.S., and M.M. designed the research. R.K.W.S. performed the theoretical research and analyzed data. I.S.-P. and A.V.S. performed the experimental research. A.V.S. analyzed the experimental data. R.K.W.S., A.V.S., and M.M. discussed the results and wrote the paper.

DECLARATION OF INTERESTS

The authors declare no competing interests.

SUPPORTING MATERIAL

Supporting material can be found online at https://doi.org/10.1016/j.bpj.2024.10.009.

REFERENCES

1. Renard, H.-F., L. Johannes, and P. Morsomme. 2018. Increasing Diversity of Biological Membrane Fission Mechanisms. *Trends Cell Biol.* 28:274–286.
2. Frolov, V. A., A. Escalada, …, A. V. Shnyrova. 2015. Geometry of membrane fission. *Chem. Phys. Lipids.* 185:129–140.
3. Hochmuth, R. M., and E. A. Evans. 1982. Extensional flow of erythrocyte membrane from cell body to elastic tether. I. Analysis. *Biophys. J.* 39:71–81.
4. Kozlovsky, Y., and M. M. Kozlov. 2003. Membrane Fission: Model for Intermediate Structures. *Biophys. J.* 85:85–96.
5. Bashkirov, P. V., S. A. Akimov, …, V. A. Frolov. 2008. GTPase cycle of dynamin is coupled to membrane squeeze and release, leading to spontaneous fission. *Cell.* 135:1276–1286. https://www.sciencedirect.com/science/article/pii/S0092867408015031.
6. Kozlov, M. M., H. T. McMahon, and L. V. Chernomordik. 2010. Protein-driven membrane stresses in fusion and fission. *Trends Biochem. Sci.* 35:699–706.
7. Mattila, J.-P., A. V. Shnyrova, …, V. A. Frolov. 2015. A hemi-fission intermediate links two mechanistically distinct stages of membrane fission. *Nature.* 524:109–113.
8. Zhang, G., and M. Müller. 2017. Rupturing the hemi-fission intermediate in membrane fission under tension: Reaction coordinates, kinetic pathways, and free-energy barriers. *J. Chem. Phys.* 147:064906.
9. Deserno, M. 2009. Mesoscopic Membrane Physics: Concepts, Simulations, and Selected Applications. *Macromol. Rapid Commun.* 30:752–771.
10. Fuhrmans, M., and M. Müller. 2015. Coarse-grained simulation of dynamin-mediated fission. *Soft Matter.* 11:1464–1480.
11. Evans, E., V. Heinrich, …, W. Rawicz. 2003. Dynamic Tension Spectroscopy and Strength of Biomembranes. *Biophys. J.* 85:2342–2350.
12. Marrink, S. J., A. H. de Vries, and A. E. Mark. 2004. Coarse Grained Model for Semiquantitative Lipid Simulations. *J. Phys. Chem. B.* 108:750–760.
13. Marrink, S. J., H. J. Risselada, …, A. H. de Vries. 2007. The MARTINI Force Field: Coarse Grained Model for Biomolecular Simulations. *J. Phys. Chem. B.* 111:7812–7824.
14. Sandre, O., L. Moreaux, and F. Brochard-Wyart. 1999. Dynamics of transient pores in stretched vesicles. *Proc. Natl. Acad. Sci. USA.* 96:10591–10596.
15. Müller, M., and M. Schick. 1996. Structure and nucleation of pores in polymeric bilayers: A Monte Carlo simulation. *J. Chem. Phys.* 105:8282–8292.
16. Morlot, S., V. Galli, …, A. Roux. 2012. Membrane Shape at the Edge of the Dynamin Helix Sets Location and Duration of the Fission Reaction. *Cell.* 151:619–629.
17. Shnyrova, A. V., P. V. Bashkirov, …, V. A. Frolov. 2013. Geometric Catalysis of Membrane Fission Driven by Flexible Dynamin Rings. *Science.* 339:1433–1436. https://science.sciencemag.org/content/339/6126/1433.abstract.
18. Espadas, J., D. Pendin, …, A. Daga. 2019. Dynamic constriction and fission of endoplasmic reticulum membranes by reticulon. *Nat. Commun.* 10:5327.
19. Mahecic, D., L. Carlini, …, S. Manley. 2021. Mitochondrial membrane tension governs fission. *Cell Rep.* 35:108947.
20. Simunovic, M., J.-B. Manneville, …, A. Callan-Jones. 2017. Friction Mediates Scission of Tubular Membranes Scaffolded by BAR Proteins. *Cell.* 170:172–184.e11.
21. Tolpekina, T. V., W. K. den Otter, and W. J. Briels. 2004. Nucleation free energy of pore formation in an amphiphilic bilayer studied by molecular dynamics simulations. *J. Chem. Phys.* 121:12060–12066.
22. Ting, C. L., D. Appelö, and Z.-G. Wang. 2011. Minimum Energy Path to Membrane Pore Formation and Rupture. *Phys. Rev. Lett.* 106:309.
23. Hu, G.-B. 2014. Whole Cell Cryo-Electron Tomography Suggests Mitochondria Divide by Budding. *Microsc. Microanal.* 20:1180–1187.
24. Mageswaran, S. K., D. A. Grotjahn, …, G. J. Jensen. 2021. Nanoscale details of mitochondrial fission revealed by cryo-electron tomography. Preprint at bioRxiv. https://doi.org/10.1101/2021.12.13.472487.
25. Zhang, L., S. Trushin, …, E. Trushina. 2016. Altered brain energetics induces mitochondrial fission arrest in Alzheimer's Disease. *Sci. Rep.* 6:18725.
26. Spencer, R. K. W., I. Santos-Pérez, …, M. Müller. 2024. Membrane fission via transmembrane contact. *Nat. Commun.* 15:2793.
27. Suen, D.-F., K. L. Norris, and R. J. Youle. 2008. Mitochondrial dynamics and apoptosis. *Genes Dev.* 22:1577–1590.
28. Suhaili, S. H., H. Karimian, …, M.-I. Aguilar. 2017. Mitochondrial outer membrane permeabilization: a focus on the role of mitochondrial membrane structural organization. *Biophys. Rev.* 9:443–457.
29. Gruhn, T., T. Franke, …, R. Lipowsky. 2007. Novel Method for Measuring the Adhesion Energy of Vesicles. *Langmuir.* 23:5423–5429.
30. Noguchi, H. 2009. Membrane Simulation Models from Nanometer to Micrometer Scale. *J. Phys. Soc. Jpn.* 78:041007.
31. Kawamoto, S., M. L. Klein, and W. Shinoda. 2015. Coarse-grained molecular dynamics study of membrane fusion: Curvature effects on free energy barriers along the stalk mechanism. *J. Chem. Phys.* 143:243112.
32. Li, Z.-L., H.-M. Ding, and Y.-Q. Ma. 2016. Interaction of peptides with cell membranes: insights from molecular modeling. *J. Phys. Condens. Matter.* 28:083001.
33. Ghosh, R., V. Satarifard, …, R. Lipowsky. 2021. Budding and Fission of Nanovesicles Induced by Membrane Adsorption of Small Solutes. *ACS Nano.* 15:7237–7248.
34. Maragliano, L., A. Fischer, …, G. Ciccotti. 2006. String method in collective variables: Minimum free energy paths and isocommittor surfaces. *J. Chem. Phys.* 125:024106.
35. E, W., W. Ren, and E. Vanden-Eijnden. 2007. Simplified and improved string method for computing the minimum energy paths in barrier-crossing events. *J. Chem. Phys.* 126:164103.
36. Ting, C. L., and Z.-G. Wang. 2012. Minimum free energy paths for a nanoparticle crossing the lipid membrane. *Soft Matter.* 8:12066–12071.
37. Müller, M., Y. G. Smirnova, …, A.-C. Shi. 2012. Transition Path from Two Apposed Membranes to a Stalk Obtained by a Combination of Particle Simulations and String Method. *Phys. Rev. Lett.* 108:228103.
38. Li, J., H. Zhang, …, A.-C. Shi. 2013. Emergence and stability of intermediate open vesicles in disk-to-vesicle transitions. *Phys. Rev.* 88:012719.
39. Kozlov, M. M., and W. Helfrich. 1992. Effects of a cosurfactant on the stretching and bending elasticities of a surfactant monolayer. *Langmuir.* 8:2792–2797.
40. Kozlovsky, Y., and M. M. Kozlov. 2002. Stalk Model of Membrane Fusion: Solution of Energy Crisis. *Biophys. J.* 82:882–895.
41. Kozlovsky, Y., L. V. Chernomordik, and M. M. Kozlov. 2002. Lipid Intermediates in Membrane Fusion: Formation, Structure, and Decay of Hemifusion Diaphragm. *Biophys. J.* 83:2634–2651.
42. Capraro, B. R., Y. Yoon, …, T. Baumgart. 2010. Curvature Sensing by the Epsin N-Terminal Homology Domain Measured on Cylindrical Lipid Membrane Tethers. *J. Am. Chem. Soc.* 132:1200–1201.
43. Bashkirov, P. V., K. V. Chekashkina, …, V. A. Frolov. 2011. Variation of lipid membrane composition caused by strong bending. *Biochem. Moscow. Suppl. Ser. A.* 5:205–211.
44. Deserno, M. 2015. Fluid lipid membranes: From differential geometry to curvature stresses. *Chem. Phys. Lipids.* 185:11–45.

45. Ryham, R. J., T. S. Klotz, …, F. S. Cohen. 2016. Calculating Transition Energy Barriers and Characterizing Activation States for Steps of Fusion. *Biophys. J.* 110:1110–1124.

46. Terzi, M. M., M. F. Ergüder, and M. Deserno. 2019. A consistent quadratic curvature-tilt theory for fluid lipid membranes. *J. Chem. Phys.* 151:164108.

47. Daoulas, K. C., and M. Müller. 2010. Comparison of Simulations of Lipid Membranes with Membranes of Block Copolymers. Springer Berlin Heidelberg, Berlin, Heidelberg, pp. 43–85. https://doi.org/10.1007/978-3-642-10479-4_7.

48. Smirnova, Y. G., M. Fuhrmans, …, M. Müller. 2015. Free-energy calculation methods for collective phenomena in membranes. *J. Phys. D Appl. Phys.* 48:343001.

49. Smirnova, Y. G., H. J. Risselada, and M. Müller. 2019. Thermodynamically reversible paths of the first fusion intermediate reveal an important role for membrane anchors of fusion proteins. *Proc. Natl. Acad. Sci. USA.* 116:2571–2576.

50. Katsov, K., M. Müller, and M. Schick. 2004. Field Theoretic Study of Bilayer Membrane Fusion. I. Hemifusion Mechanism. *Biophys. J.* 87:3277–3290.

51. Katsov, K., M. Müller, and M. Schick. 2005. Fusion of biological membranes. *Pramana.* 64:1127–1134.

52. Katsov, K., M. Müller, and M. Schick. 2006. Field Theoretic Study of Bilayer Membrane Fusion: II. Mechanism of a Stalk-Hole Complex. *Biophys. J.* 90:915–926.

53. Müller, M., K. Katsov, and M. Schick. 2006. Biological and synthetic membranes: What can be learned from a coarse-grained description? *Phys. Rep.* 434:113–176.

54. Li, J., K. A. Pastor, …, J. Zhou. 2013. Elastic properties and line tension of self-assembled bilayer membranes. *Phys. Rev.* 88:012718.

55. Dehghan, A., K. A. Pastor, and A.-C. Shi. 2015. Line tension of multicomponent bilayer membranes. *Phys. Rev.* 91:022713. https://doi.org/10.1103/PhysRevE.91.022713.

56. Zhang, P.-W., and A.-C. Shi. 2015. Application of self-consistent field theory to self-assembled bilayer membranes. *Chin. Phys. B.* 24:128707.

57. Ting, C. L., and M. Müller. 2017. Membrane stress profiles from self-consistent field theory. *J. Chem. Phys.* 146:104901.

58. Cheng, X., L. Lin, …, A.-C. Shi. 2010. Nucleation of Ordered Phases in Block Copolymers. *Phys. Rev. Lett.* 104:148301.

59. Ting, C. L., N. Awasthi, …, J. S. Hub. 2018. Metastable Prepores in Tension-Free Lipid Bilayers. *Phys. Rev. Lett.* 120:128103. https://doi.org/10.1103/PhysRevLett.120.128103.

60. Han, Y., Z. Xu, …, L. Zhang. 2020. Pathways connecting two opposed bilayers with a fusion pore: a molecularly-informed phase field approach. *Soft Matter.* 16:366–374.

61. Schindelin, J., I. Arganda-Carreras, …, A. Cardona. 2012. Fiji: an open-source platform for biological-image analysis. *Nat. Methods.* 9:676–682.

62. Fredrickson, G. 2006. The Equilibrium Theory of Inhomogeneous Polymers (International Series of Monographs on Physics). Oxford University Press, USA.

63. G. Gompper and M. Schick, eds. (2006). Self-consistent Field Theory and its Applications, Soft Matter, Volume 1: Polymer Melts and Mixtures, Volume 1 of *Soft Matter* Polymer Melts and Mixtures, pp. 87–178.

64. Spencer, R. K. W., Y. G. Smirnova, …, M. Müller. 2024. Transient pores in hemifusion diaphragms. *Biophys. J.* 123:2455–2475.

65. Ranjan, A., J. Qin, and D. C. Morse. 2008. Linear Response and Stability of Ordered Phases of Block Copolymer Melts. *Macromolecules.* 41:942–954.

66. E, W., W. Ren, and E. Vanden-Eijnden. 2002. String method for the study of rare events. *Phys. Rev. B.* 66:052301.

67. Weinan, E., W. Ren, and E. Vanden-Eijnden. 2005. Finite Temperature String Method for the Study of Rare Events. *J. Phys. Chem. B.* 109:6688–6693.

68. Vanden-Eijnden, E. 2006. Towards a Theory of Transition Paths. *J. Stat. Phys.* 123:503–523.

69. Maragliano, L., and E. Vanden-Eijnden. 2007. On-the-fly string method for minimum free energy paths calculation. *Chem. Phys. Lett.* 446:182–190.

70. E, W., and E. Vanden-Eijnden. 2010. Transition-Path Theory and Path-Finding Algorithms for the Study of Rare Events. *Annu. Rev. Phys. Chem.* 61:391–420.

71. Schneider, L., and M. Müller. 2019. Multi-architecture Monte-Carlo (MC) simulation of soft coarse-grained polymeric materials: SOft coarse grained Monte-Carlo Acceleration (SOMA). *Comput. Phys. Commun.* 235:463–476.

72. Kučerka, N., M.-P. Nieh, and J. Katsaras. 2011. Fluid phase lipid areas and bilayer thicknesses of commonly used phosphatidylcholines as a function of temperature. *Biochim. Biophys. Acta Biomembr.* 1808:2761–2771.

73. Morris, C. E., and U. Homann. 2001. Cell Surface Area Regulation and Membrane Tension. *J. Membr. Biol.* 179:79–102.

74. Djakbarova, U., Y. Madraki, …, C. Kural. 2021. Dynamic interplay between cell membrane tension and clathrin-mediated endocytosis. *Biol. Cell.* 113:344–373.

75. Müller, M., K. Katsov, and M. Schick. 2003. A New Mechanism of Model Membrane Fusion Determined from Monte Carlo Simulation. *Biophys. J.* 85:1611–1623. https://linkinghub.elsevier.com/retrieve/pii/S0006349503745925.

76. Risselada, H. J., G. Marelli, …, M. Müller. 2012. Line-Tension Controlled Mechanism for Influenza Fusion. *PLoS One.* 7:e38302.

77. Provatas, N., and K. Elder. 2010. Nonequilibrium Dynamics. John Wiley & Sons, Ltd, pp. 33–56 chapter 4. https://doi.org/10.1002/9783527631520.ch4.

78. Kramers, H. 1940. Brownian motion in a field of force and the diffusion model of chemical reactions. *Physica.* 7:284–304.

79. Allen, R. J., C. Valeriani, and P. Rein Ten Wolde. 2009. Forward flux sampling for rare event simulations. *J. Phys. Condens. Matter.* 21:463102.

80. Wu, L.-G., E. Hamid, …, H.-C. Chiang. 2014. Exocytosis and Endocytosis: Modes, Functions, and Coupling Mechanisms. *Annu. Rev. Physiol.* 76:301–331.

81. Zhao, W.-D., E. Hamid, …, L.-G. Wu. 2016. Hemi-fused structure mediates and controls fusion and fission in live cells. *Nature.* 534:548–552.

82. Mizuno, N., C. C. Jao, …, A. C. Steven. 2010. Multiple Modes of Endophilin-mediated Conversion of Lipid Vesicles into Coated Tubes Implications for Synaptic Endocytosis. *J. Biol. Chem.* 285:23351–23358.

83. Pontes, B., P. Monzo, and N. C. Gauthier. 2017. Membrane tension: A challenging but universal physical parameter in cell biology. *Semin. Cell Dev. Biol.* 71:30–41.

84. Sheetz, M. P., and J. Dai. 1996. Modulation of membrane dynamics and cell motility by membrane tension. *Trends Cell Biol.* 6:85–89.

85. Chen, H., S. A. Detmer, …, D. C. Chan. 2003. Mitofusins Mfn1 and Mfn2 coordinately regulate mitochondrial fusion and are essential for embryonic development. *J. Cell Biol.* 160:189–200.

86. Otera, H., N. Ishihara, and K. Mihara. 2013. New insights into the function and regulation of mitochondrial fission. *Biochim. Biophys. Acta.* 1833:1256–1268.

87. Lee, H., and Y. Yoon. 2014. Transient Contraction of Mitochondria Induces Depolarization through the Inner Membrane Dynamin OPA1 Protein. *J. Biol. Chem.* 289:11862–11872.

88. Lee, J. E., L. M. Westrate, …, G. K. Voeltz. 2016. Multiple dynamin family members collaborate to drive mitochondrial division. *Nature.* 540:139–143. https://www.nature.com/articles/nature20555.

89. Abrisch, R. G., S. C. Gumbin, …, G. K. Voeltz. 2020. Fission and fusion machineries converge at ER contact sites to regulate mitochondrial morphology. *J. Cell Biol.* 219:e201911122.

90. Chiu, Y.-H., S.-C. A. Lin, …, C.-J. Li. 2021. Molecular Machinery and Pathophysiology of Mitochondrial Dynamics. *Front. Cell Dev. Biol.* 9:743892.

91. Al Ojaimi, M., A. Salah, and A. W. El-Hattab. 2022. Mitochondrial Fission and Fusion: Molecular Mechanisms, Biological Functions, and Related Disorders. *Membranes.* 12:893.

92. Zhang, Q., Y. Tamura, …, H. Sesaki. 2014. Biosynthesis and roles of phospholipids in mitochondrial fusion, division and mitophagy. *Cell. Mol. Life Sci.* 71:3767–3778.

93. Chernomordik, L. V., and M. M. Kozlov. 2005. Membrane Hemifusion: Crossing a Chasm in Two Leaps. *Cell.* 123:375–382.

94. Praefcke, G. J. K., and H. T. McMahon. 2004. The dynamin superfamily: universal membrane tubulation and fission molecules? *Nat. Rev. Mol. Cell Biol.* 5:133–147.

95. Basu Ball, W., J. K. Neff, and V. M. Gohil. 2018. The role of nonbilayer phospholipids in mitochondrial structure and function. *FEBS Lett.* 592:1273–1290.

96. Tian, A., and T. Baumgart. 2009. Sorting of Lipids and Proteins in Membrane Curvature Gradients. *Biophys. J.* 96:2676–2688.

97. Lu, C.-H., K. Pedram, …, B. Cui. 2022. Membrane curvature regulates the spatial distribution of bulky glycoproteins. *Nat. Commun.* 13:3093.

98. Bashkirov, P. V., P. I. Kuzmin, …, V. A. Frolov. 2022. Molecular Shape Solution for Mesoscopic Remodeling of Cellular Membranes. *Annu. Rev. Biophys.* 51:473–497.

Biophysical Journal, Volume *123*

Supplemental information

Fission of double-membrane tubes under tension

Russell K.W. Spencer, Isaac Santos-Pérez, Anna V. Shnyrova, and Marcus Müller

Supplementary Information for

"Fission of Double-Membrane Tubes under Tension"

Russell K. W. Spencer[1], Isaac Santos-Perez[2], Anna V. Shnyrova[3,4], Marcus Müller[1]

[1]Institute for Theoretical Physics, Georg-August University, 37077 Göttingen, Germany
[2]Electron Microscopy and Crystallography, Center for Cooperative Research in Biosciences (CIC bioGUNE), Bizkaia Science and Technology Park Bld 800, 48160-Derio, Bizkaia, Spain
[3]Instituto Biofisika (CSIC, UPV/EHU), Barrio Sarriena, 48940 Leioa, Spain
[4]Department of Biochemistry and Molecular Biology, University of the Basque Country, 48940 Leioa, Spain

E-mail: russell.spencer@theorie.physik.uni-goettingen.de, anna.shnyrova@ehu.eus, mmueller@theorie.physik.uni-goettingen.de

OBSERVED WLM

An overview of the experimental setup as well as a post-fission stable WLM are shown in Figure S1.

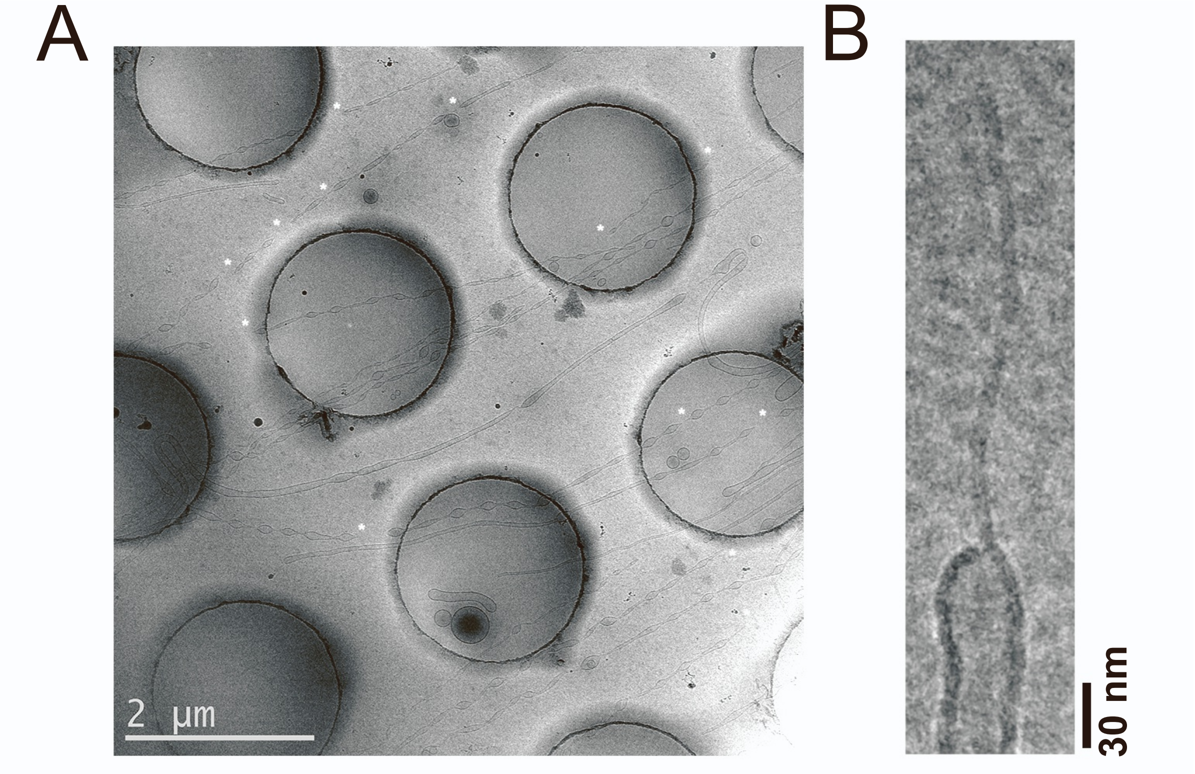

Figure S1: CryoEM of sNTs. A. Low magnification image of the sNTs obtained by the methods explained in Figure 1A. Asterisks indicate sites of sNT fission. B. Stable WLM upon sNT fission as observed by cryoEM.

PHASE BEHAVIOR

In order to study the rearrangement of lipid membranes using SCFT, we start by calculating the equilibrium phase behavior, in terms of the chemical potential of the lipids relative to the solvent, $\mu \equiv \mu_\ell - \mu_s$ and composition of the lipids, f, defined as the molecular fraction of a lipid tail. The former controls the membrane tension and the latter controls the spontaneous curvature of a lipid monolayer. The phase diagram, shown in Figure S2, is calculated by comparing the grandcanonical free energies of different structures.

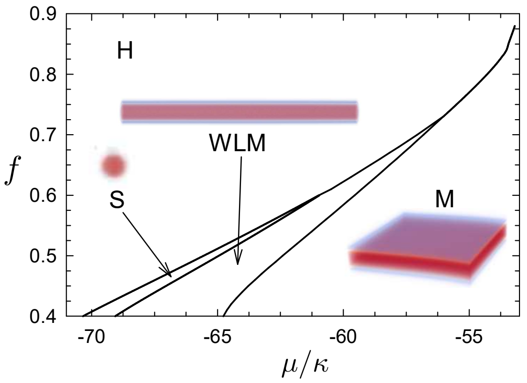

Figure S2: Phase behavior of lipids in solvent is shown in terms of the fractional composition of the lipid, f, and exchange chemical potential, $\mu = \mu_\ell - \mu_s$, of the lipid relative to that of the solvent. We consider the phases: homogeneous solution (H), spherical micelles (S), cylindrical wormlike micelles (WLM); and membranes (M).

In addition to the membrane structures that interest us, we also find spherical and worm-like micelles. The free-energy calculations were performed for a single spherical micelle (S), worm-like micelle (WLM), and membrane (M), coexisting with a homogeneous solution. The curves indicate where forming a S, WLM or M first lowers the free energy at the given f and μ.

The simplest form of M is a flat planar membrane, however, other possibilities include membrane nanotubes (NTs) and vesicles. The geometry (*e.g.* NT or vesicle diameter) is controlled by additional factors, such as tension, spontaneous curvature and penalties to bending. It is also possible for multiple structures to coexist, as in the case of double-membrane nanotubes (dNTs), which will be discussed later. In this case, interactions between membranes, such as hydration repulsion, also become relevant.

Although S and WLM are no longer stable at large f, they remain metastable. The WLM-M coexistence curve meets the H-M coexistence curve at an angle and can be continued to larger f. This is to say that there is a region on the low μ side of the H-M curve where WLM have a lower free energy than membranes, though both have a higher free energy than H. A membrane under tension (lower μ than coexistence) may thus lower its free energy by converting to a WLM, before lowering the free energy further by dissolving the WLM into H.

As described in the main text, throughout this work, unless otherwise stated, we will use $\chi N = 30$ and $f = 0.8$. The constants that we derive, such as the ordering transition, bending and splay moduli as well as spontaneous curvatures are specific to these parameters.

PROPERTIES OF MEMBRANE BILAYERS AND TUBES

We are primarily interested in the mechanisms by which membranes rearrange during fission and fusion, however, it is useful to elucidate the equilibrium properties of membranes and NTs. The stability of membranes as well as the tension in the membrane can be controlled by the chemical potential of the lipids, μ. Membranes are stable at high μ, but at low μ they dissociate into a homogeneous mixed state containing a small concentration of lipids. We first locate the membrane-homogeneous transition and the relationship between the free energy per unit area, tension, in the membrane and μ. We then calculate the free energy of an NT as well as its equilibrium radius and bending energy.

The free energy of a planar membrane is shown in Figure S3. The transition occurs at $\mu_{\text{MH}} = -11.3512\sqrt{\bar{N}}k_BT$. Expanding the free energy per unit area, $\sigma \equiv F/A$, to linear order in distance from the transition, $(\mu_{\text{MH}} - \mu)$, we find $\sigma \approx 1.374(\mu_{\text{MH}} - \mu)/R_0^2$ from Figure S3. The width of the bilayer at $\mu = \mu_{\text{MH}}$, given by the distance over over which the total lipid concentration is greater than 0.5, is $d = 1.22R_0$. σ and d, along with κ discussed below, are used to scale quantities in the main paper.

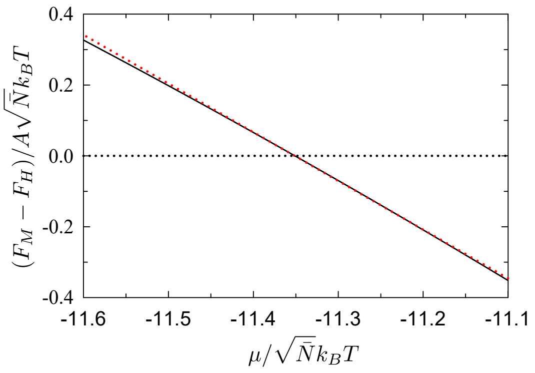

Figure S3: The free energy per unit area of a planar bilayer membrane is shown as a function of lipid chemical potential, μ. The red dotted line shows $F/A = 1.374(\mu_{\text{MH}} - \mu)/R_0^2$.

We are primarily interested in NTs and thus turn our attention thereto. There is a number of ways of calculating the bending energy of a membrane, such as that described in Ref. 1. SCFT gives us direct access to the free energy, making our task simpler than that of simulations. The first step is to find the radius of an NT from our SCFT calculations.

We define the radius of an NT as

$$r_{\rm NT} \equiv \frac{\int d\mathbf{r}\ \phi_\ell(\mathbf{r}) r^2}{\int d\mathbf{r}\ \phi_\ell(\mathbf{r}) r} \tag{1}$$

where $\phi_\ell(\mathbf{r})$ is the concentration of lipids at position $\mathbf{r}$ and $r = 0$ defines the cylindrical axis. We can then study the membrane bending energy which can be written as (2–4)

$$U_{\rm bend} = \int \left[\frac{\kappa}{2}(K - C_0)^2 + \bar{\kappa} K_G\right] dA \tag{2}$$

where κ and $\bar{\kappa}$ are the bending modulus and the saddle splay modulus, respectively. The extrinsic curvature, $K = 1/R_1 + 1/R_2$, is the sum of the two principal curvatures and the Gaussian curvature, $K_G = 1/(R_1 R_2)$, is their product. The inverse length, C_0, is the spontaneous curvature. In the case of a cylinder there is only one curvature, thus $K = 1/r_{\rm NT}$ and $K_G = 0$, and it follows from the symmetry of the membrane that $C_0 = 0$. Taking the integral in Equation 2 over the area of the cylinder and adding in the, previously discussed, free energy per unit area allows us to write the free energy of a cylindrical membrane, $F_{\rm NT}$, per unit length, L, as

$$\frac{F_{\rm NT}}{2\pi L} = r_{\rm NT}\sigma + \frac{\kappa}{2r_{\rm NT}} \tag{3}$$

where the first term is simply the free energy per unit area multiplied by the circumference of the cylinder and the second term is the bending energy.

Extracting σ from Figure S3, we then fit Equation 3 to the free energy in Figure S4A to obtain the bending modulus, $\kappa = 0.209\sqrt{\bar{N}}k_BT$. Minimizing the free energy, Equation 3, with respect to the radius results in $r_{\rm NT} = \sqrt{\kappa/2\sigma}$. Figure S4B shows a comparison with the radius found from SCFT.

Repeating this argument for a spherical vesicle of radius $r_{\rm V}$, the free energy then becomes

$$\frac{F_{\rm V}}{4\pi} = r_{\rm V}^2\sigma + 2\kappa + \bar{\kappa} \tag{4}$$

Unlike a cylinder, a spherical vesicle cannot minimize this free energy with any finite radius. If $\sigma < 0$, the vesicle can always decrease its free energy by growing, as with a cylinder, however if $\sigma > 0$ the vesicle shrinks unbounded. This is obviously a result of truncating the bending energy expansion, as higher powers of $1/r_{\rm V}$ would become more significant as $r_{\rm V}$ decreases. Nonetheless, we still have a simple way of extracting the saddle splay modulus: we employ SCFT calculations similar to the ones above, but with spherical symmetry. Using $\mu \gtrsim \mu_{\rm MH}$, so $\sigma \lesssim 0$, the vesicle 'tries' to grow but is repelled by the reflecting upper boundary. This procedure produces a small correction to the $r_{\rm V}^2$ term caused by the aforementioned repulsion, however the constant term, $2\kappa + \bar{\kappa}$, is unaffected, allowing us to extract $\bar{\kappa} = -0.07459\sqrt{\bar{N}}$. An example plot is shown in Figure S5.

Turning our attention back to cylinders, concentration profiles for single- and double-nanotubes, sNTs and dNTs, are

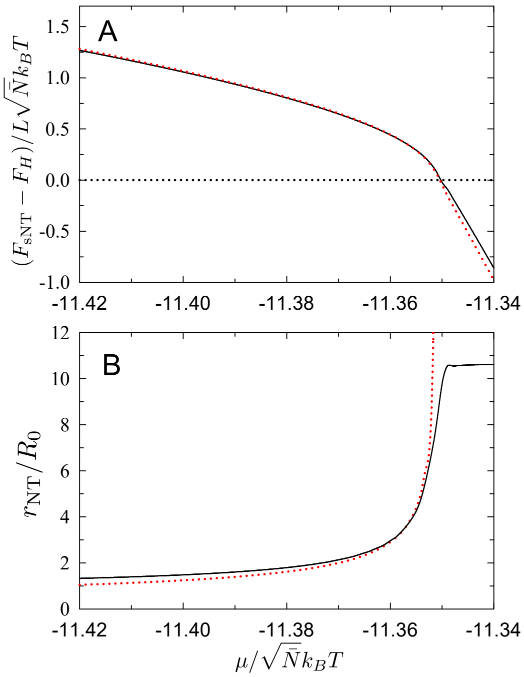

Figure S4: A Free energy of NTs per unit length and B equilibrium radii of NTs as a function of lipid chemical potential, μ. Red, dotted curves show fits to A $F/2\pi L = r_{\rm NT}\sigma + \kappa/2r_{\rm NT}$ and B $r_{\rm NT} = \sqrt{\kappa/2\sigma}$. Close to and above $\mu = \mu_{\rm MH}$ (where σ is small or negative), the radius is limited by the size of the system, and exhibits a plateau.

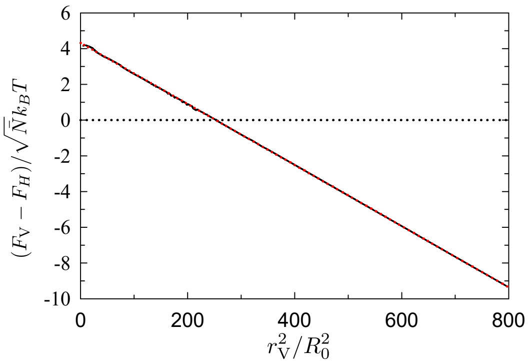

Figure S5: Free energy of a spherical vesicle as a function of radius, at $\mu = -11.35\sqrt{\bar{N}}k_BT$. The radius is controlled by a repulsion of the membrane against a reflecting boundary.

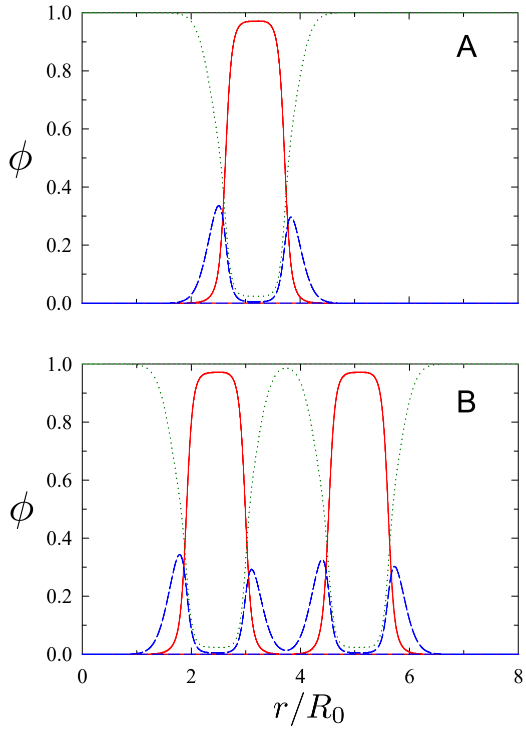

Figure S6: Profiles of A sNT and B dNT at $\mu = -11.373\sqrt{\bar{N}}k_BT$. Concentrations are shown of the (red) tail, (blue) head and (green) solvent as a function of the radial distance from the cylinder axis.

shown in Figure S6. At fixed chemical potential, NTs have a preferred radius, as discussed above. In a dNT, the radius of each is set by a combination of the preferred radius of each (that of a sNT) and the repulsion between head groups. The free energy of the dNT, relative to the mixed state, is slightly higher than double that of a sNT, due to a deviation from the equilibrium radius. Close to coexistence, however, this difference becomes small.

As we have seen, equilibrium membranes (above $\mu_{\rm MH}$) are planar (zero spontaneous curvature) and cylinders only form under tension. Throughout our calculations we are interested in the fission of NTs, as they exist in biological systems. In these cases, there are various influences, such as proteins and other cellular components, causing the membrane to retain its cylindrical shape. Close to $\mu_{\rm MH}$, we restrict the cylindrical radius simply by restricting the size of our box. This is similar to how we restricted the spherical vesicle and can also be seen in the plateau of the radius in Figure S4C, where the cylinder comes up against the reflecting boundary.

The results we have shown thus far were calculated from one-dimensional calculations, exploiting symmetries of the system. Several calculations in the main paper also use cylindrical symmetry, however, when this is not possible, as for the more complicated membrane rearrangements, we employ a cuboidal box. For the chemical potentials that we use, the

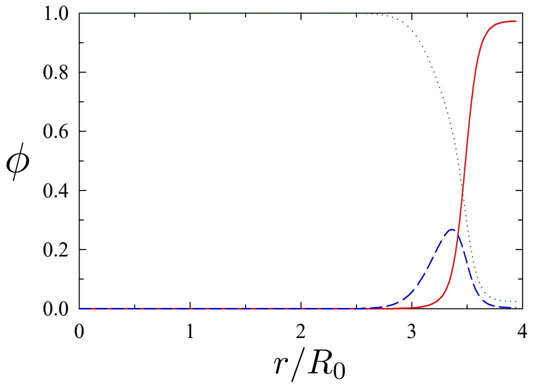

Figure S7: A planar monolayer profile is illustrated. Concentrations are shown of the (red) tail, (blue) head and (green) solvent as a function of position along the dimension normal to the membrane.

square cross-section of the box has little effect. This is confirmed by the following: (i) the circular symmetry of the NTs (where expected - small deviations are noted for low tensions), (ii) recalculations where the system was initialized in rotated configurations, and (iii) comparisons between the full 3D calculation (in a cuboidal box) and the 2D (cylindrically-symmetric) calculations, where applicable. At higher μ, the cylinder pushes against the box boundaries and begins to deform, but this deformation is small (if present at all) for the values of μ presented.

PROPERTIES OF MEMBRANE MONOLAYERS

It is also instructive to examine the properties of membrane monolayers. To do this, we construct systems with profiles like those in Figure S7. Reflecting boundaries are implemented on either end of the simulation. Similar calculations can be conducted in planar, cylindrical or spherical geometries in order to examine the properties of planar, cylindrical and spherical monolayers.

Unlike bilayers, monolayers are not symmetric, and we cannot say *a priori* that the spontaneous monolayer curvature is $C_{0,m} = 0$. For the case of a cylindrical monolayer, the free energy per unit length becomes

$$\frac{F_{{\rm NT},m}}{2\pi L} = r_{\rm NT}\tilde{\sigma}_m + \frac{\kappa_m r_{\rm NT}}{2}\left(\frac{1}{r_{\rm NT}} - C_{0,m}\right)^2 \quad (5)$$

$$= r_{\rm NT}\sigma_m + \frac{\kappa_m}{2r_{\rm NT}} - C_{0,m}\kappa_m \quad (6)$$

where $\tilde{\sigma}_m$ is the free energy per unit area of the monolayer, $\sigma_m = \tilde{\sigma}_m + \kappa_m C_{0,m}^2/2$ is the free energy per unit area of a planar monolayer and κ_m is the monolayer bending modulus. The free energy of a spherical monolayer with radius $r_{\rm V}$ is

$$\frac{F_{{\rm V},m}}{4\pi} = r_{\rm V}^2\tilde{\sigma}_m + \frac{\kappa_m r_{\rm V}^2}{2}\left(\frac{2}{r_{\rm V}} - C_{0,m}\right)^2 + \bar{\kappa}_m \quad (7)$$

$$= r_{\rm V}^2\sigma_m + 2\kappa_m - 2\kappa_m C_{0,m} r_{\rm V} + \bar{\kappa}_m \quad (8)$$

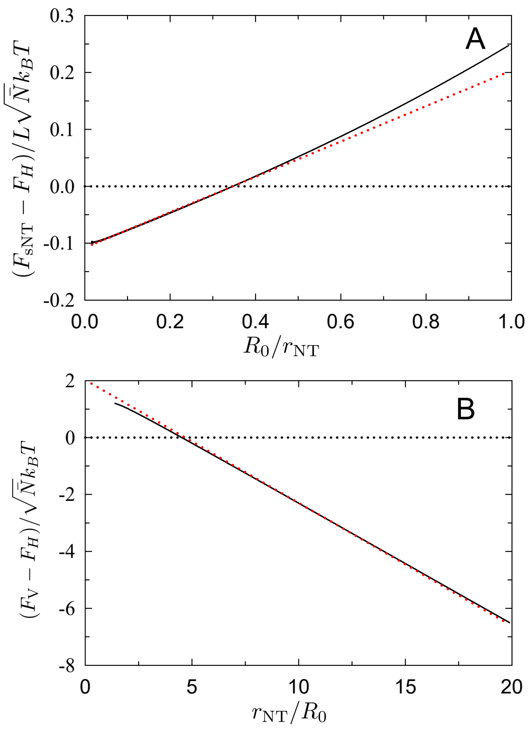

Figure S8: Free energies are shown for A cylindrical and B spherical monolayers as functions of radius, with $\mu = \mu_{\rm MH}$ chosen so that the free energy density of a planar membrane is $\sigma_m = 0$. Red curves illustrate fits to A Equation 6 and B Equation 8.

where $\bar{\kappa}_m$ is the monolayer saddle splay modulus.

Similar to our previous calculations, we examine the free energy of cylindrical and spherical monolayers. In this case, we control the radius of the cylindrical or spherical structures through our choice of the system radius. The resulting free energies are shown in Figure S8. Fitting the curves to Equation 6 and Equation 8 allows us to extract the monolayer bending and saddle splay moduli, $\kappa_m = 0.09909\sqrt{\bar{N}}k_BT$ and $\bar{\kappa}_m = -0.03741\sqrt{\bar{N}}k_BT$, and the spontaneous monolayer curvature, $C_0 = 0.1734R_0^{-1}$. As one might expect, the bending and saddle splay moduli for the monolayers are close to half of that for the bilayer.

The small deviation from $\kappa = 2\kappa_m$ likely occurs because we ignored the membrane thickness, ΔR. The bending energy of a bilayer is given by the bending energy of the two monolayers *i.e.* the sum of Equation 6 for the $r_{\rm NT}$ of the inner and outer membranes, with $C_{0,m}$ having opposite signs for the two membranes. For a large bilayer radius, the inner and outer radii are approximately the same and we recover Equation 3 with $\kappa = 2\kappa_m$, plus a small correction of order $\Delta R^2/r_{\rm NT}^3$. Although insignificant for large radii, this correction may account for the deviation from strictly $\kappa = 2\kappa_m$ since the fits from which we extracted κ and κ_m included small radii. A similar argument applies to $\bar{\kappa}$ and $\bar{\kappa}_m$.

LEAFLETS UNDER DIFFERENT TENSION

Throughout this work, we have assumed that all membranes (and membrane leaflets) are under the same tension, fixed by their chemical potential. When using constriction due to tension as a proxy for other, more complicated, ways of constricting the membrane, this approximation is likely appropriate, but should be both noted and tested. Testing the effects of tension differentials is particularly important in systems such as mitochondria, where the inner and outer membranes are attached to different reservoirs with, presumably, in general, different tensions. A thorough investigation is beyond the scope of this manuscript, but here we lay the groundwork for simulating membranes under different tensions using SCFT.

As described above, the tension in a membrane (or leaflet) is set by the lipid chemical potential. Furthermore, the instantaneous exchange of lipids between leaflets implies that membranes in the same simulation (with a fixed lipid chemical potential) will have the same tension. Even if we introduce multiple (miscible) lipid species, with separate chemical potentials, they will instantly mix. The solution is to introduce a repulsion between the head groups of otherwise identical lipid species, thereby relegating lipids to a specific leaflet, while allowing their tail groups to mix. This was done previously in (5). Example membranes with the same and different tensions in each leaflet are shown in Figure S9. The difference in tension is visible in the asymmetry in the head-group density on either side of the membrane.

We utilize a head-head repulsion of $\chi N = 300$ in order to reduce overlap to almost zero. Note that this large χN value does not bring about the significant challenges usually seen in SCFT with large χN because there are no interfaces between repulsive heads.

It is also important to note that at a fixed chemical potential and finite head-head repulsion, there is a shift in the free energy and thus zero-tension chemical potential which, though small, is noticeable on the scale of our calculations. For $\chi N = 300$ the shift in $\mu_{\rm MH}$ is approximately $9.9 \times 10^{-3}\sqrt{\bar{N}}k_BT$, thus the μ values in the plot below are this amount lower than the quantities above to obtain the same tension. The shift in the free energy is due to the small number of extra lipids allowed in the system due to the finite amount of mixing, and disappears as the head-head repulsion diverges.

The next steps are to repeat the sNT calculations using a different tension for the inner and outer leaflets. Preliminary calculations (not shown) suggest that the average tension dictates the radius of the composite tube, thus we anticipate only a small effect on the barrier. One can also repeat the dNT calculations using combinations of leaflets in a membrane to create the desired membrane tension. Note that this must be done carefully. If the above technique is used and apposing leaflets are of different species, then the extra repulsion may far exceed the usual hydration repulsion, and would likely preclude fusion. This investigation is left for future work.

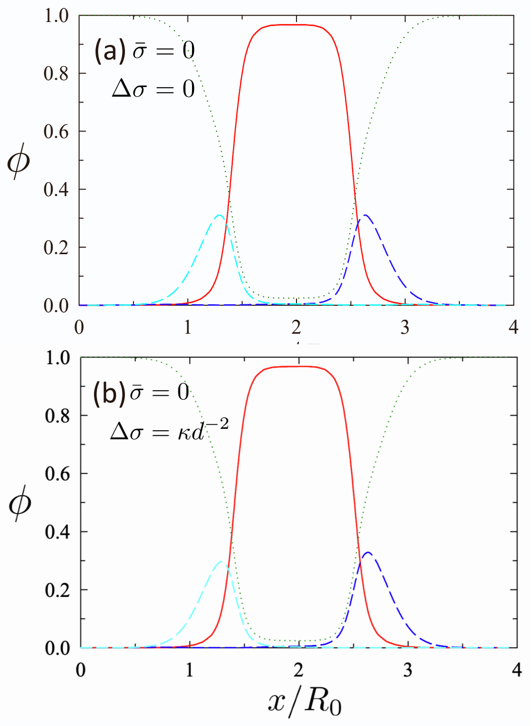

Figure S9: Profiles of planar bilayers, similar to Figure S6 and Figure S7, calculated at (a) zero tension, and (b) a total tension of zero, but the two leaflet having tensions of $\Delta\sigma = \pm\kappa/d^2$. Different head types are shown in blue and cyan.

LIPID DIFFUSION IN PLANAR MEMBRANES

In order to extract dynamical information from our particle-based simulations, we conduct simulations of planar membranes in order to compare the in-membrane self-diffusion constant of lipids to that found in experiments. An illustration of such a planar membrane and a plot of average lipid displacement is shown in Figure S10. From this we can extract a diffusion constant of approximately $D = 1.9 \cdot 10^{-4}\text{nm}^2/\Delta t$. Approximating the in-plane diffusion constant of lipids as $4\mu\text{m}^2/\text{s}$ (6) allows us to estimate a SOMA time step size of $\Delta t = 2 \cdot 10^{-10}s$. Other particle-based simulation methods, such as MARTINI (7), use time steps of $10^{-14}s$, making this a significant increase in speed. The uncertainties in both the membrane thickness (particularly between various cellular components) and lipid diffusion time are quite high, particularly as both quantities vary significantly depending on the particular membrane under consideration and the membrane environment. The resulting estimate for our time step is thus only a rough approximation. Based on the variation in the aforementioned literature values, we expect our estimate to be within a factor of 4 of the true value.

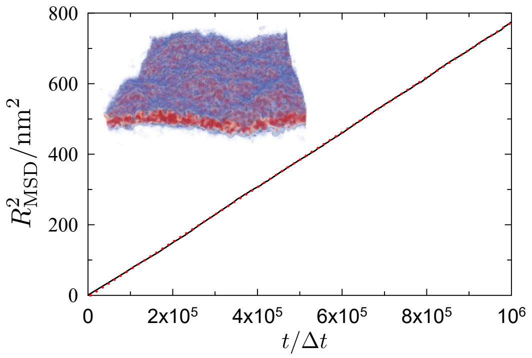

Figure S10: Mean-squared lipid displacement in the membrane plane as a function of time step, for a planar membrane in SOMA. The scaling of the vertical axis was calculated by equating an average membrane bilayer thickness of 4nm (8) with the thickness found in SCFT (see Figure S6) of approximately $1.22R_0$. The inset shows an image of the planar bilayer used in SOMA. Fitting the mean-squared displacement, ΔR^2_{MSD}, to $R^2_{\text{MSD}} = 4Dt$ yields $D = 1.9 \cdot 10^{-4}\text{nm}^2/\Delta t$, where Δt is a SOMA time step.

MEMBRANE TUBE COLLAPSE RATE

Unlike the SCFT calculations, the coarse-grained simulations can give us an order-of-magnitude estimate for the time taken to undergo the aforementioned transformation processes. Using the time step calculated above, $\Delta t = 2 \cdot 10^{-10}$ s, allows us to investigate the time taken to rearrange membranes.

The collapse of an NT into a WLM is shown in Figure S11. Approximating the membrane width as 4 nm (8) and using the previously-calculated time step, we estimate that the collapse of the NT proceeds at a rate of approximately $100\mu\text{m/s}$. At higher lipid concentration (or chemical potential), the NT has a larger diameter and collapses more slowly and the converse is also true. Repeating the above calculation, the rate of collapse appears to be proportional to the free-energy difference between sNT and WLM, which varies linearly with tension. Using the fact that $R_c = \sqrt{\kappa/2\sigma}$, we can write the speed of WLM elongation as $v = C\left(R_c^{-2} - R_{cW}^{-2}\right)$, where R_{cW} is the radius of a membrane tube at sNT-WLM coexistence. For the case outlined above, $C \approx 1.973 \cdot 10^6\text{nm}^3/\text{s}$ and $R_{cW} \approx 8\text{nm}$.

The illustrated NT is under high tension, chosen to intentionally increase the speed due to the slow speed of simulations.[1] If we were to decrease the tension so as to adjust the collapse rates in our simulations, the simulations would take prohibitively long. It should also be noted that we are approximating lipids as polymers and polymer membranes can withstand much higher tension and areal strain before rupture (9, 10) and the illustrated system is likely beyond the tension where lipid membranes would be stable.

[1]The initial condition was calculated from an NT at $\mu - \mu_{\text{MH}} = -0.26\kappa$, where the radius of the tube is $R_c \approx 3.9\text{nm}$.

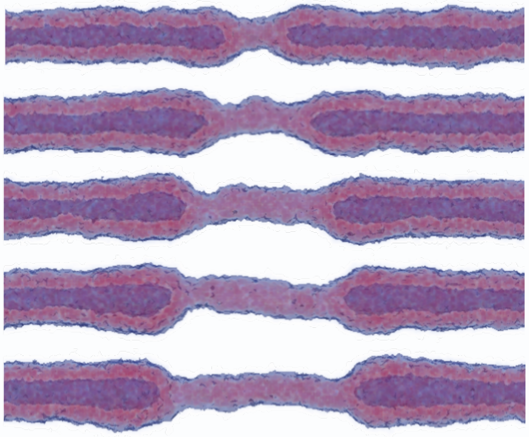

Figure S11: The collapse of a sNT into a WLM in a SOMA simulation is shown at intervals of $2 \cdot 10^5 \Delta t$. Approximating the membrane thickness as 4nm (8), the collapse proceeds at approximately $2 \cdot 10^{-5} \text{nm}/\Delta t$.

TRANSITION PATHWAY DETAILS

In order to clarify the pathways presented in the main text, this section reproduces, annotates and expands upon Figures 5-8 and discusses variations to the mechanisms presented in the main text. In cases where membrane changes may not be obvious, we have highlighted the relevant locations on the images.

We first consider a variation of the sNT collapse mechanism. As discussed in the main text, the minimum free energy path (MFEP) connecting an intact sNT and a pair of capped sNTs does not include pores. Seeing as short-lived pores are apparently readily nucleated by interactions between membranes in a dNT, we wished to investigate the fission mechanism of a sNT where we enforce that the initial condition contains a pore. This is illustrated in Figure S12. rather than closing the pore, and then undergoing fission in the usual way (collapse to HF, then WLM, then fission of the WLM) the pore facilitates fission by simultaneously unzipping the sNT and closing it, until there are two capped sNTs connected by a WLM, with pores in the caps, which then close.

Note that the starting state of Figure S12 necessarily involves an external field that renders the pore in the sNT metastable. Without such an external field, a pore in the sNT is *not* metastable, *i.e.* if allowed to relax, it would close and an intact sNT would form. A pore in a sNT could be rendered metastable, *e.g.* by proteins. In this case, the free-energy barrier between the sNT with a pore and the WLM is lower than that associated with a collapse of an intact sNT.

Figure S13 reproduces Figure 6A in the main text, and represents the simplest pathway for merging the inner and outer membranes in a dNT. Starting from A the unperturbed dNT, the outer membrane bends inwards towards the inner membrane (highlighted in B). A small contraction of the inner NT is also visible. C A stalk then grows between the inner and outer membranes. The membrane thins on either side of the stalk - this is first apparent in D. E-F The stalk widens horizontally and the connection thins (thins vertically, from the vantage point of the lower panels). This produces a thin hemifusion diaphragm (HD), which separates the inner and outer regions. G-H The HD may then broaden. The free-energy barrier to rupturing this HD is small, and we have observe it rupturing, to form a pore, in some particle-based simulations.

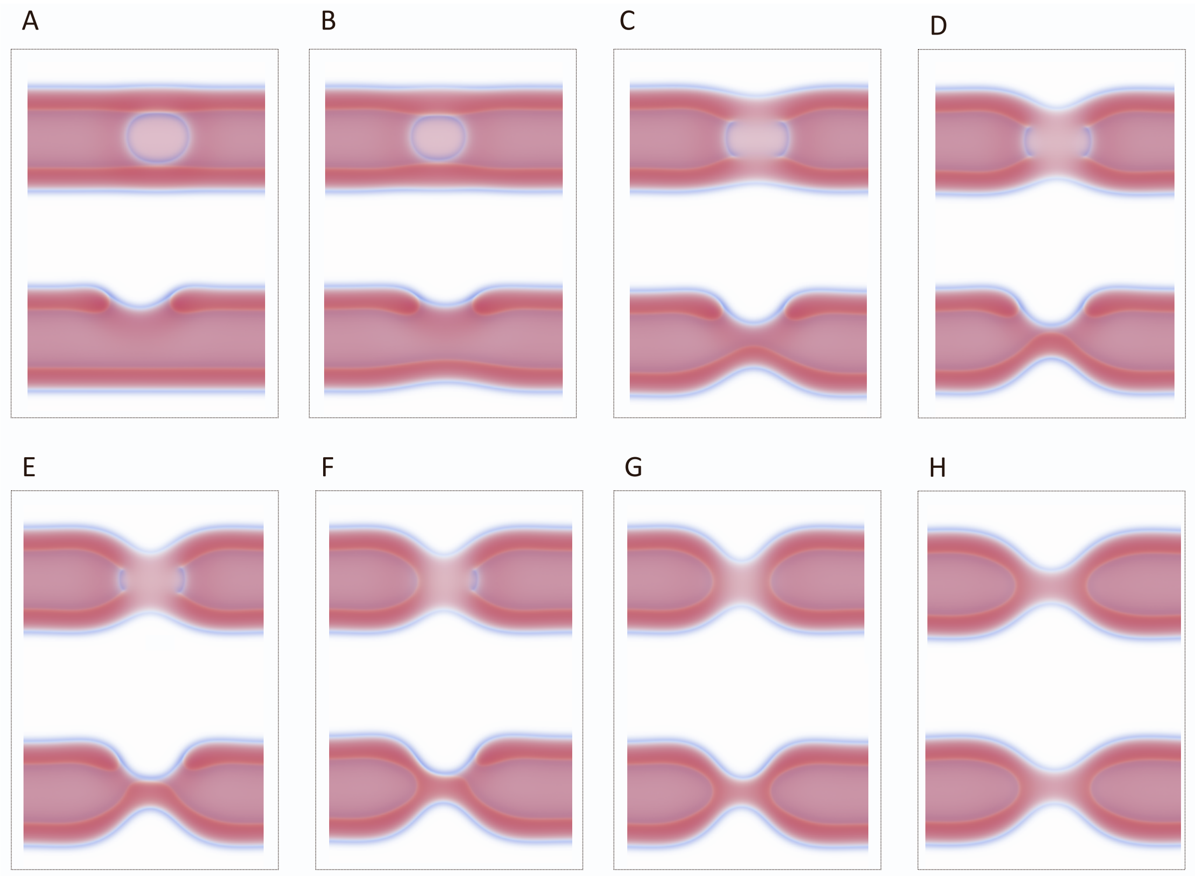

Figure S12: Images of the fission of a sNT starting from a state containing a pore at $\sigma = 0.387\kappa/d^2$.

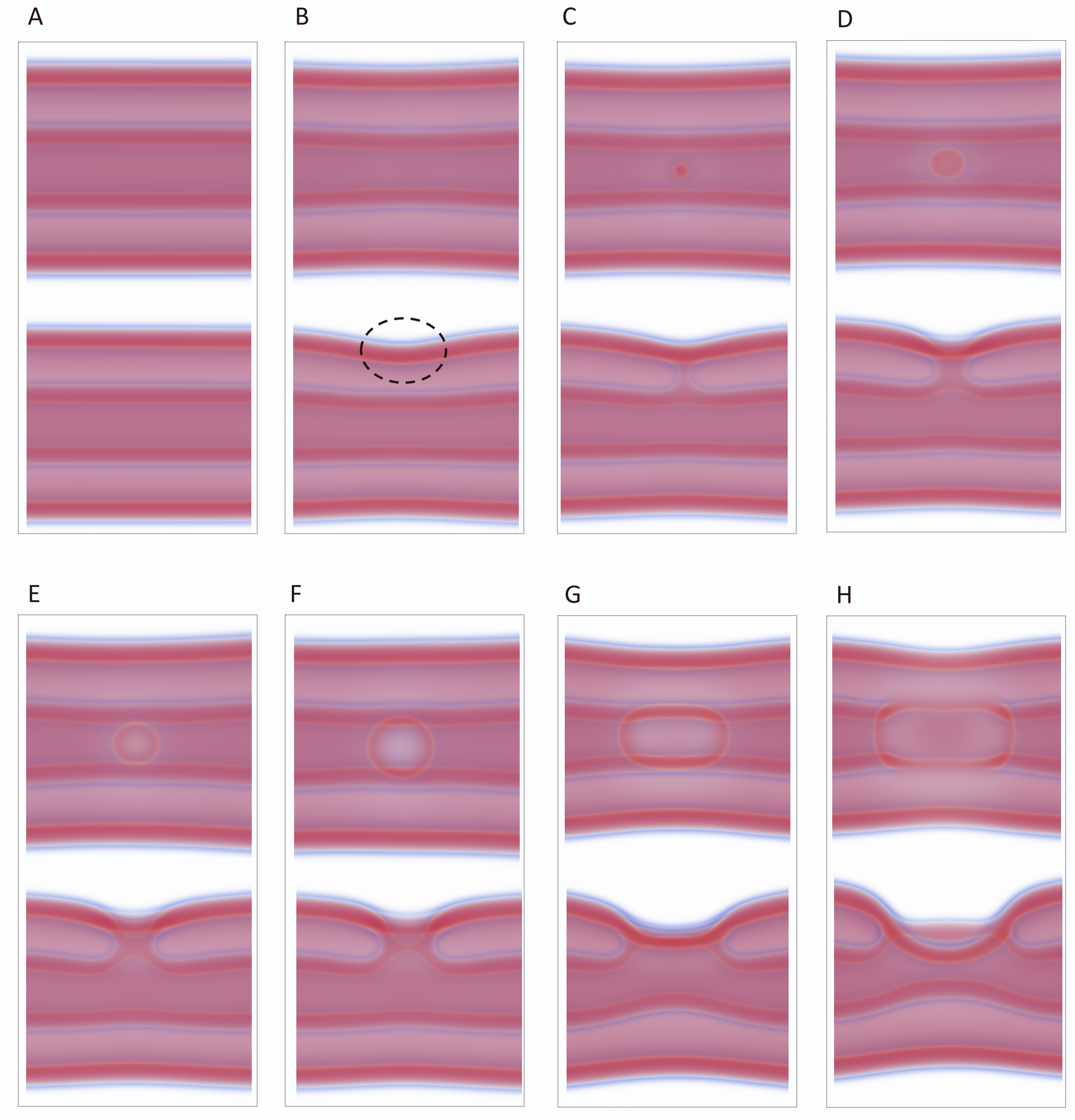

Figure S13: Enlarged versions of the images in Figure 4A in the main text. Panel A has been added and represents the unperturbed double membrane. The change in panel B has been highlighted. Unlike in the main text, outer tubes have not been made transparent.

Figure S14 reproduces Figure 6B in the main text, and represents the variation on the previous pathway, where a pore intermediate is present in the outer membrane. The transition begins similarly, bending the outer membrane towards the inner and forming a stalk. Once again, the membranes become thinner around the stalk. This time, however, a pore nucleates in D. The stalk widens when the pore forms (going from panel C to D). The stalk continues to widen, and zips around the pore. The expanding connection is highlighted in E. The next three steps, F-H, are the same as in the previous pathway.

In fact, there are more variations of this pathway. Figure S15 shows a path where a stalk forms (as in Figure S14) and nucleates a pore in the inner membrane. As before, the stalk then grows around the pore. Another variant of this, shown in Figure S16, is to first form a pore, which then causes a stalk to form next to it and subsequently zip around the pore. A similar mechanism (not shown) can occur, involving a pore first forming in the outer membrane. Other variations may involve multiple stalks, for example on either side of the pore, stalks forming at different positions *i*.e. not adjacent along the axis of the cylinder, but at different angles etc.

The barrier to each of these pathways is similar – the difference is negligible on the scale of our calculations. The difference in free-energy barrier between these pathways is so similar because of the following: the barrier (free energy difference between the peak and preceding minimum) is determined by the highest peak compared with the unperturbed dNT state. The barrier to reversing the transition (see discussion in the main text) is small along any of these paths, which is to say, the barrier height is dominated by the difference in free energy between the hemifused state and the unperturbed dNT state.

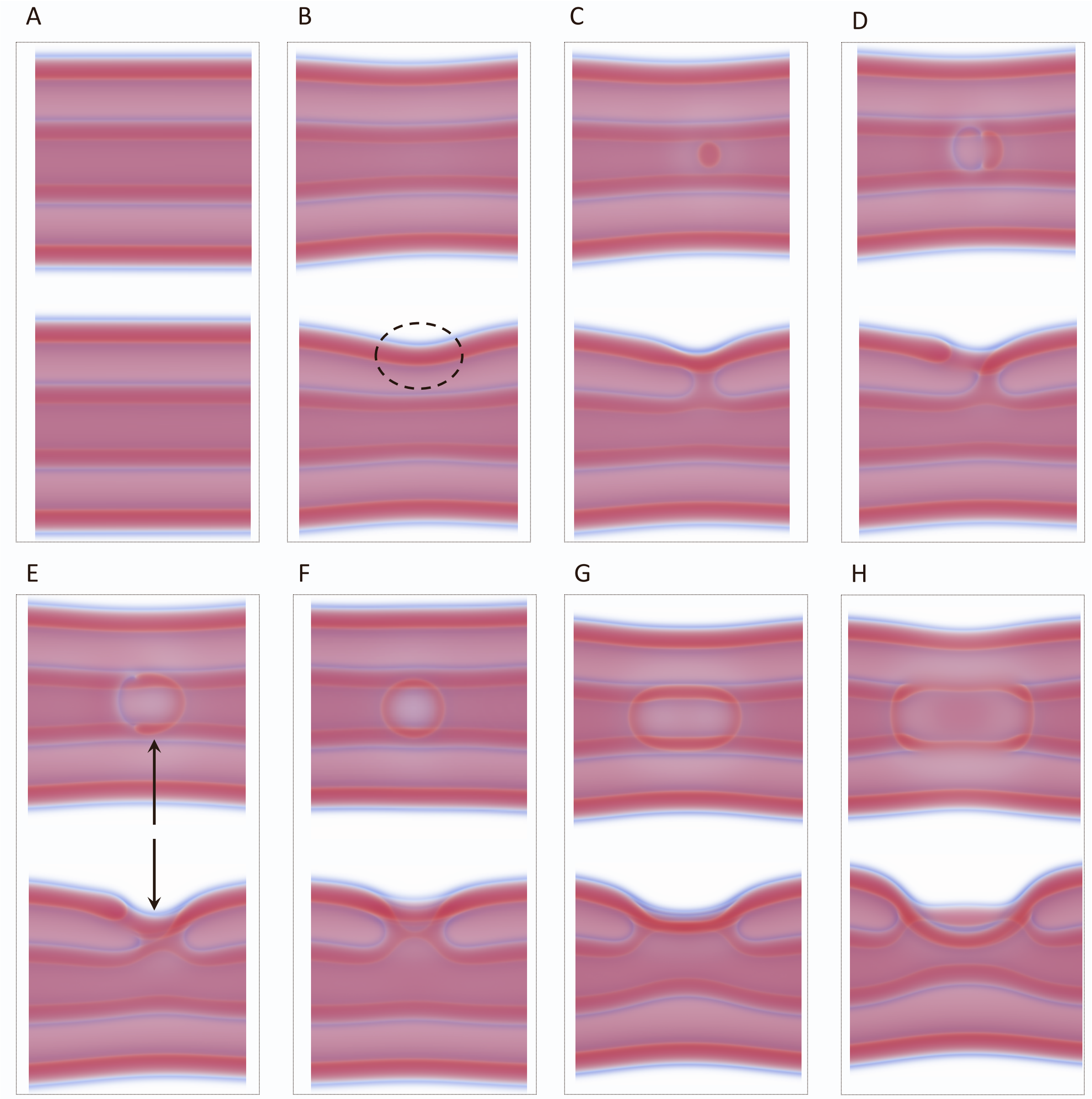

Figure S14: Enlarged versions of the images in Figure 4B in the main text. Panel A has been added and represents the unperturbed double membrane. Interesting points in panels B and E have been highlighted.

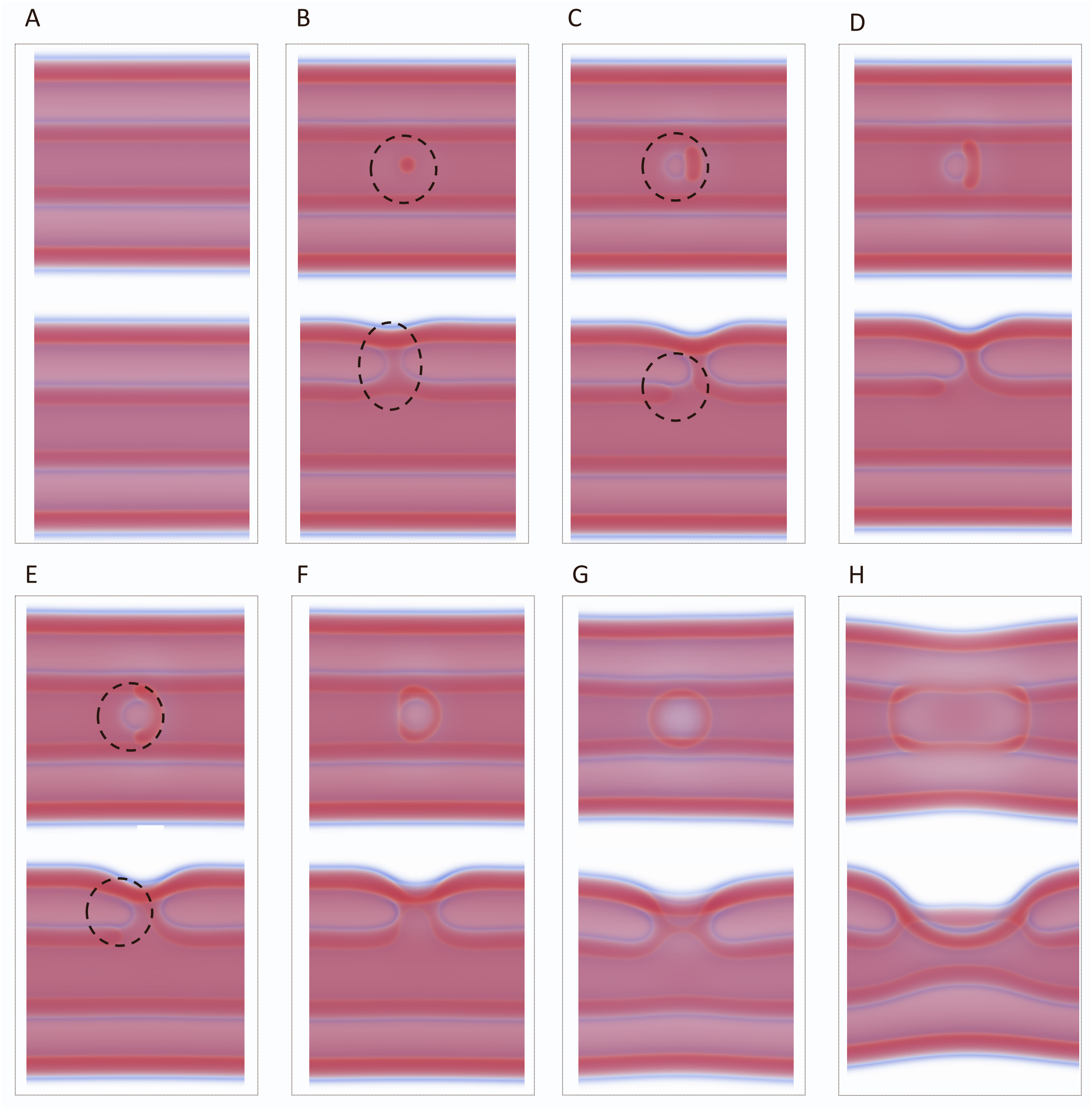

Figure S15: A path similar to Figure S14 but with a pore forming on the inner membrane, at $\sigma = 0.387\kappa/d^2$.

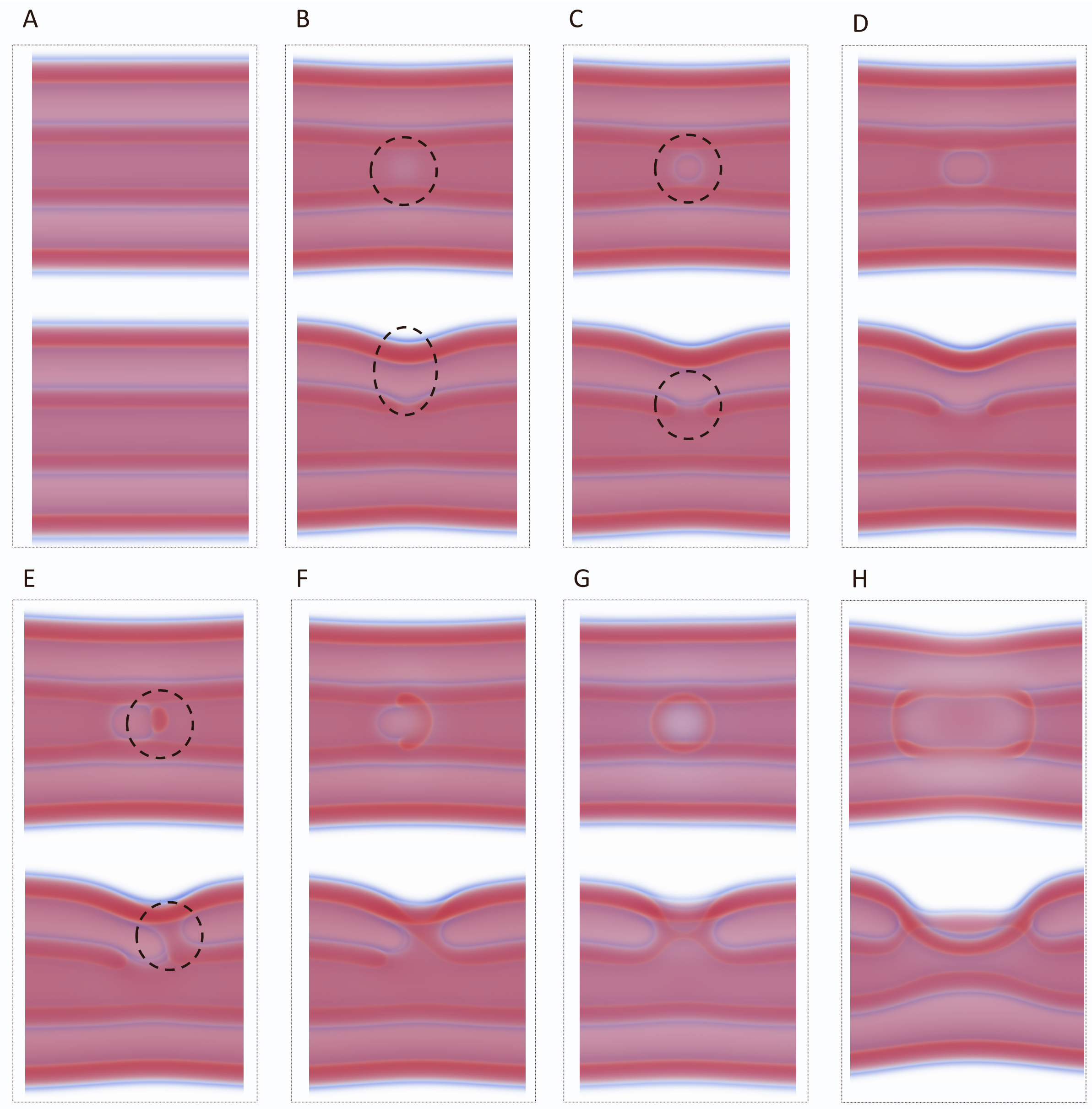

Figure S16: A path similar to Figure S15 but with the pore forming before the stalk, at $\sigma = 0.387\kappa/d^2$.

Figure S17 reproduces Figure 6D in the main text for $\sigma = 0.387\kappa/d^2$, with images added to illustrate the collapse of the inner NT and the concomitant formation of a WLM. The initial state, panel A, is the unperturbed dNT. Both NTs become thinner in panel B. As the inner NT thins, on its way to a WLM, the hydrostatic repulsion pushing on the outer NT decreases, allowing it to partially adopt a thickness closer to its equilibrium radius. In panel C the inner NT collapses, connecting to itself. The connection grows in panel D, forming a long WLM that connects two capped NTs. The growth continues and, in panel F, the outer membrane bends inward towards the WLM.

In the next phases of the evolution, depicted in panel G, a stalk forms at the saddle-point of the inward-bending outer NT. Note that the image in panel F has been rotated 90° (about the cylinder axis) relative to panel G, in order to illustrate the inward bend. The orientation is consistent in all other images. Just 'above' and 'right' of the stalk in panel G, there is another inward bend from the outer NT, and the WLM bends slightly 'right' towards the outer membrane. A second stalk forms here, as shown in panel H. In panel I this is followed by a pore forming in the inner NT. This pore connects the inner and intermediate regions, making this pathway 'leaky'.

After the stalks and pore have formed, panel J, the 'left' stalk widens, followed by the right stalk widening in panel K. The two wide stalks merge at the 'top' and then at the 'bottom', in panels L and M, respectively. The inside of the 'upper' inner NT is now once again disconnected from the intermediate region. Note that in panel M the 'lower' portion of the WLM remains a WLM. Panel H shows that this WLM shortens and widens, similar to what we saw in Figure S13, forming a diaphragm, which separates the 'upper' and 'lower' inner NTs. The diaphragm then ruptures in panel O, reconnecting the inner NTs and adopting the same final state seen in Figure S13 and Figure S14. At lower tension (higher μ) the hemifusion diaphragm is more circular. The preferred shape appears to be controlled by the curvature of the membranes.

As with the previous pathway, variations of this mechanism exist. Some features are consistent between all pathways that we have observed, *e.g.* the presence of two stalks.[2] The steps L-O, however, may occur differently, with a pore first opening in the 'lower' NT (similar to the pore in I) and the connections growing around this pore (similar to the steps K-L).

Figure S18 reproduces Figure 7 in the main text for $\sigma = 0.387\kappa/d^2$. The HD, shown in the final panel of Figure S13, expands around the dNT. As the HD expands, the highly-curved region highlighted in B becomes thin. This is apparent from the light color. C As the curvature increases further, a pore opens in the outer membrane and D expands. The pore closes by connecting the outer membrane to the inner membrane. This re-connection is beginning to form in E. The result is two dNTs connected by an sNT, the cylindrically hemifused state. As with the other pathways, a variation exists of this pathway, where a pore opens in the outer membrane earlier, *i.e.* close to the time represented by B. The pore then travels around the membrane until C. Once again, we showed only the variation with the lowest free energy.

Figure S19 reproduces Figure 9A in the main text. Once again, the main changes are highlighted. Starting from the cylindrically hemifused state (final state in Figure S18) B a pore forms in the inner membrane. The pore begins to 'unzip' the inner and outer membranes. As it unzips, the 'open' side of the inner NT bends inward and connects to the other side, closing the inner NT. D What remains is an inner NT, connected to the outer NT by a stalk. E The stalk ruptures.

Figure S20 reproduces Figure 9b in the main text. This time, rather than forming a pore first, the inner NT connects to itself forming an HD separating the inner region. This is highlighted in B. The formation of this HD is likely helped by the inward bend seen at the top of A. C The HD extends into a stalk, or WLM. The inner NT is now a capped NT, connected to an HD by a WLM. D The WLM ruptures, leaving the HD intact. E The HD ruptures shortly thereafter.

Figure S21 reproduces Figure 9c in the main text. A The outer membranes come closer to one another (right hand side) and B a pore forms between them. As we have seen before, the pore expands as shown in B-D, leaving only a WLM. The WLM then ruptures. There is some variation in the geometry of this mechanism (though not the topology) depending on the tension in the NT. At high tension, the sNT in the middle is longer than at low tension (See Figure 9 in the main text).

In the case of a long sNT, the outer membranes do not bend towards one another, rather a pore opens on one side of the sNT (*i.e.* close to a connection point). Once the WLM forms, it ruptures at a connection point and retracts, similar to what we saw for the fission of a WLM connecting two capped NTs. At low tension, where the sNT is short, the WLM is merely a short stalk and ruptures in the middle.

In addition to the pathways illustrated in the main text, the CH state may collapse through via the fission of the ST, as illustrated in Figure S22. The ST collapses via the canonical pathway and then retracts. Once disconnected, this state continuously transforms into two capped tubes, connected by a stalk. We expect that in experimental systems, the double tubes would retract further before this occurs, thus the barrier should be that of the canonical pathway and involve the retraction of a capped single tube. This is not shown due to the small size of our simulations box.

[2]As described in the main text, if one stalk is manually removed from the string, it reforms when the string is relaxed.

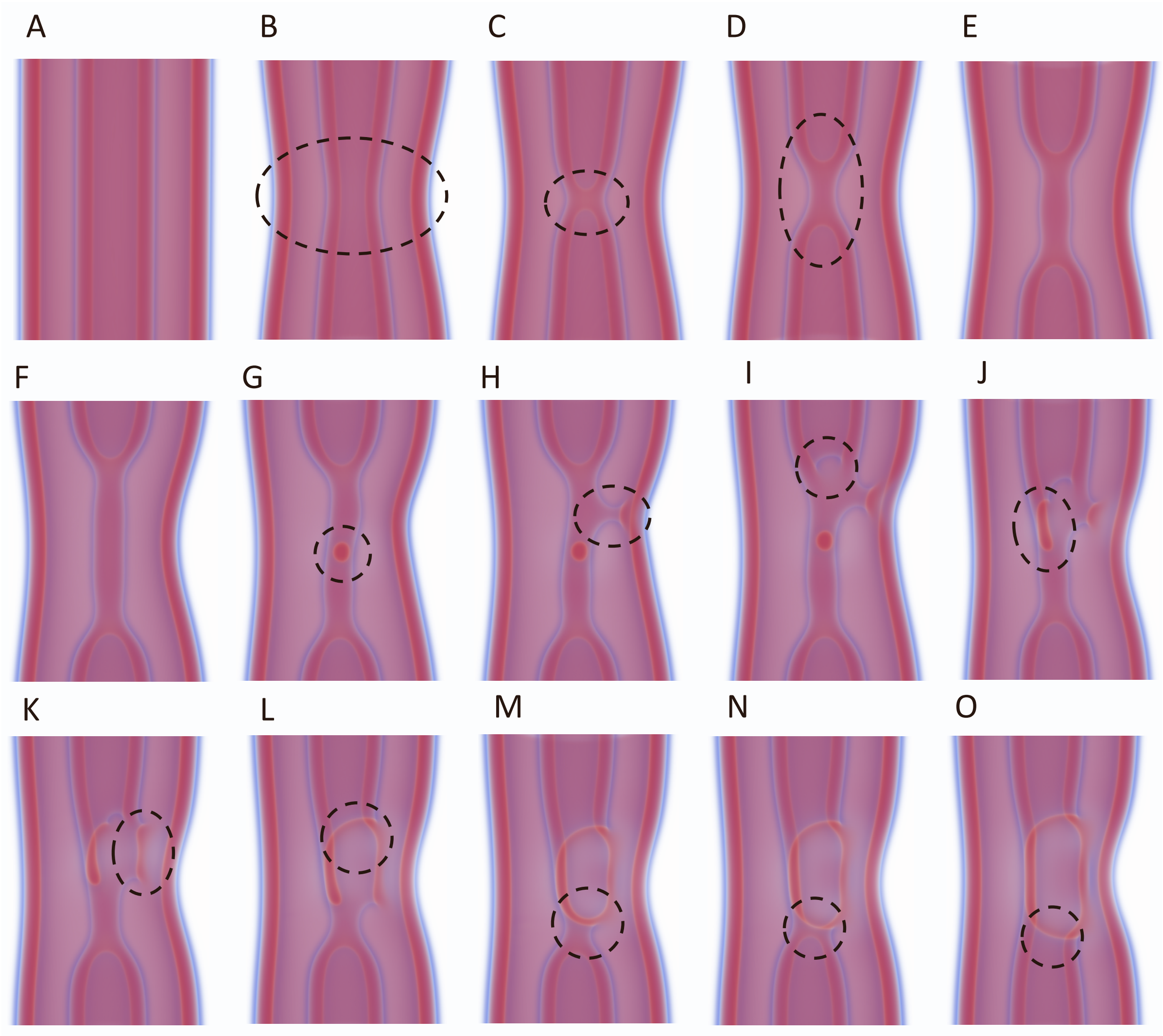

Figure S17: Enlarged versions of the images in Figure 4D in the main text. Panels A-E have been added and represent the collapse of the inner NT into a WLM. Interesting points have been highlighted to aid visualization and discussion.

REFERENCES

1. Harmandaris, V. A., and M. Deserno, 2006. A novel method for measuring the bending rigidity of model lipid membranes by simulating tethers. *Journal of Chemical Physics* 125:204905.

2. Helfrich, W., 1973. Elastic Properties of Lipid Bilayers: Theory and Possible Experiments. *Zeitschrift für Naturforschung C* 28:693–703.

3. Evans, E., 1974. Bending Resistance and Chemically Induced Moments in Membrane Bilayers. *Biophysical Journal* 14:923–931.

4. Canham, P., 1970. The minimum energy of bending as a possible explanation of the biconcave shape of the human red blood cell. *Journal of Theoretical Biology* 26:61–81.

5. Spencer, R. K., Y. G. Smirnova, A. Soleimani, and M. Müller, 2024. Transient pores in hemifusion diaphragms. *Biophysical Journal* 123:2455–2475.

6. Macháň, R., and M. Hof, 2010. Lipid diffusion in planar membranes investigated by fluorescence correlation spectroscopy. *Biochimica et Biophysica Acta (BBA) - Biomembranes* 1798:1377–1391.

7. Souza, P. C. T., R. Alessandri, J. Barnoud, S. Thallmair, I. Faustino, F. Grünewald, I. Patmanidis, H. Abdizadeh, B. M. H. Bruininks, T. A. Wassenaar, P. C. Kroon, J. Melcr, V. Nieto, V. Corradi, H. M. Khan, J. Domański, M. Javanainen, H. Martinez-Seara, N. Reuter, R. B. Best, I. Vattulainen, L. Monticelli, X. Periole, D. P. Tieleman, A. H. de Vries, and S. J. Marrink, 2021. Martini 3: a general purpose force field for coarse-grained molecular dynamics. *Nat. Methods* 18:382–388.

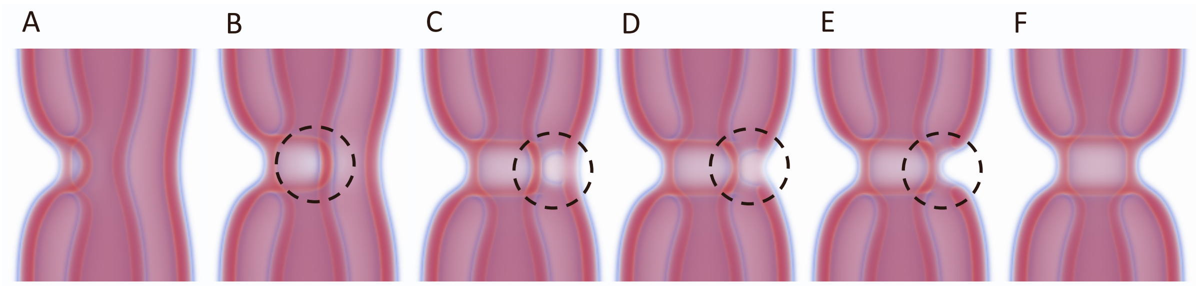

Figure S18: Enlarged versions of the images in Figure 7 in the main text. Interesting points have been highlighted to aid visualization and discussion.

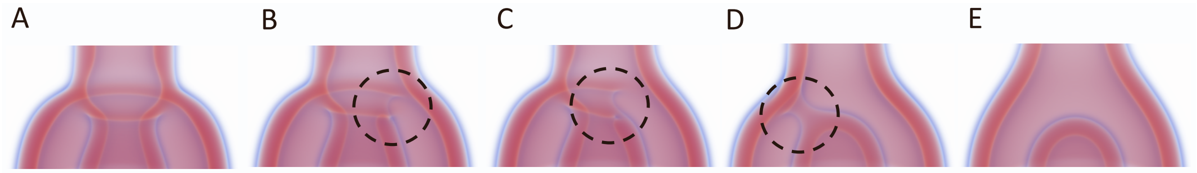

Figure S19: Enlarged versions of the images in Figure 9a in the main text. Interesting points have been highlighted to aid visualization and discussion.

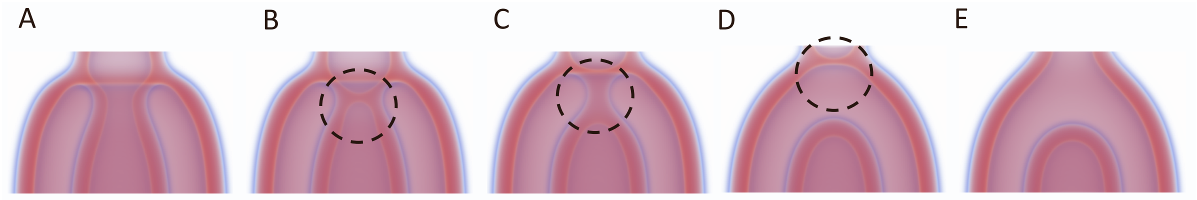

Figure S20: Enlarged versions of the images in Figure 9b in the main text. Interesting points have been highlighted to aid visualization and discussion.

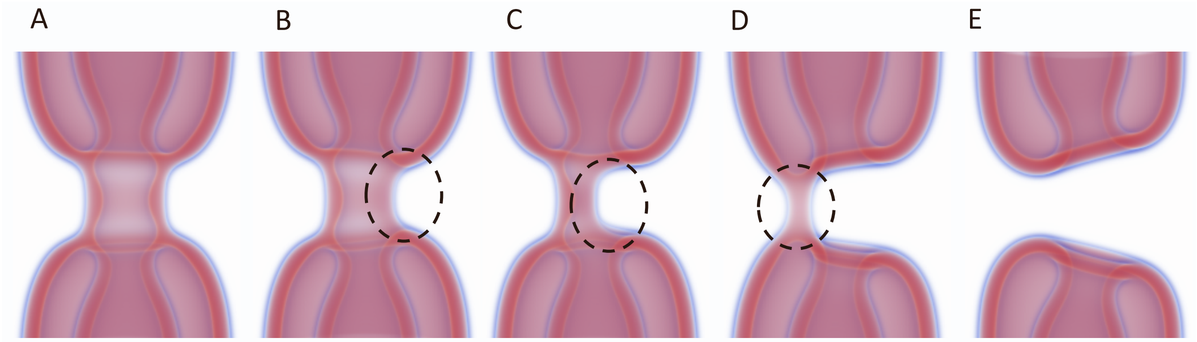

Figure S21: Enlarged versions of the images in Figure 9c in the main text. Interesting points have been highlighted to aid visualization and discussion.

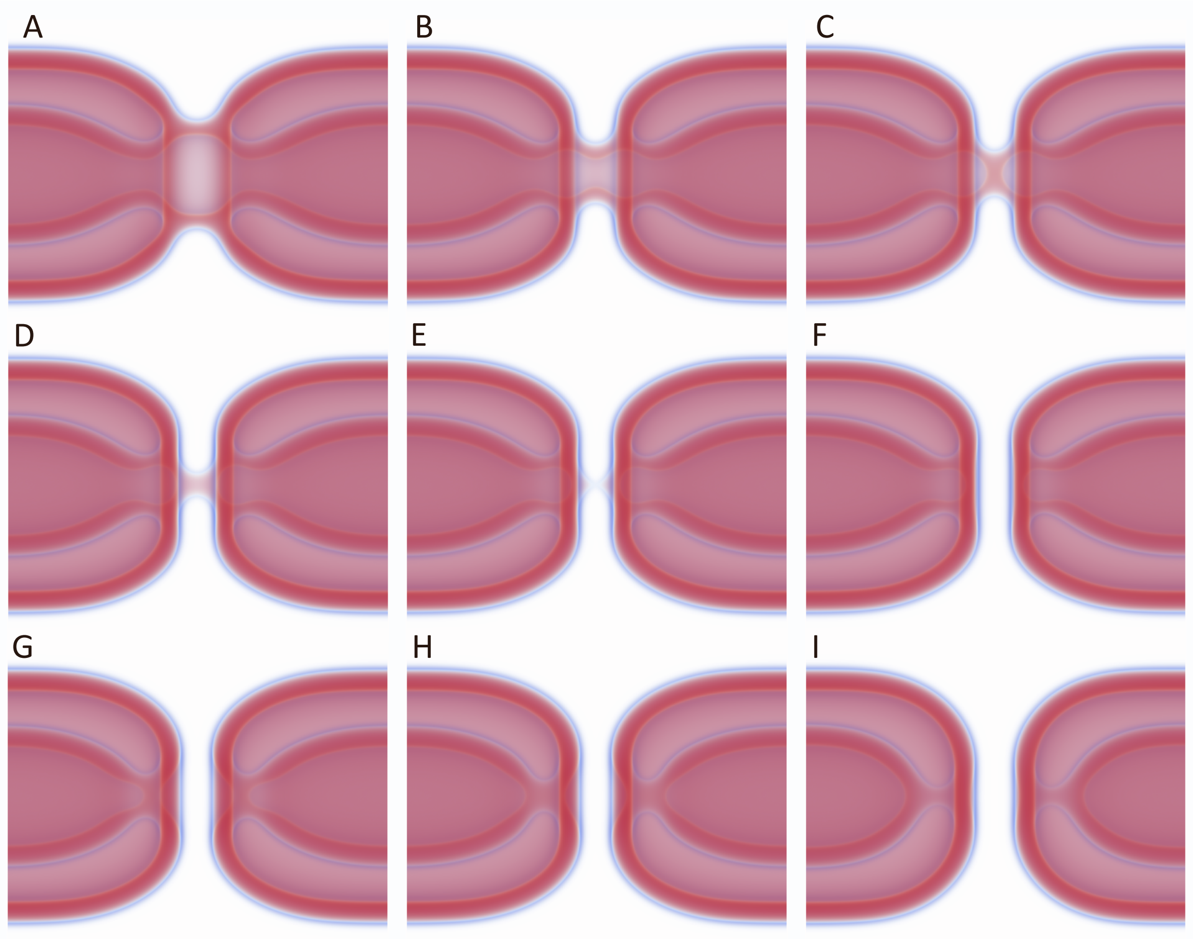

Figure S22: Collapse of the CH state via the canonical fission of the ST at $\sigma = 0.129\kappa/d^2$.

8. Mitra, K., I. Ubarretxena-Belandia, T. Taguchi, G. Warren, and D. M. Engelman, 2004. Modulation of the bilayer thickness of exocytic pathway membranes by membrane proteins rather than cholesterol. *Proceedings of the National Academy of Sciences* 101:4083–4088.

9. Discher, B. M., Y.-Y. Won, D. S. Ege, J. C.-M. Lee, F. S. Bates, D. E. Discher, and D. A. Hammer, 1999. Polymersomes: Tough Vesicles Made from Diblock Copolymers. *Science* 284:1143–1146.

10. Dimova, R., U. Seifert, B. Pouligny, S. Förster, and H.-G. Döbereiner, 2002. Hyperviscous diblock copolymer vesicles. *The European Physical Journal E* 7:241–250.
